# Alcohols as Substrates and Solvents for the Construction of 3-Alkoxylated-2-Oxindoles by Direct Alkoxylation of 3-Halooxindoles

**DOI:** 10.3390/molecules22050801

**Published:** 2017-05-13

**Authors:** Bing Lin, Zhi-Yong Chen, Huan-Huan Liu, Qi-Di Wei, Ting-Ting Feng, Ying Zhou, Can Wang, Xiong-Li Liu, Wei-Cheng Yuan

**Affiliations:** 1 Guizhou Medicine Edicine Edible Plant Resources Research and Development Center, College of Pharmacy, Guizhou University, Guiyang 550025, China; nlin@gzu.edu.cn (B.L.); dream198896@126.com (Z.-Y.C.); edithcl@sina.com (H.-H.L.); xuchwhj@163.com (Q.-D.W.); ttfeng@gzu.edu.cn (T.-T.F.); ftt0809@163.com (C.W.); 2 Key Laboratory for Asymmetric Synthesis & Chirotechnology of Sichuan Province, Chengdu Institute of Organic Chemistry, Chinese Academy of Sciences, Chengdu 610041, China; yuanweicheng@126.com

**Keywords:** 3-alkoxylated-2-oxindoles, 3-halooxindoles, alcohols, alkoxylation, environment-friendly chemistry

## Abstract

Described herein is an environmentally benign method for the synthesis of multisubstituted 3-alkoxylated-2-oxindoles **3** via direct alkoxylation of 3-halooxindoles **1**. A wide variety of such multisubstituted 3-alkoxylated-2-oxindole scaffold*s* were smoothly obtained in good yields (up to 94%) by heating in an oil bath at 35 °C for 24 h. A particularly valuable feature of this method was the development of environment-friendly chemistry using alcohols **2** as both the substrates and solvents in the presence of a catalytic amount of base.

## 1. Introduction

**Figure 1:**
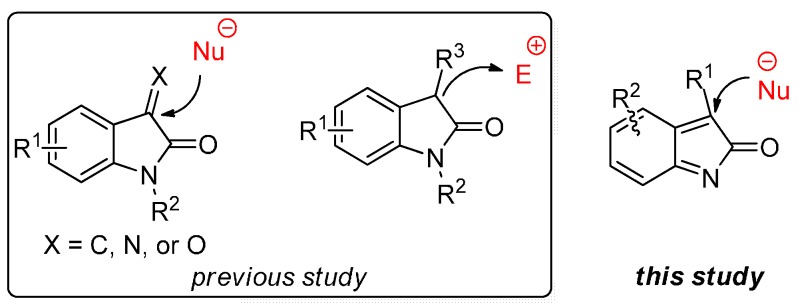
Strategies for the synthesis of 3,3′-disubstituted oxindoles.

3,3′-Disubstituted oxindoles are embedded in the indole alkaloids and various clinical pharmaceuticals [1,2,3,4,5,6,7,8,9,10,11]. Significant efforts have been devoted by many research groups to the synthesis of 3,3′-disubstituted oxindoles. Among them, the most important and efficient approaches involve the use of electrophilic isatins/isatinimines and nucleophilic 3-monosubstituted oxindoles for the synthesis of 3,3′-disubstituted oxindoles (Figure 1) [2,3,4,5,12,13]. Despite these advances, however, the use of indol-2-ones (generated in situ from 3-halooxindoles) as electrophiles has been limited [14,15,16,17,18,19,20,21,22,23,24,25,26,27,28].

**Figure 2:**
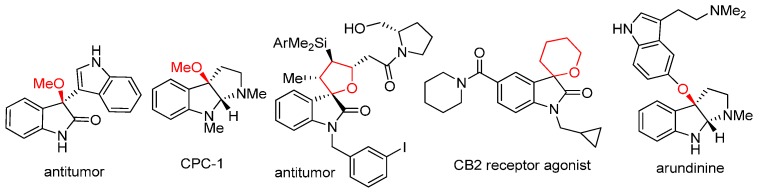
Representatives of the 3-alkoxylated-2-oxindolederivatives.

**Scheme 1:**
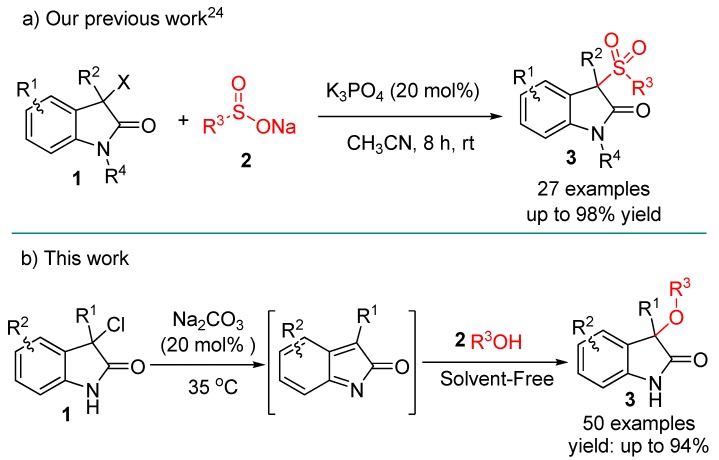
Construction of 3,3′-disubstituted oxindoles using 3-halooxindoles as electrophiles.

On the other hand, as 3,3′-disubstituted oxindole scaffolds, 3-alkoxylated-2-oxindoles possessing interesting structural properties were found in a number of biologically active synthetic and natural products (Figure 2) [29,30,31,32,33,34,35,36,37] Therefore, methods for alkoxylation of the oxindole nucleus are of value in medicinal chemistry and natural product synthesis. Over the past several years, although many synthetic methods have been developed for the synthesis of 3-hydroxy-2-oxindoles, existing catalytic syntheses of substituted 3-alkoxylated-2-oxindoles from simple substrates and catalysts are very few [38,39,40,41]. Worthy of note is that in 1964, Hinman and Bauman, et al. reported only a single example that described the synthesis of 3-methoxy-3-methyloxindole from 3-bromo-3-methyloxindole by treatment with 1 equivalent of NaHCO_3_ in MeOH/water (10/1). Prompted by this precedent, we have recently expanded the scope of this type of electrophile to synthesize a wide variety of 3-sulfonylated 3,3-disubstituted oxindole derivatives (Scheme 1) [21]. In this context, considering the high solubility of HCl in the alcoholic solvent, we supposed that a stoichiometric amount of base may be not necessary in alkoxylation of 3-halooxindoles in alcoholic solvents. We present herein the use of alcohols as both the substrates and solvents for the synthesis of 3-alkoxylated-2-oxindoles by direct alkoxylation of 3-halooxindoles using a catalytic amount of base.

## 2. Results and Discussion

In our initial endeavor, the 3-chloroxindole **1a** was prepared via a three-step approach (Knoevenagel condensation, reduction and chlorination) using benzaldehyde and 2-oxindole as the starting materials [17]. We then investigated 3-chloroxindole **1a** as a starting substrate to substantiate the feasibility of the strategy under various reaction conditions, as shown in Table 1. The tertiary amine catalyst DABCO (entry 1, Table 1) failed to afford the desired product **3aa**, providing an intractable product mixture from which no product could be identified by HRMS analysis. We then screened other different tertiary amines and inorganic bases (e.g., Et_3_N, DBU, Na_2_CO_3_, K_2_CO_3_ and NaHCO_3_) as catalysts in the reaction, and found that they can catalyse the reaction successfully leading to the desired product **3aa** in moderate to good yields (entries 2–6, Table 1). In the absence of catalyst, the reaction did not well occur under otherwise identical conditions, and only starting materials remained (Table 1, entry 7). Further solvent screening demonstrated that the reaction could deliver the product **3aa** preferentiallys (94% yield) with MeOH as the solvent (Table 1, entry 6). When the reaction was performed in EtOAc and THF, only 10% and 13% yields of **3aa** were obtained, respectively (Table 1, entries 10 and 11). Considering the environmental friendliness of this chemistry and good solubility of the substrates in alcohols, we chose to use alcohols as both the substrates and solvents in the alkoxylation reactions of 3-halooxindoles.Shortening the reaction time led to the desired product **3aa** in the relatively lower yields, along with some remaining starting materials (Table 1, entries 13 and 14). Further screening of the amount of the base demonstrated that the reaction could deliver the product **3aa** in 94% yield when a catalytic amount (20 mol %) of Na_2_CO_3_ was employed (Table 1, entry 6 and entries 15–17). Thus, the optimal reaction conditions we established were: 3-chloroxindole **1a** (0.40 mmol), 20 mol % of Na_2_CO_3_ (0.08 mmol) in 4.0 mL of MeOH **2a** in an oil bath at 35 °C for 24 h.

With the best reaction conditions in hands, we next turned our interest to the reaction scope, and the results are summarized in Table 2. MeOH (**2a**) was first used as a standard substrate to probe the reactivity of different 3-chloroxindoles **1** in this reaction. Significant structural variation in the oxindole system could be accommodated in this reaction. For example, electron-rich (Table 2, **3ba** and **3ca**) and electron-poor (Table 2, **3da**–**3ja**) substituents incorporated on the phenyl group or the benzo moiety of the oxindole core were perfectly tolerated under the conditions.

**Scheme 2:**
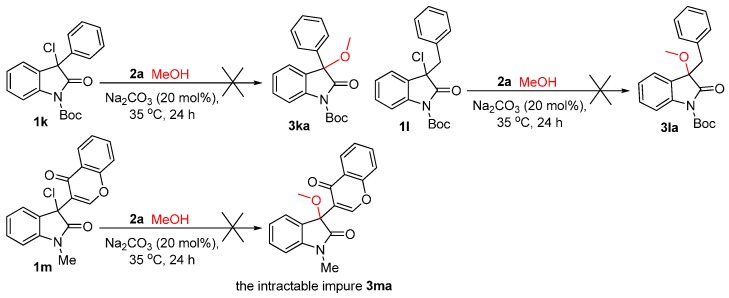
Contrast experiments with different types of substrate oxindoles **1** using MeOH as substrate and solvent.

The generality of the reaction was further demonstrated by using a variety of alcohols **2**, clearly indicating that all of the reactions proceeded smoothly under the optimal conditions, producing the desired products **3** in moderate to good yields (Table 2, **3ab**–**3ah**), regardless of the electronic nature of the chloroxindoles **1**. It is noteworthy that the bulky isopropyl alcohol (**2d**) led to deleterious effects on the reactivity, affording the desired products in moderate yields (Table 2, **3dd**–**3gd**). In addition, our attempts to identify the alkoxylation reactions of 3-aryl or 3-arylmethyl substituted *N*-Boc-oxindoles **1** using MeOH as substrate and solvent were in vain (Scheme 2). Furthermore, using 3-aryl substituted *N*-Me-oxindole **1m** as substrate it proved difficult to obtain the pure compound **1ma**, and we always obtained an intractable product mixture (Scheme 2). 

**Scheme 3:**
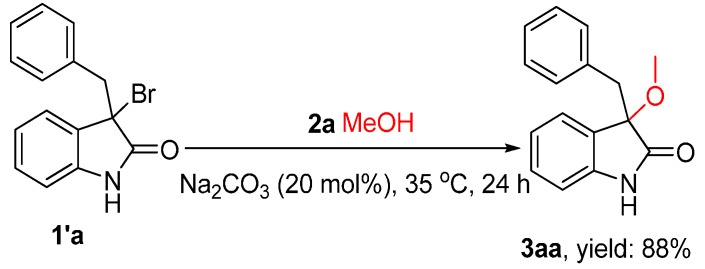
Bromooxindole **1**′**a** as a test substrate for this transformation.

In order to further explore the scope of the substrates, we also chose bromooxindole **1′a** as a test substrate for this transformation. To our delight, the reaction proceeded well to give the desired product **3aa** in good yield (88%) under the standard reaction conditions (Scheme 3).

**Scheme 4:**
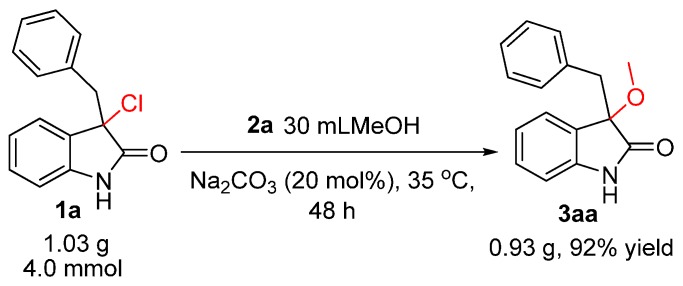
The alkoxylation of chloroxindole **1a** on a gram scale.

The significance and the high efficiency of the current protocol were demonstrated by a gram-scale synthesis of **3aa**. The alkoxylation of 3-chloroxindole **1a** proceeded cleanly on a 4.0 mmol scale (1.03 g of **1a**) in oil bath at 35 °C for 48 h. As outlined in Scheme 4, the corresponding adduct **3aa** was obtained smoothly in 92% yield, which was similar to those observed in a previous investigation (entry 1 of Table 2).

## 3. Experimental Section

### 3.1. General

The ^1^H and ^13^C NMR spectra were recorded on Bruker Avance DMX 400 MHz or 500 M NMR spectrometers (Bruker, Billerica, MA, USA) in CDCl_3_ using TMS as internal standard. Chemical shifts were reported as *δ* values (ppm). High-resolution mass spectra (HRMS-ESI) were obtained on a Micro™ Q-TOF Mass Spectrometer (Waters, Milford, MA, USA). Melting points were uncorrected and recorded on an Electothermal 9100 digital melting point apparatus (Electothermal, Stone, UK). Reagents were purchased from commercial sources and were used as received unless mentioned otherwise. Reactions were monitored by thin layer chromatography using silica gel GF_254_ plates. Column chromatography was performed on silica gel (300–400 mesh).

### 3.2. General Experimental Procedures for Synthesis of 3-Alkoxylated-2-Oxindoles ***3***

In an ordinary vial equipped with a magnetic stirring bar was added 3-chloroxindole **1** (0.4 mmol), 20 mol % of catalyst Na_2_CO_3_ (8.5 mg, 0.08 mmol) and 4.0 mL of alcohol **2**. The reaction mixture was stirred in oil bath at 35 °C for 24 h. After completion of the reaction, as indicated by TLC, the removal of solvent and purification by flash column chromatography (hexane/EtOAc = 10:1~6:1) was carried out to furnish the corresponding products **3**. 

### 3.3. Characterization Data of Compounds ***3***


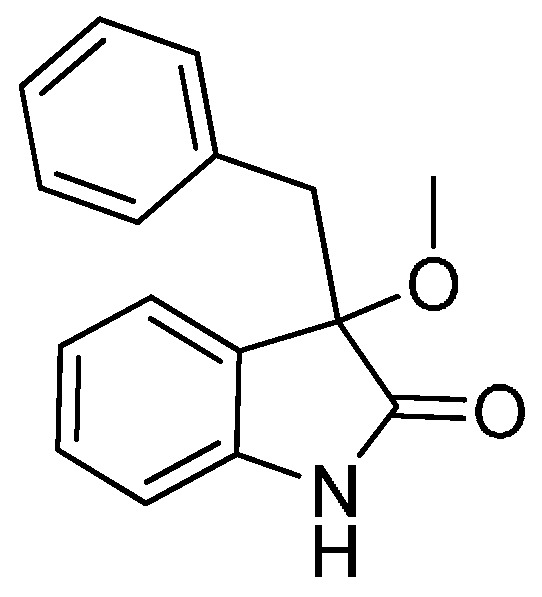


*3-Benzyl-3-methoxyindolin-2-one* (**3aa**). Light orange solid, m.p. 120.3–122.5 °C; yield 94%; ^1^H-NMR (CDCl_3_) δ: 3.08 (s, 3H), 3.11 (d, *J* = 12.8 Hz, 1H), 3.32 (d, *J* = 12.8 Hz, 1H), 6.80–6.83 (m, 1H), 6.93–6.96 (m, 2H), 7.02–7.11 (m, 5H), 7.22–7.26 (m, 1H), 9.13 (br s, 1H); ^13^C-NMR (CDCl_3_) δ: 43.6, 5.3.3, 84.2, 110.4, 122.6, 125.3, 126.3, 126.7, 127.6, 129.8, 130.6, 133.9, 141.2, 178.6; HRMS (ESI-TOF) *m*/*z*: Calcd. for C_16_H_15_NNaO_2_ [M + Na]^+^: 276.1000; Found: 276.1004. Spectra are in Supplementary Materials.


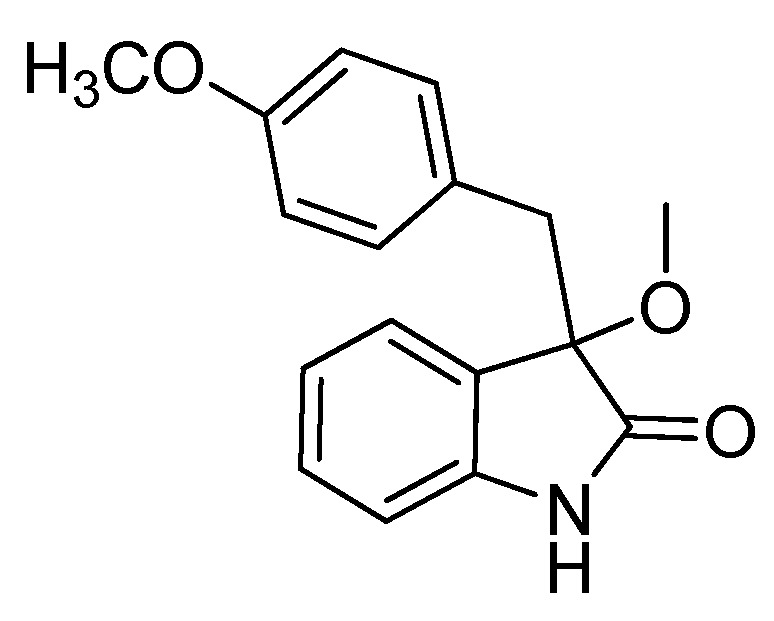


*3-Methoxy-3-(4-methoxybenzyl)indolin-2-one* (**3ba**). Light orange solid, m.p. 142.3–144.1 °C; yield 92%; ^1^H-NMR (CDCl_3_) δ: 3.03 (d, *J* = 12.8 Hz, 1H), 3.05 (s, 3H), 3.23 (d, *J* = 12.8 Hz, 1H), 3.66 (s, 3H), 6.57–6.61 (m, 2H), 6.79–6.85 (m, 3H), 7.01–7.03 (m, 2H), 7.20–7.25 (m, 1H), 8.98 (br s, 1H); ^13^C- NMR (CDCl_3_) δ: 42.9, 53.4, 55.1, 84.4, 110.5, 113.1, 122.7, 125.4, 126.0, 126.6, 129.9, 131.7, 141.4, 158.5, 178.8; HRMS (ESI-TOF) *m*/*z*: Calcd. for C_17_H_17_NNaO_3_ [M + Na]^+^: 306.1106; Found: 306.1107.


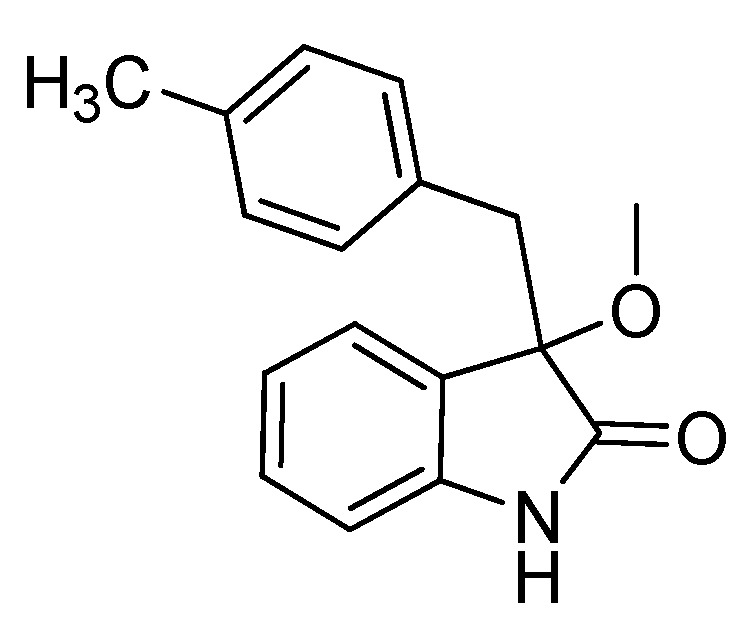


*3-Methoxy-3-(4-methylbenzyl)indolin-2-one* (**3ca**). Light orange solid, m.p. 130.7–134.2 °C; yield 91%; ^1^H-NMR (CDCl_3_) δ: 2.13 (s, 3H), 2.97–3.01 (m, 4H), 3.19 (d, *J* = 12.8 Hz, 1H), 6.72–6.81 (m, 5H), 6.94–6.99 (m, 2H), 7.13–7.18 (m, 1H), 8.94 (br s, 1H); ^13^C-NMR (CDCl_3_) δ: 21.0, 43.2, 53.2, 84.2, 110.4, 122.5, 125.3, 126.5, 128.3, 129.7, 130.4, 130.7, 136.2, 141.2, 178.7; HRMS (ESI-TOF) *m*/*z*: Calcd. for C_17_H_17_NNaO_2_ [M + Na]^+^: 290.1157; Found: 290.1154.


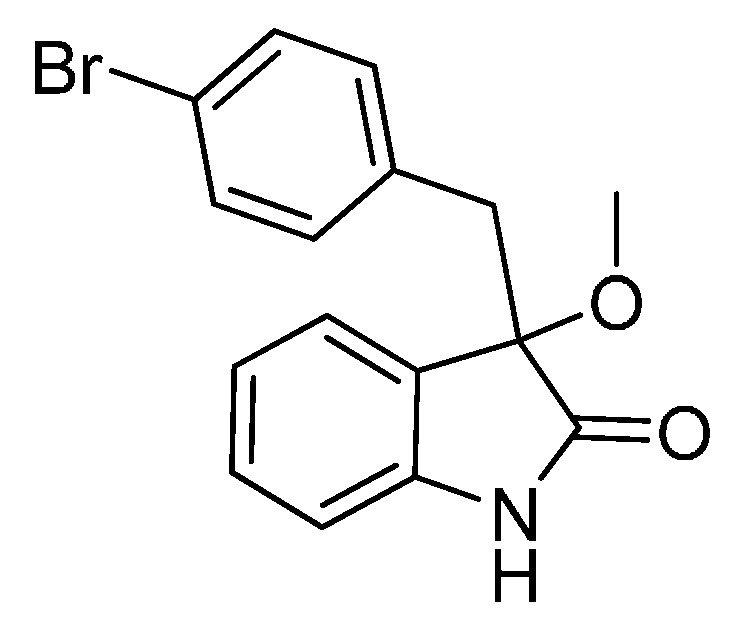


*3-(4-Bromobenzyl)-3-methoxyindolin-2-one* (**3da**). Light orange solid, m.p. 120.3–122.4 °C; yield 91%; ^1^H-NMR (CDCl_3_) δ: 3.05 (d, *J* = 13.2 Hz, 1H), 3.08 (s, 3H), 3.26 (d, *J* = 13.2 Hz, 1H), 6.81–6.84 (m, 3H), 7.03–7.06 (m, 2H), 7.20–7.26 (m, 3H), 8.82 (br s, 1H); ^13^C-NMR (CDCl_3_) δ: 43.0, 53.3, 83.8, 110.5, 121.0, 122.7, 125.2, 126.0, 130.0, 130.8, 132.3, 132.9, 141.0, 178.2; HRMS (ESI-TOF) *m*/*z*: Calcd. for C_16_H_14_BrNNaO_2_ [M + Na]^+^: 354.0106; Found: 354.0106.


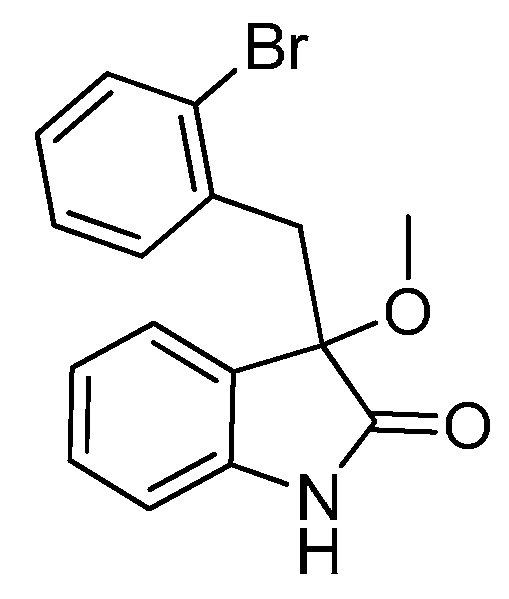


*3-(2-Bromobenzyl)-3-methoxyindolin-2-one* (**3ea**). Light orange solid, m.p. 184.5–187.1 °C; yield 91%; ^1^H- NMR (CDCl_3_) δ: 3.09 (s, 3H), 3.36 (d, *J* = 11.2 Hz, 1H), 3.48 (d, *J* = 11.2 Hz, 1H), 6.68 (d, *J* = 6.0 Hz, 1H), 6.89–6.95 (m, 2H), 7.05–7.09 (m, 1H), 7.20–7.27 (m, 2H), 7.40–7.42 (m, 1H), 7.48–7.50 (m, 1H), 9.32 (br s, 1H); ^13^C-NMR (CDCl_3_) δ: 42.0, 53.2, 83.2, 110.5, 122.6, 125.6, 126.8, 128.5, 129.8, 132.4, 132.8, 134.4, 140.9, 179.0; HRMS (ESI-TOF) *m*/*z*: Calcd. for C_16_H_14_BrNNaO_2_ [M + Na]^+^: 354.0106; Found: 354.0105.


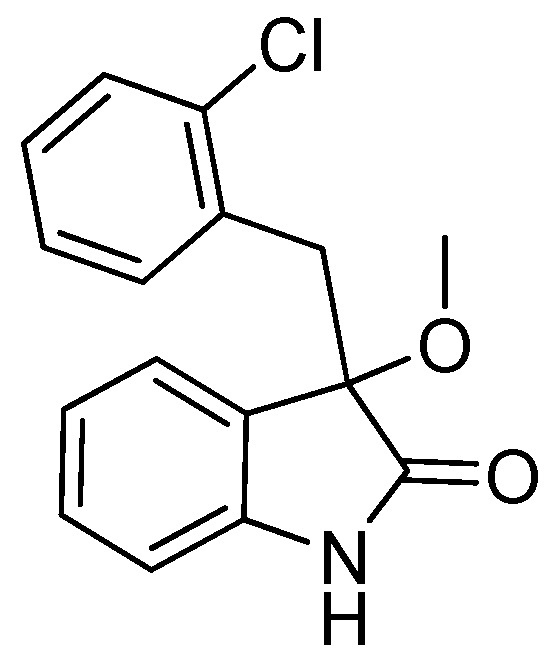


*3-(2-Chlorobenzyl)-3-methoxyindolin-2-one* (**3fa**). Light orange solid, m.p. 138.2–139.8 °C; yield 93%; ^1^H-NMR (CDCl_3_) δ: 3.09 (s, 3H), 3.35 (d, *J* = 13.5 Hz, 1H), 3.47 (d, *J* = 13.5 Hz, 1H), 6.77 (d, *J* = 7.2 Hz, 1H), 6.89–6.97 (m, 2H), 7.13–7.26 (m, 4H), 7.42–7.44 (m, 1H), 9.40 (br s, 1H); ^13^C-NMR (CDCl_3_) δ: 39.4, 53.2, 83.3, 110.4, 122.6, 125.5, 125.9, 126.2, 128.3, 129.1, 129.8, 132.5, 132.7, 135.3, 140.5, 141.0, 179.0; HRMS (ESI-TOF) *m*/*z*: Calcd. for C_16_H_14_ClNNaO_2_ [M + Na]^+^: 310.0611; Found: 310.0614.


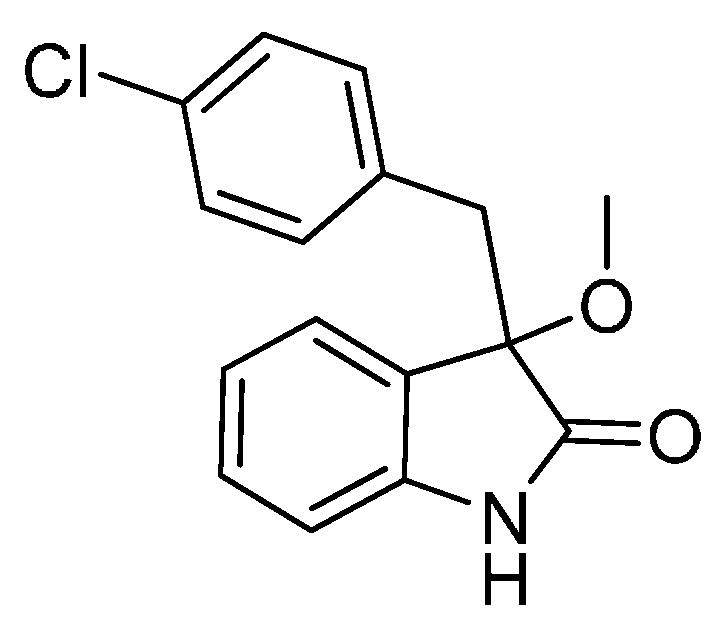


*3-(4-Chlorobenzyl)-3-methoxyindolin-2-one* (**3ga**). Light orange solid, m.p. 125.2–127.3 °C; yield 91%; ^1^H-NMR (CDCl_3_) δ: 3.05 (d, *J* = 12.8 Hz, 1H), 3.08 (s, 3H), 3.27 (d, *J* = 12.8 Hz, 1H), 6.83 (d, *J* = 7.6 Hz, 1H), 6.88 (d, *J* = 8.4 Hz, 2H), 7.02–7.08 (m, 4H), 7.24–7.28 (m, 1H), 8.93 (br s, 1H); ^13^C-NMR (CDCl_3_) δ: 42.9, 53.3, 83.9, 110.5, 122.8, 125.3, 126.1, 127.8, 130.0, 131.9, 132.4, 132.8, 141.1, 178.3; HRMS (ESI-TOF) *m*/*z*: Calcd. for C_16_H_14_ClNNaO_2_ [M + Na]^+^: 310.0611; Found: 310.0610.


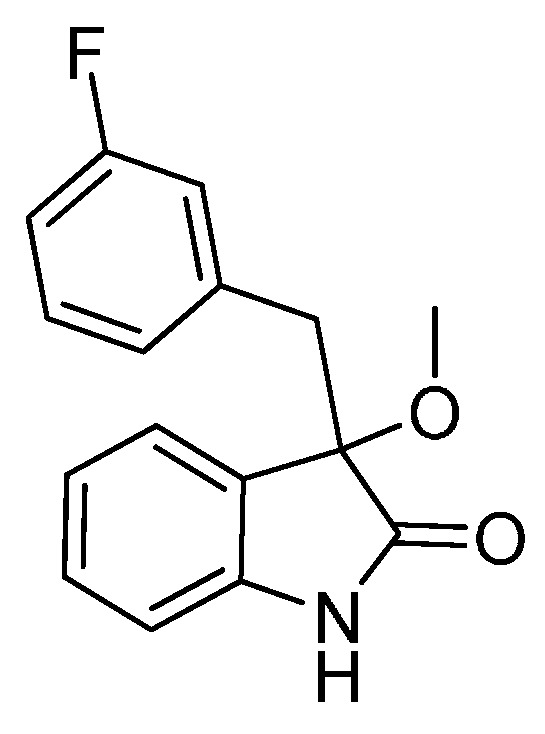


*3-(3-Fluorobenzyl)-3-methoxyindolin-2-one* (**3ha**). Light orange solid, m.p. 135.7–138.2 °C; yield 90%; ^1^H-NMR (CDCl_3_, 500 MHz) δ: 3.07 (d, *J* = 13.5 Hz, 1H), 3.09 (s, 3H), 3.31 (d, *J* = 13.5 Hz, 1H), 6.70–6.75 (m, 2H), 6.83–6.85 (m, 2H), 6.98–7.06 (m, 3H), 7.24–7.27 (m, 1H), 9.12 (br s, 1H); ^13^C-NMR (CDCl_3_, 125 MHz) δ: 43.2, 53.3, 83.8, 110.5, 110.6, 113.7 (d, *J_CF_* = 20.8 Hz), 117.4 (d, *J_CF_* = 21.3 Hz), 122.7, 125.3, 126.0, 126.3, 126.4, 128.9, 129.0, 130.0, 136.5, 136.6, 141.1, 141.2, 162.1 (d, *J_CF_* = 245.8 Hz), 178.5; HRMS (ESI-TOF) *m*/*z*: Calcd. for C_16_H_14_FNNaO_2_ [M + Na]^+^: 294.0906; Found: 294.0908.


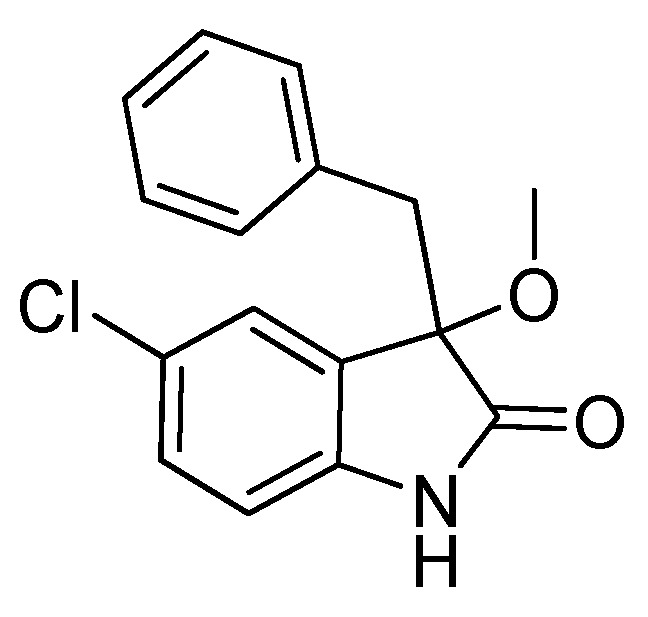


*3-Benzyl-5-chloro-3-methoxyindolin-2-one* (**3ia**). Light orange solid, m.p. 130.4–133.3 °C; yield 90%; ^1^H-NMR (CDCl_3_) δ: 3.00 (s, 3H), 3.03 (d, *J* = 12.8 Hz, 1H), 3.21 (d, *J* = 12.8 Hz, 1H), 6.66 (d, *J* = 8.4 Hz, 1H), 6.87–6.92 (m, 3H), 7.01–7.08 (m, 3H), 7.13–7.19 (m, 1H), 8.83 (br s, 1H); ^13^C-NMR (CDCl_3_) δ: 43.6, 53.5, 84.3, 111.4, 125.6, 127.0, 127.8, 128.2, 128.3, 129.8, 130.5, 133.4, 139.6, 178.2; HRMS (ESI-TOF) *m*/*z*: Calcd. for C_16_H_14_ClNNaO_2_ [M + Na]^+^: 310.0611; Found: 310.0611.


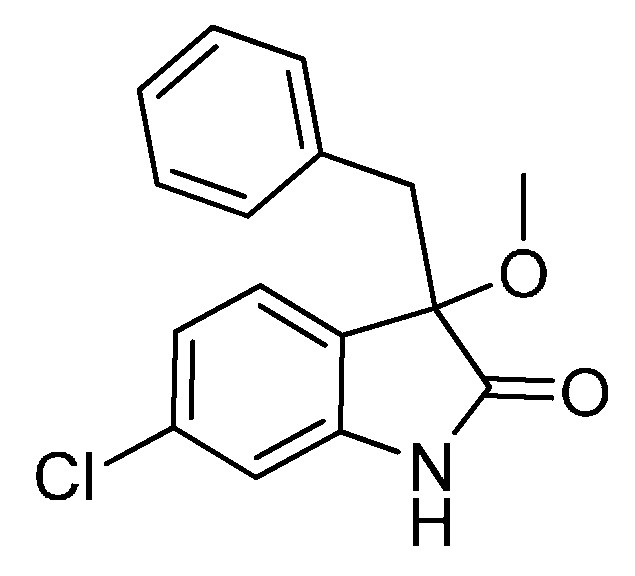


*3-Benzyl-6-chloro-3-methoxyindolin-2-one* (**3ja**). Light orange solid, m.p. 198.4–201.9 °C; yield 91%; ^1^H-NMR (DMSO-*d*_6_) δ: 2.91 (s, 3H), 3.00 (d, *J* = 12.8 Hz, 1H), 3.20 (d, *J* = 12.8 Hz, 1H), 6.66 (s, 1H), 6.67–6.90 (m, 2H), 7.03 (d, *J* = 2.0 Hz, 1H), 7.09–7.13 (m, 4H), 10.5 (br s, 1H); ^13^C-NMR (DMSO-*d*_6_) δ: 42.3, 52.3, 83.1, 109.9, 121.5, 125.0, 126.6, 126.8, 127.7, 130.3, 134.0, 134.1, 143.9, 176.2; HRMS (ESI-TOF) *m*/*z*: Calcd. for C_16_H_14_ClNNaO_2_ [M + Na]^+^: 310.0611; Found: 310.0611.


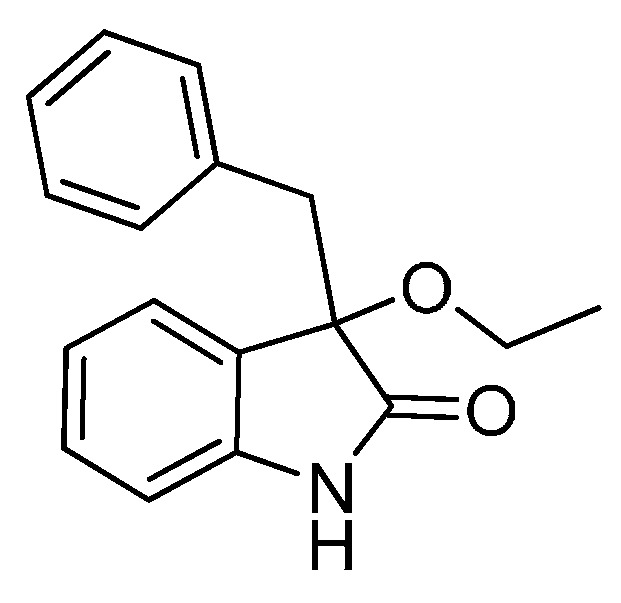


*3-Benzyl-3-ethoxyindolin-2-one* (**3ab**). Light orange solid, m.p. 112.0–113.8 °C; yield 89%; ^1^H-NMR (CDCl_3_) δ: 1.14–1.18 (m, 3H), 3.10–3.15 (m, 2H), 3.22–3.26 (m, 1H), 3.31 (d, *J* = 12.8 Hz, 1H), 6.78 (d, *J* = 7.6 Hz, 1H), 6.92–6.93 (m, 2H), 6.94–7.10 (m, 5H), 7.20–7.26 (m, 1H), 9.01 (br s, 1H); ^13^C- NMR (CDCl_3_) δ: 15.3, 43.8, 61.2, 83.6, 110.3, 122.5, 125.1, 126.7, 127.1, 127.6, 129.6, 130.5, 134.0, 141.0, 179.0; HRMS (ESI-TOF) *m*/*z*: Calcd. for C_17_H_17_NNaO_2_ [M + Na]^+^: 290.1157; Found: 290.1154.


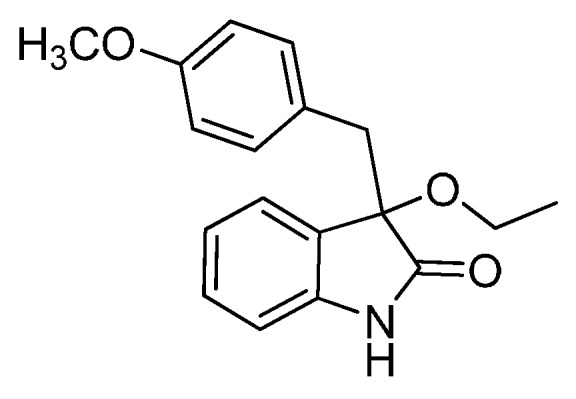


*3-Ethoxy-3-(4-methoxybenzyl)indolin-2-one* (**3bb**). Light orange solid, m.p. 151.2–153.1°C; yield 92%; ^1^H-NMR (CDCl_3_) δ: 1.12–1.16 (m, 3H), 3.02–3.12 (m, 2H), 3.19–3.26 (m, 2H), 3.65 (s, 3H), 6.56–6.58 (m, 2H), 6.76–6.84 (m, 3H), 7.01–7.06 (m, 2H), 7.18–7.25 (m, 1H), 8.93 (br s, 1H); ^13^C-NMR (CDCl_3_) δ: 15.5, 43.1, 55.1, 61.3, 83.8, 110.4, 113.1, 122.6, 125.2, 126.1, 127.5, 129.7, 131.6, 141.2, 158.4, 179.1; HRMS (ESI-TOF) *m*/*z*: Calcd. for C_18_H_19_NNaO_3_ [M + Na]^+^: 320.1263; Found: 320.1263.


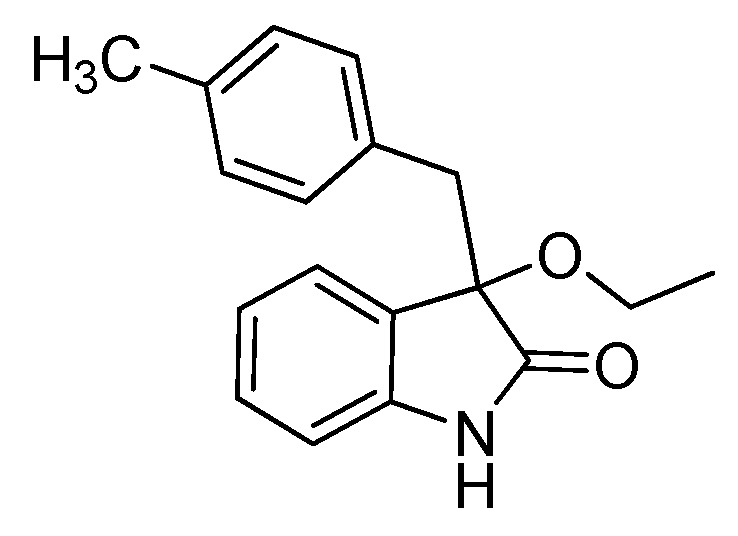


*3-Ethoxy-3-(4-methylbenzyl)indolin-2-one* (**3cb**). Light orange solid, m.p. 138.8–141.9 °C; yield 91%; ^1^H-NMR (CDCl_3_) δ: 1.07–1.10 (m, 3H), 2.13 (s, 3H), 2.99–3.07 (m, 2H), 3.14–3.22 (m, 2H), 6.69–6.79 (m, 5H), 6.94–7.01 (m, 2H), 7.12–7.18 (m, 1H), 8.82 (br s, 1H); ^13^C-NMR (CDCl_3_) δ: 15.3, 21.0, 43.4, 61.1, 83.6, 110.2, 122.5, 125.1, 127.3, 128.3, 129.6, 130.4, 130.8, 136.1, 141.0, 178.9; HRMS (ESI-TOF) *m*/*z*: Calcd. for C_18_H_19_NNaO_2_ [M + Na]^+^: 304.1313; Found: 304.1315.


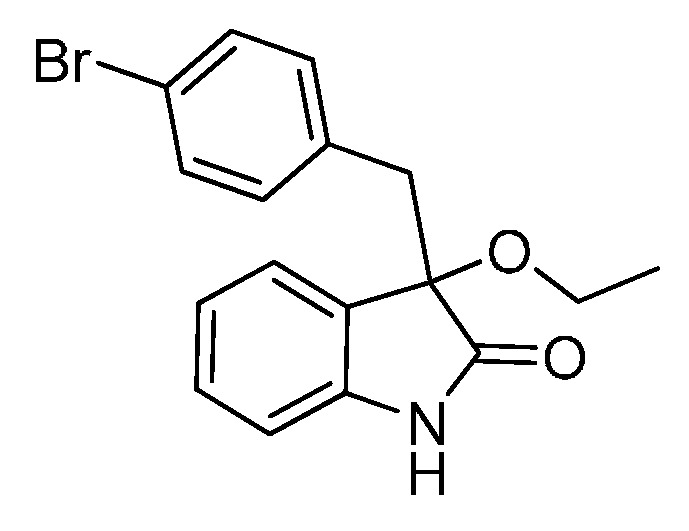


*3-(4-Bromobenzyl)-3-ethoxyindolin-2-one* (**3db**). Light orange solid, m.p. 142.1–144.6 °C; yield 90%; ^1^H-NMR (CDCl_3_) δ: 1.15–1.18 (m, 3H), 3.04 (d, *J* = 13.2 Hz, 1H), 3.11–3.15 (m, 1H), 3.22–3.28 (m, 2H), 6.78–6.84 (m, 3H), 7.03–7.05 (m, 2H), 7.18–7.27 (m, 3H), 8.82 (br s, 1H); ^13^C-NMR (CDCl_3_) δ: 15.3, 43.2, 61.3, 83.2, 110.4, 120.9, 122.7, 125.1, 126.8, 129.8, 130.7, 132.3, 133.0, 140.8, 178.6; HRMS (ESI-TOF) *m*/*z*: Calcd. for C_17_H_16_BrNNaO_2_ [M + Na]^+^: 368.0262; Found: 368.0265.


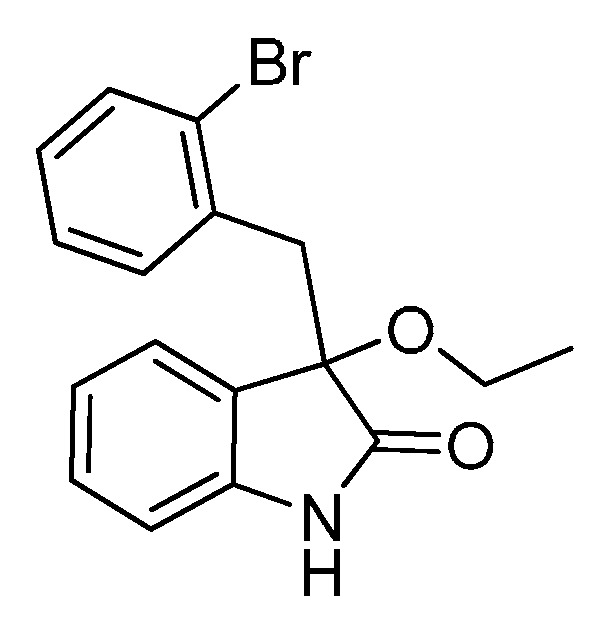


*3-(2-Bromobenzyl)-3-ethoxyindolin-2-one* (**3eb**). Light orange solid, m.p. 185.1–186.2 °C; yield 88%; ^1^H-NMR (CDCl_3_) δ: 1.14–1.18 (m, 3H), 3.07–3.11 (m, 1H), 3.23–3.28 (m, 1H), 3.32 (d, *J* = 13.6 Hz, 1H), 3.47 (d, *J* = 13.6 Hz, 1H), 6.65 (d, *J* = 7.2 Hz, 1H), 6.86–6.92 (m, 2H), 7.05–7.07 (m, 1H), 7.18–7.25 (m, 2H), 7.38–7.40 (m, 1H), 7.49–7.52 (m, 1H); ^13^C-NMR (CDCl_3_) δ: 15.4, 42.2, 61.2, 82.7, 110.5, 122.6, 125.6, 126.5, 126.7, 126.9, 128.6, 129.7, 132.4, 132.9, 134.7, 140.9, 179.4; HRMS (ESI-TOF) *m*/*z*: Calcd. for C_17_H_16_BrNNaO_2_ [M + Na]^+^: 368.0262; Found: 368.0262.


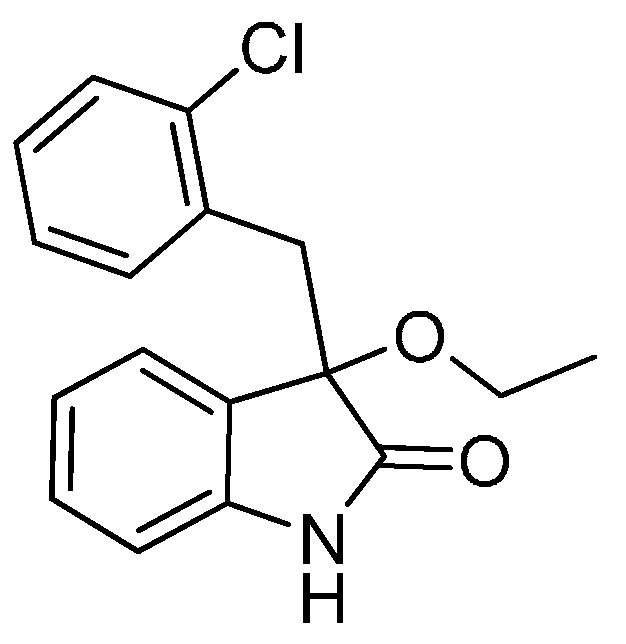


*3-(2-Chlorobenzyl)-3-ethoxyindolin-2-one* (**3fb**). Light orange solid, m.p. 207.1–209.2 °C; yield 90%; ^1^H-NMR (DMSO-*d*_6_) δ: 1.01–1.05 (m, 3H), 2.91–2.95 (m, 1H), 3.05–3.08 (m, 1H), 3.14 (d, *J* = 12.8 Hz, 1H), 3.32 (d, *J* = 12.8 Hz, 1H), 6.72 (d, *J* = 7.8 Hz, 1H), 6.80–6.87 (m, 2H), 7.14–7.28 (m, 5H), 10.5 (br s, 1H); ^13^C-NMR (DMSO-*d*_6_) δ: 15.3, 40.1, 60.0, 82.0, 109.8, 121.5, 124.8, 126.5, 128.6, 129.0, 129.7, 132.2, 132.5, 142.0, 176.7; HRMS (ESI-TOF) *m*/*z*: Calcd. for C_17_H_16_ClNNaO_2_ [M + Na]^+^: 324.0767; Found: 324.0769.


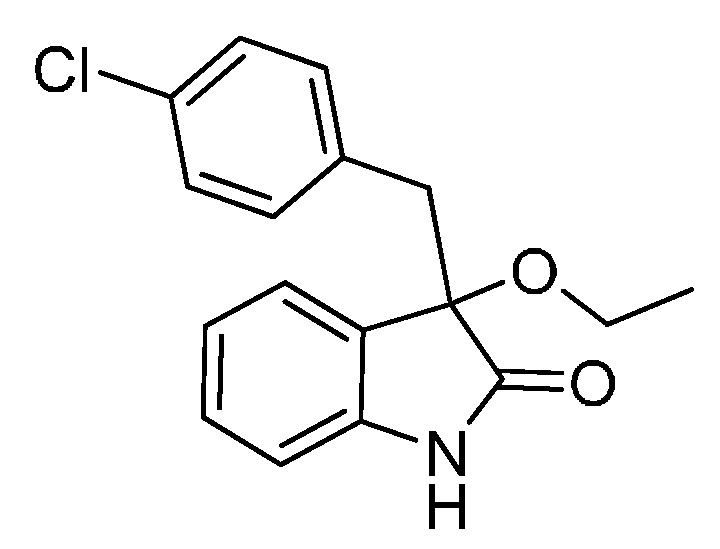


*3-(4-Chlorobenzyl)-3-ethoxyindolin-2-one* (**3gb**). Light orange solid, m.p. 154.1–156.2 °C; yield 91%; ^1^H-NMR (CDCl_3_) δ: 1.14–1.18 (m, 3H), 3.06 (d, *J* = 12.8 Hz, 1H), 3.11–3.15 (m, 1H), 3.21–3.25 (m, 1H), 3.28 (d, *J* = 12.8 Hz, 1H), 6.81 (d, *J* = 8.0 Hz, 1H), 6.88 (d, *J* = 8.4 Hz, 2H), 7.02–7.05 (m, 4H), 7.22–7.27 (m, 1H), 9.04 (br s, 1H); ^13^C-NMR (CDCl_3_) δ: 15.3, 43.1, 61.3, 83.3, 110.4, 122.7, 125.1, 126.9, 127.7, 129.8, 131.9, 132.5, 132.7, 140.9, 178.7; HRMS (ESI-TOF) *m*/*z*: Calcd. for C_17_H_16_ClNNaO_2_ [M + Na]^+^: 324.0767; Found: 324.0766.


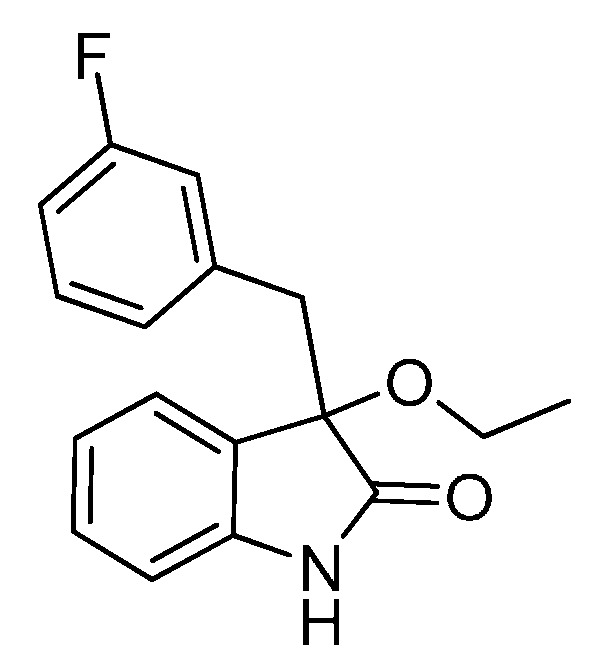


*3-Ethoxy-3-(3-fluorobenzyl)indolin-2-one* (**3hb**). Light orange solid, m.p. 118.3–119.8 °C; yield 89%; ^1^H-NMR (CDCl_3_, 500 MHz) δ: 1.16–1.19 (m, 3H), 3.08 (d, *J* = 13.0 Hz, 1H), 3.12–3.15 (m, 1H), 3.24–3.27 (m, 1H), 3.31 (d, *J* = 13.0 Hz, 1H), 6.69–6.75 (m, 2H), 6.80–6.83 (m, 2H), 7.00–7.05 (m, 3H), 7.22–7.26 (m, 1H), 9.10 (br s, 1H); ^13^C-NMR (CDCl_3_, 125 MHz) δ: 15.3, 43.4, 61.2, 83.2, 110.4, 110.5, 113.6, 113.7, 117.4 (d, *J_CF_* = 21.3 Hz), 122.7, 125.1, 126.3, 126.4, 126.8, 128.9 (d, *J_CF_* = 8.8 Hz), 129.8, 136.6 (d, *J_CF_* = 7.5 Hz), 140.9, 162.1 (d, *J_CF_* = 243.8 Hz), 178.7; HRMS (ESI-TOF) *m*/*z*: Calcd. for C_17_H_16_FNNaO_2_ [M + Na]^+^: 308.1063; Found: 308.1067.


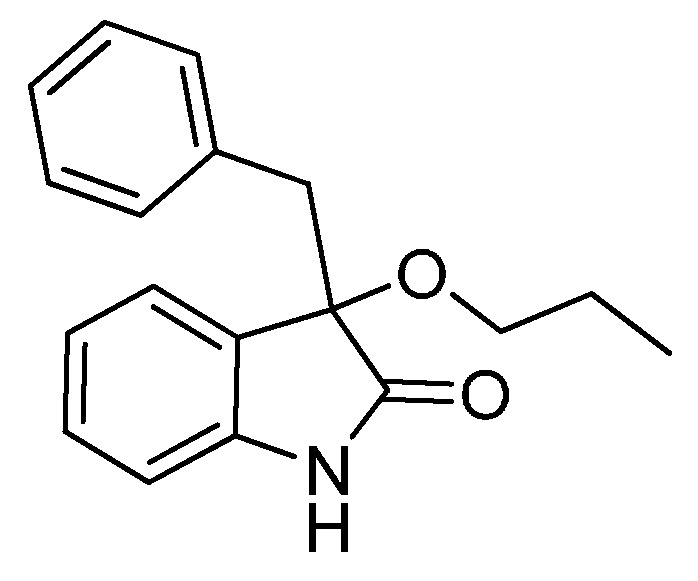


*3-Benzyl-3-propoxyindolin-2-one* (**3ac**). Light orange solid, m.p. 146.1–148.3 °C; yield 86%; ^1^H-NMR (CDCl_3_) δ: 0.84–0.88 (m, 3H), 1.54–1.60 (m, 2H), 2.95–3.01 (m, 1H), 3.08 (d, *J* = 12.8 Hz, 1H), 3.15–3.20 (m, 1H), 3.33 (d, *J* = 12.8 Hz, 1H), 6.78 (d, *J* = 7.6 Hz, 1H), 6.96–7.13 (m, 7H), 7.19–7.26 (m, 1H), 8.90 (br s, 1H); ^13^C-NMR (CDCl_3_) δ: 10.5, 23.1, 43.8, 67.2, 83.4, 110.2, 122.5, 125.3, 126.7, 127.2, 127.6, 129.6, 130.7, 134.1, 140.9, 178.9; HRMS (ESI-TOF) *m*/*z*: Calcd. for C_18_H_19_NNaO_2_ [M + Na]^+^: 304.1313; Found: 304.1315.


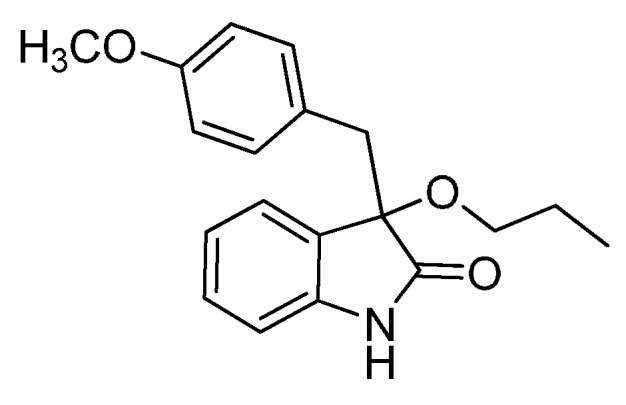


*3-(4-Methoxybenzyl)-3-propoxyindolin-2-one* (**3bc**). Light orange solid, m.p. 145.5–146.9 °C; yield 82%; ^1^H-NMR (CDCl_3_) δ: 0.81–0.85 (m, 3H), 1.52–1.56 (m, 2H), 2.94–3.02 (m, 2H), 3.12–3.15 (m, 1H), 3.25 (d, *J* = 12.8 Hz, 1H), 3.66 (s, 3H), 6.57–6.60 (m, 2H), 6.78 (d, *J* = 7.6 Hz, 1H), 6.84–6.87 (m, 2H), 6.97–7.00 (m, 2H), 7.19–7.25 (m, 1H), 8.99 (br s, 1H); ^13^C-NMR (CDCl_3_) δ: 10.7, 23.2, 43.0, 55.1, 67.3, 83.6, 110.4, 113.1, 122.6, 125.4, 126.2, 127.5, 129.7, 131.7, 141.2, 158.4, 179.3; HRMS (ESI-TOF) *m*/*z*: Calcd. for C_19_H_21_NNaO_3_ [M + Na]^+^: 334.1419; Found: 334.1415.


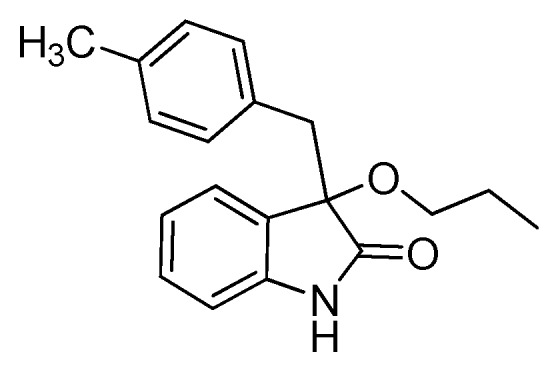


*3-(4-Methylbenzyl)-3-propoxyindolin-2-one* (**3cc**). Light orange solid, m.p. 75.1–76.8 °C; yield 83%; ^1^H-NMR (CDCl_3_) δ: 0.76–0.80 (m, 3H), 1.46–1.53 (m, 2H), 2.15 (s, 3H), 2.89–2.93 (m, 1H), 2.98 (d, *J* = 12.8 Hz, 1H), 3.08–3.11 (m, 1H), 3.21 (d, *J* = 12.8 Hz, 1H), 6.68 (d, *J* = 7.6 Hz, 1H), 6.76–6.82 (m, 4H), 6.92–6.97 (m, 2H), 7.12–7.19 (m, 1H), 8.41 (br s, 1H); ^13^C-NMR (CDCl_3_) δ: 10.5, 21.0, 23.1, 43.4, 67.2, 83.3, 110.1, 122.4, 125.3, 127.4, 128.3, 129.5, 130.5, 130.9, 136.1, 140.9, 178.7; HRMS (ESI-TOF) *m*/*z*: Calcd. for C_19_H_21_NNaO_2_ [M + Na]^+^: 318.1470; Found: 318.1472.


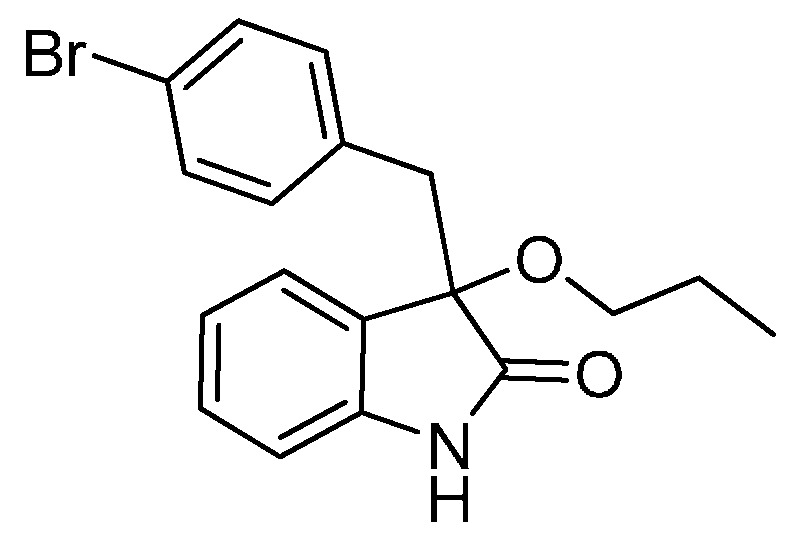


*3-(4-Bromobenzyl)-3-propoxyindolin-2-one* (**3dc**). Light orange solid, m.p. 143.3–144.8 °C; yield 82%; ^1^H-NMR (CDCl_3_) δ: 0.84–0.87 (m, 3H), 1.53–1.59 (m, 2H), 2.95–3.02 (m, 2H), 3.14–3.19 (m, 1H), 3.27 (d, *J* = 12.8 Hz, 1H), 6.81–6.86 (m, 3H), 6.94–6.96 (m, 1H), 7.01–7.04 (m, 1H), 7.20–7.24 (m, 3H), 8.88 (br s, 1H); ^13^C-NMR (CDCl_3_) δ: 10.5, 23.0, 43.1, 67.2, 83.0, 110.4, 120.9, 122.6, 125.2, 126.9, 129.8, 130.7, 132.4, 133.2, 140.8, 178.7; HRMS (ESI-TOF) *m*/*z*: Calcd. for C_18_H_18_BrNNaO_2_ [M + Na]^+^: 382.0419; Found: 382.0417.


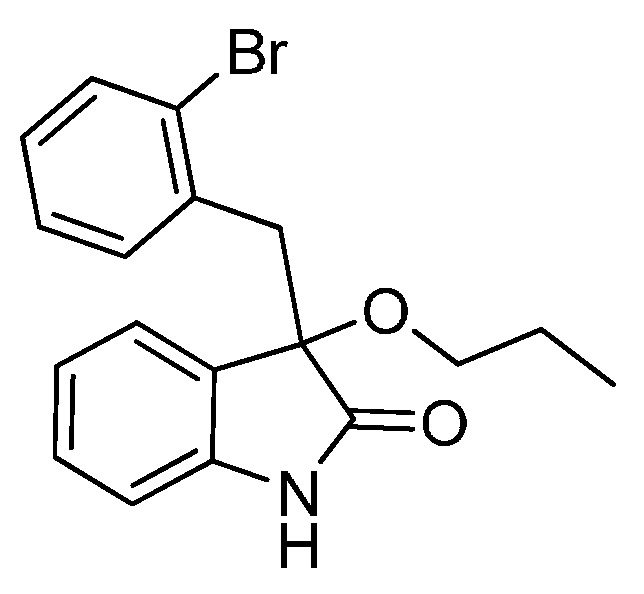


*3-(2-Bromobenzyl)-3-propoxyindolin-2-one* (**3ec**). Light orange solid, m.p. 153.1–154.9 °C; yield 81%; ^1^H-NMR (CDCl_3_) δ: 0.85–0.88 (m, 3H), 1.54–1.62 (m, 2H), 2.95 (d, *J* = 6.0 Hz, 1H), 3.21 (d, *J* = 6.4 Hz, 1H), 3.32 (d, *J* = 11.2 Hz, 1H), 3.48 (d, *J* = 11.2 Hz, 1H), 6.59 (d, *J* = 6.0 Hz, 1H), 6.89–6.92 (m, 2H), 7.07–7.09 (m, 1H), 7.21–7.26 (m, 2H), 7.41 (d, *J* = 6.4 Hz, 1H), 7.56 (d, *J* = 6.0 Hz, 1H), 9.22 (br s, 1H); ^13^C-NMR (CDCl_3_) δ: 10.6, 23.1, 42.1, 67.0, 82.3, 110.3, 122.5, 125.5, 126.5, 126.6, 126.7, 128.4, 129.6, 132.3, 133.0, 134.7, 140.7, 179.2; HRMS (ESI-TOF) *m*/*z*: Calcd. for C_18_H_18_BrNNaO_2_ [M + Na]^+^: 382.0419; Found: 382.0421.


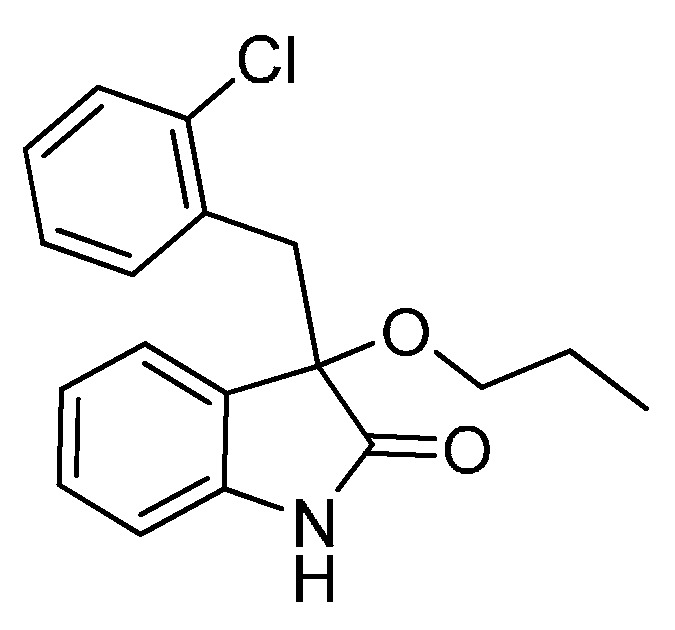


*3-(2-Chlorobenzyl)-3-propoxyindolin-2-one* (**3fc**). Light orange solid, m.p. 160.3–161.9 °C; yield 80%; ^1^H-NMR (CDCl_3_) δ: 0.85–0.88 (m, 3H), 1.56–1.62 (m, 2H), 2.94–2.98 (m, 1H), 3.18–3.22 (m, 1H), 3.33 (d, *J* = 13.6 Hz, 1H), 3.46 (d, *J* = 13.6 Hz, 1H), 6.69 (d, *J* = 7.2 Hz, 1H), 6.86–6.94 (m, 2H), 7.13–7.26 (m, 4H), 7.51 (d, *J* = 4.0 Hz, 1H), 9.15 (br s, 1H); ^13^C-NMR (CDCl_3_) δ: 10.6, 23.1, 39.6, 67.1, 82.4, 110.2, 122.5, 125.4, 126.0, 126.7, 128.2, 129.0, 129.6, 132.8, 135.3, 140.8, 179.2; HRMS (ESI-TOF) *m*/*z*: Calcd. for C_18_H_18_ClNNaO_2_ [M + Na]^+^: 338.0924; Found: 338.0924.


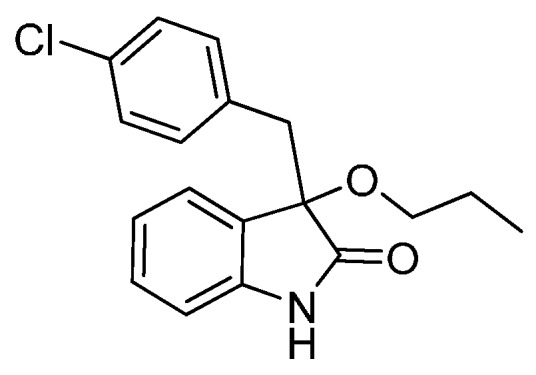


*3-(4-Chlorobenzyl)-3-propoxyindolin-2-one* (**3gc**). Light orange solid, m.p. 158.1–161.2 °C; yield 84%; ^1^H-NMR (CDCl_3_) δ: 0.84–0.87 (m, 3H), 1.53–1.60 (m, 2H), 2.96–3.00 (m, 1H), 3.03 (d, *J* = 12.8 Hz, 1H), 3.14–3.18 (m, 1H), 3.29 (d, *J* = 12.8 Hz, 1H), 6.81 (d, *J* = 7.6 Hz, 1H), 6.89–6.97 (m, 3H), 7.01–7.07 (m, 3H), 7.22–7.26 (m, 1H), 8.90 (br s, 1H); ^13^C-NMR (CDCl_3_) δ: 10.5, 23.0, 43.1, 67.3, 83.0, 110.3, 122.6, 125.2, 126.9, 127.7, 129.8, 132.0, 132.7, 140.8, 178.7; HRMS (ESI-TOF) *m*/*z*: Calcd. for C_18_H_18_ClNNaO_2_ [M + Na]^+^: 338.0924; Found: 338.0927.


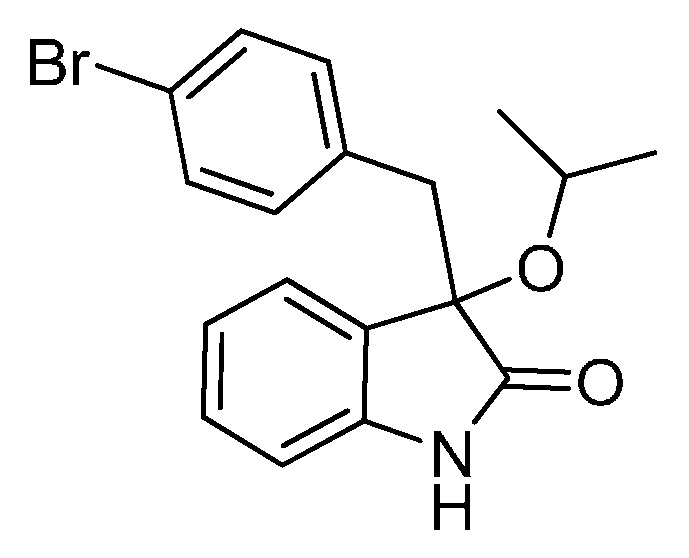


*3-(4-Bromobenzyl)-3-isopropoxyindolin-2-one* (**3dd**). Light orange solid, m.p. 148.1–150.1 °C; yield 77%; ^1^H-NMR (CDCl_3_, 500 MHz) δ: 1.01 (d, *J* = 6.5 Hz, 3H), 1.10 (d, *J* = 6.0 Hz, 3H), 2.97 (d, *J* = 13.0 Hz, 1H), 3.23 (d, *J* = 13.0 Hz, 1H), 6.78–6.83 (m, 3H), 6.98–7.02 (m, 2H), 7.19–7.27 (m, 3H), 8.57 (br s, 1H); ^13^C-NMR (CDCl_3_, 125 MHz) δ: 23.1, 24.1, 43.8, 69.5, 82.7, 110.3, 120.9, 122.4, 125.6, 127.3, 129.8, 130.6, 132.4, 133.2, 140.6, 179.3; HRMS (ESI-TOF) *m*/*z*: Calcd. for C_18_H_18_BrNNaO_2_ [M + Na]^+^: 382.0419; Found: 382.0419.


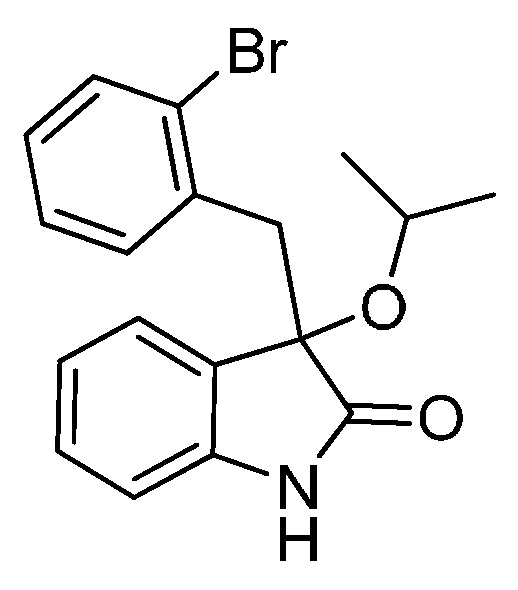


*3-(2-Bromobenzyl)-3-isopropoxyindolin-2-one* (**3ed**). Light orange solid, m.p. 182.0–183.3 °C; yield 74%; ^1^H-NMR (CDCl_3_) δ: 0.99 (d, *J* = 6.0 Hz, 3H), 1.10 (d, *J* = 6.0 Hz, 3H), 3.24 (d, *J* = 14.0 Hz, 1H), 3.40–3.44 (m, 2H), 6.53 (d, *J* = 7.2 Hz, 1H), 6.84–6.89 (m, 2H), 7.03–7.08 (m, 1H), 7.18–7.25 (m, 2H), 7.37–7.41 (m, 1H), 7.55–7.57 (m, 1H), 9.16 (br s, 1H); ^13^C-NMR (CDCl_3_) δ: 23.2, 24.0, 42.7, 69.3, 82.1, 110.5, 122.4, 126.1, 126.7, 126.8, 126.9, 128.5, 129.7, 132.3, 133.1, 134.9, 140.6, 180.4; HRMS (ESI-TOF) *m*/*z*: Calcd. for C_18_H_18_BrNNaO_2_ [M + Na]^+^: 382.0419; Found: 382.0422.


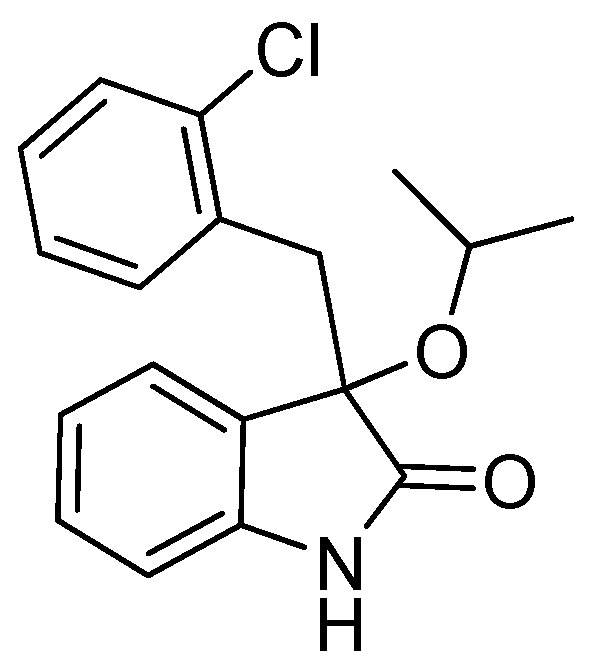


*3-(2-Chlorobenzyl)-3-isopropoxyindolin-2-one* (**3fd**). Light orange solid, m.p. 197.2–198.7 °C; yield 70%; ^1^H-NMR (CDCl_3_) δ: 1.01 (d, *J* = 6.0 Hz, 3H), 1.12 (d, *J* = 6.0 Hz, 3H), 3.27 (d, *J* = 13.6 Hz, 1H), 3.40–3.45 (m, 2H), 6.66 (d, *J* = 7.2 Hz, 1H), 6.87–6.92 (m, 2H), 7.12–7.26 (m, 4H), 7.49–7.52 (m, 1H), 9.16 (br s, 1H); ^13^C-NMR (CDCl_3_) δ: 23.1, 24.0, 40.1, 69.2, 82.1, 110.3, 122.2, 125.9, 126.0, 127.0, 128.1, 128.9, 129.6, 132.9, 135.4, 140.5, 180.1; HRMS (ESI-TOF) *m*/*z*: Calcd. for C_18_H_18_ClNNaO_2_ [M + Na]^+^: 338.0924; Found: 338.0925.


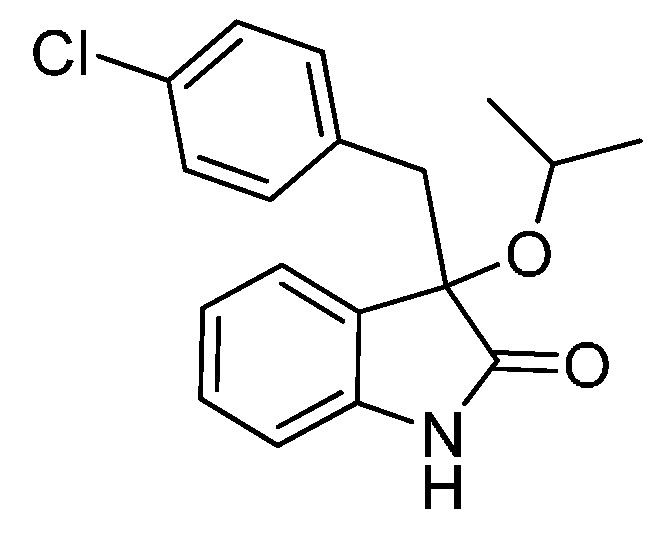


*3-(4-Chlorobenzyl)-3-isopropoxyindolin-2-one* (**3gd**). Light orange solid, m.p. 198.2–201.3 °C; yield 72%; ^1^H-NMR (CDCl_3_) δ: 1.01 (d, *J* = 6.4 Hz, 3H), 1.08 (d, *J* = 6.0 Hz, 3H), 2.98 (d, *J* = 12.8 Hz, 1H), 3.25 (d, *J* = 12.8 Hz, 1H), 3.38–3.45 (m, 1H), 6.81 (d, *J* = 7.6 Hz, 1H), 6.86–6.89 (m, 2H), 6.97–7.04 (m, 4H), 7.22–7.27 (m, 1H), 8.97 (br s, 1H); ^13^C-NMR (CDCl_3_) δ: 23.1, 24.1, 43.7, 69.5, 82.8, 110.4, 122.4, 125.6, 127.3, 127.7, 129.8, 132.0, 132.6, 132.7, 140.6, 179.6; HRMS (ESI-TOF) *m*/*z*: Calcd. for C_18_H_18_ClNNaO_2_ [M + Na]^+^: 338.0924; Found: 338.0926.


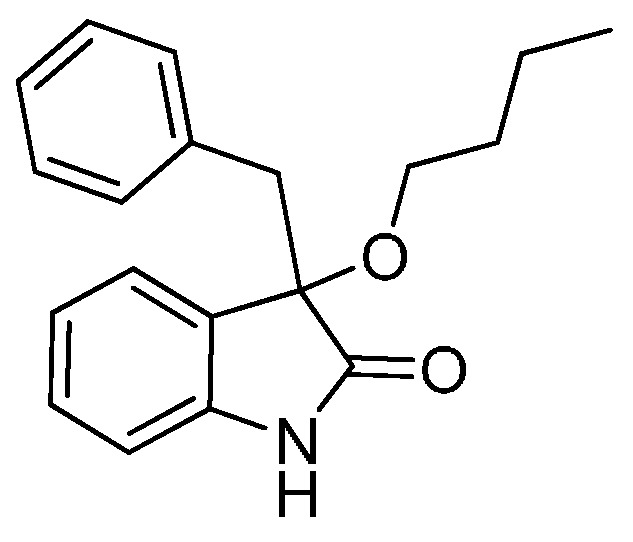


*3-Benzyl-3-butoxyindolin-2-one* (**3ae**). Light orange solid, m.p. 125.5–126.7 °C; yield 80%; ^1^H-NMR (CDCl_3_) δ: 0.82–0.85 (m, 3H), 1.28–1.36 (m, 2H), 1.49–1.54 (m, 2H), 3.01–3.09 (m, 2H), 3.16–3.20 (m, 1H), 3.32 (d, *J* = 12.8 Hz, 1H), 6.78–6.81 (m, 1H), 6.95–7.11 (m, 7H), 7.19–7.26 (m, 1H), 9.01 (br s, 1H); ^13^C-NMR (CDCl_3_) δ: 13.8, 19.1, 31.8, 43.8, 65.3, 83.4, 110.3, 122.4, 125.2, 126.7, 127.2, 127.5, 129.6, 130.6, 134.1, 141.0, 179.1; HRMS (ESI-TOF) *m*/*z*: Calcd. for C_19_H_21_NNaO_2_ [M + Na]^+^: 318.1470; Found: 318.1473.


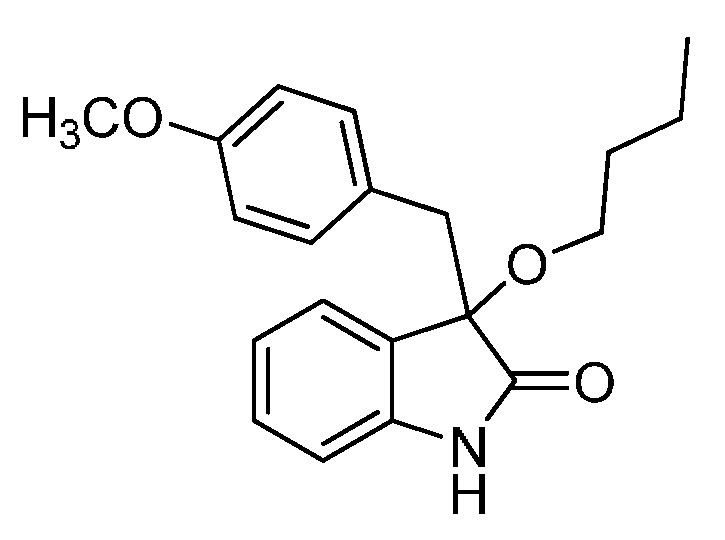


*3-Butoxy-3-(4-methoxybenzyl)indolin-2-one* (**3be**). Light orange solid, m.p. 139.5–141.7 °C; yield 83%; ^1^H-NMR (CDCl_3_) δ: 0.81–0.85 (m, 3H), 1.28–1.34 (m, 2H), 1.48–1.52 (m, 2H), 6.59 (d, *J* = 8.4 Hz, 2H), 6.80 (d, *J* = 7.6 Hz, 1H), 6.87 (d, *J* = 8.8 Hz, 2H), 6.97–7.04 (m, 2H), 7.20–7.24 (m, 1H), 9.08 (br s, 1H); ^13^C-NMR (CDCl_3_) δ: 13.8, 19.1, 31.8, 42.9, 54.9, 65.2, 83.5, 110.3, 112.9, 122.4, 125.2, 126.1, 127.3, 129.5, 131.5, 141.0, 158.2, 179.1; HRMS (ESI-TOF) *m*/*z*: Calcd. for C_20_H_23_NNaO_3_ [M + Na]^+^: 348.1576; Found:348.1576.


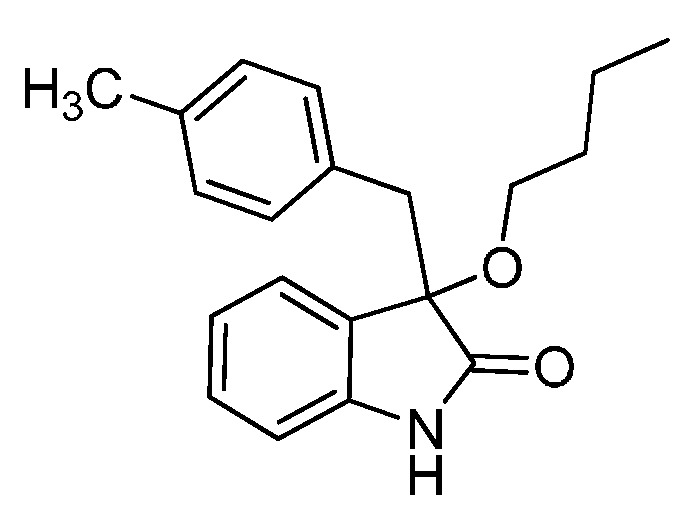


*3-Butoxy-3-(4-methylbenzyl)indolin-2-one* (**3ce**). Light orange solid, m.p. 105.1–107.7 °C; yield 85%; ^1^H-NMR (CDCl_3_) δ: 0.74–0.78 (m, 3H), 1.22–1.25 (m, 2H), 1.43–1.48 (m, 2H), 2.13 (s, 3H), 2.93–2.98 (m, 2H), 3.10–3.13 (m, 1H), 3.20 (d, *J* = 13.2 Hz, 1H), 6.71 (d, *J* = 8.0 Hz, 1H), 6.75–6.81 (m, 4H), 6.90–6.94 (m, 2H), 7.12–7.18 (m, 1H), 8.87 (br s, 1H); ^13^C-NMR (CDCl_3_) δ: 13.8, 19.1, 21.0, 31.9, 43.3, 65.2, 83.4, 110.2, 122.4, 125.2, 127.3, 128.2, 129.5, 130.5, 130.9, 136.1, 141.0, 179.0; HRMS (ESI-TOF) *m*/*z*: Calcd. for C_20_H_23_NNaO_2_ [M + Na]^+^: 332.1626; Found:332.1629.


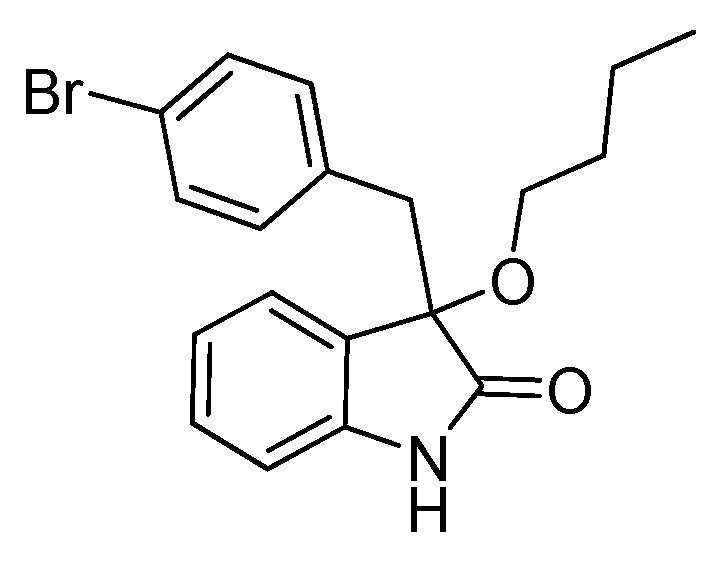


*3-(4-Bromobenzyl)-3-butoxyindolin-2-one* (**3de**). Light orange solid, m.p. 136.0–138.1 °C; yield 84%; ^1^H-NMR (CDCl_3_) δ: 0.82–0.85 (m, 3H), 1.30–1.35 (m, 2H), 1.48–1.54 (m, 2H), 2.99–3.03 (m, 2H), 3.17–3.19 (m, 1H), 3.27 (d, *J* = 13.0 Hz, 1H), 6.80–6.86 (m, 3H), 6.94–6.96 (m, 1H), 7.01–7.04 (m, 1H), 7.20–7.27 (m, 3H), 8.93 (br s, 1H); ^13^C-NMR (CDCl_3_) δ: 13.8, 19.1, 31.8, 43.1, 65.3, 83.0, 110.4, 120.9, 122.6, 125.2, 126.9, 129.8, 130.7, 132.4, 133.2, 140.8, 178.7; HRMS (ESI-TOF) *m*/*z*: Calcd. for C_19_H_20_BrNNaO_2_ [M + Na]^+^: 396.0575; Found: 396.0578.


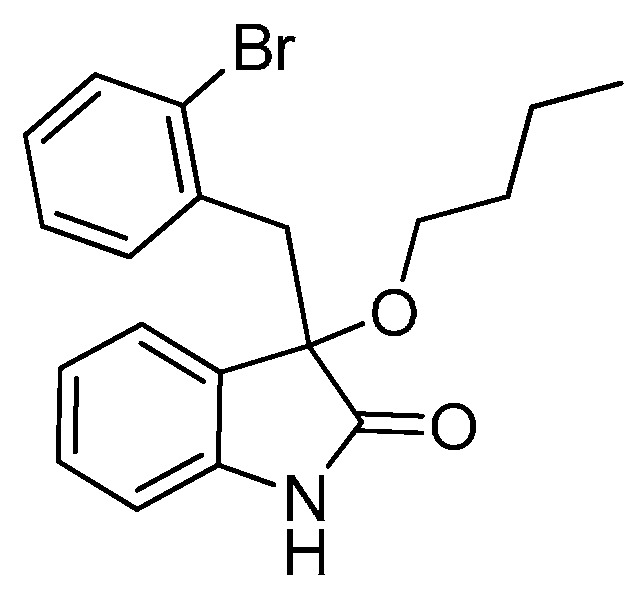


*3-(2-Bromobenzyl)-3-butoxyindolin-2-one* (**3ee**). Light orange solid, m.p. 98.9–99.3 °C; yield 80%; ^1^H-NMR (CDCl_3_) δ: 0.82–0.85 (m, 3H), 1.25–1.36 (m, 2H), 1.45–1.51 (m, 2H), 3.00–3.01 (m, 1H), 3.22–3.24 (m, 1H), 3.32 (d, *J* = 11.2 Hz, 1H), 3.48 (d, *J* = 11.2 Hz, 1H), 6.59 (d, *J* = 6.0 Hz, 1H), 6.89–6.92 (m, 2H), 7.07–7.08 (m, 1H), 7.20–7.25 (m, 2H), 7.40–7.42 (m, 1H), 7.55 (d, *J* = 6.0 Hz, 1H), 9.55 (br s, 1H); ^13^C-NMR (CDCl_3_) δ: 13.8, 19.2, 31.8, 42.1, 65.1, 82.4, 110.4, 122.4, 125.4, 126.5, 126.6, 126.7, 128.4, 129.5, 132.3, 132.9, 134.7, 140.8, 179.4; HRMS (ESI-TOF) *m*/*z*: Calcd. for C_19_H_20_BrNNaO_2_ [M + Na]^+^: 396.0575; Found: 396.0575.


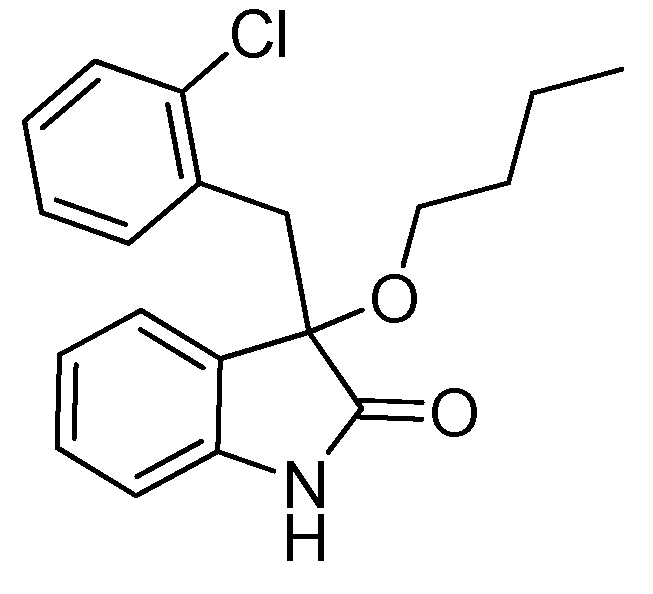


*3-Butoxy-3-(2-chlorobenzyl)indolin-2-one* (**3fe**). Light orange solid, m.p. 142.1–144.0 °C; yield 81%; ^1^H-NMR (CDCl_3_) δ: 0.82–0.86 (m, 3H), 1.33–1.38 (m, 2H), 1.49–1.57 (m, 2H), 2.97–3.03 (m, 1H), 3.20–3.25 (m, 1H), 3.32 (d, *J* = 13.6 Hz, 1H), 3.46 (d, *J* = 13.6 Hz, 1H), 6.69 (d, *J* = 7.6 Hz, 1H), 6.87–6.94 (m, 2H), 7.13–7.26 (m, 4H), 7.47–7.50 (m, 1H), 9.29 (br s, 1H); ^13^C-NMR (CDCl_3_) δ: 13.8, 19.2, 31.8, 39.6, 65.1, 82.5, 110.3, 122.5, 125.4, 126.0, 126.7, 128.2, 129.0, 129.6, 132.8, 132.9, 135.3, 140.7, 179.2; HRMS (ESI-TOF) *m*/*z*: Calcd. for C_19_H_20_ClNNaO_2_ [M + Na]^+^: 352.1080; Found:352.1082.


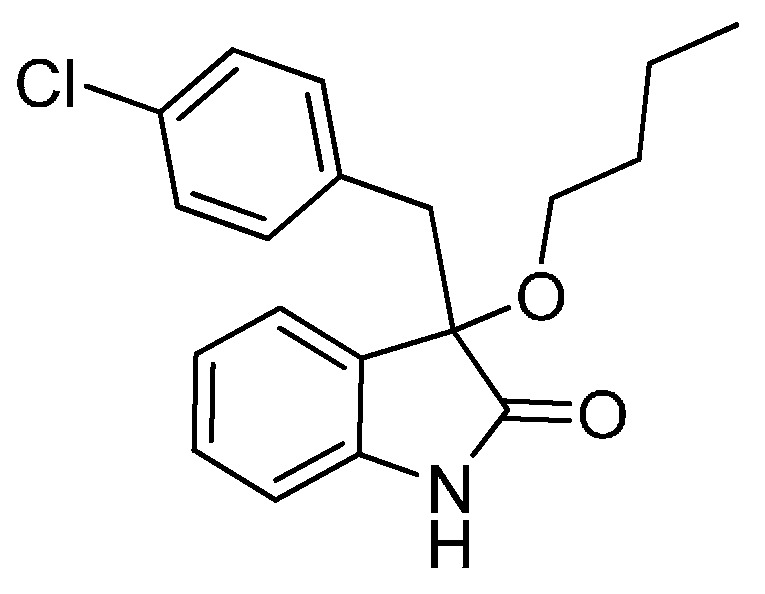


*3-Butoxy-3-(4-chlorobenzyl)indolin-2-one* (**3ge**). Light orange solid, m.p. 142.1–144.3 °C; yield 83%; ^1^H-NMR (CDCl_3_) δ: 0.81–0.85 (m, 3H), 1.30–1.34 (m, 2H), 1.50–1.54 (m, 2H), 3.00–3.05 (m, 2H), 3.16–3.21 (m, 1H), 3.28 (d, *J* = 12.8 Hz, 1H), 6.80–6.83 (m, 1H), 6.89–6.96 (m, 3H), 7.01–7.06 (m, 3H), 7.21–7.27 (m, 1H), 9.09 (br s, 1H); ^13^C-NMR (CDCl_3_) δ: 13.7, 19.1, 31.8, 43.1, 65.3, 83.1, 110.4, 122.6, 125.1, 126.9, 127.7, 129.7, 132.0, 132.7, 140.9, 178.8; HRMS (ESI-TOF) *m*/*z*: Calcd. for C_19_H_20_ClNNaO_2_ [M + Na]^+^: 352.1080; Found:352.1083.


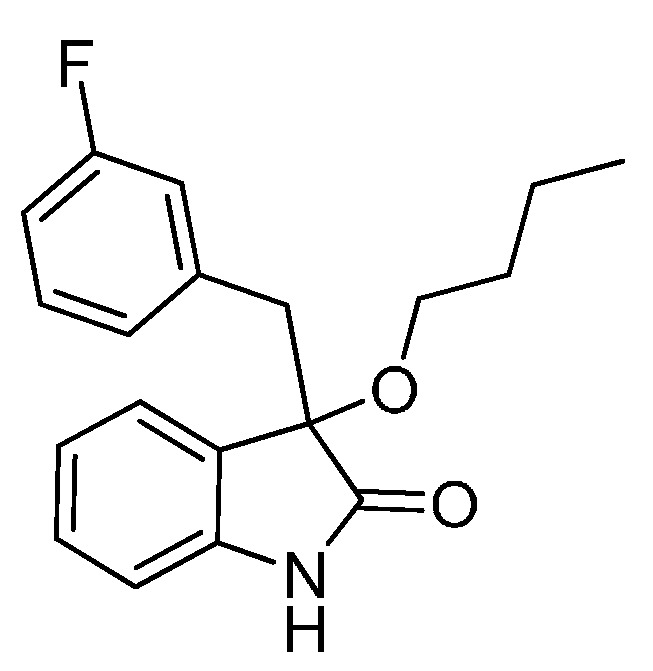


*3-Butoxy-3-(3-fluorobenzyl)indolin-2-one* (**3he**). Light orange solid, m.p. 129.0–131.1 °C; yield 74%; ^1^H-NMR (CDCl_3_, 500 MHz) δ: 0.82–0.86 (m, 3H), 1.26–1.35 (m, 2H), 1.45–1.57 (m, 2H), 3.02–3.05 (m, 2H), 3.19–3.22 (m, 1H), 3.32 (d, *J* = 13.5 Hz, 1H), 6.72–6.76 (m, 2H), 6.80–6.84 (m, 2H), 6.91–6.95 (m, 1H), 7.01–7.05 (m, 2H), 7.22–7.26 (m, 1H), 9.18 (br s, 1H); ^13^C-NMR (CDCl_3_, 125 MHz) δ: 13.7, 19.1, 31.8, 43.4, 65.3, 83.0, 110.4, 113.6 (d, *J_CF_* = 21.1 Hz), 117.4 (d, *J_CF_* = 21.2 Hz), 122.6, 125.1, 126.4, 126.9, 128.8, 128.9, 129.8, 136.7, 136.8, 140.9, 162.1 (d, *J_CF_* = 243.8 Hz), 178.9; HRMS (ESI-TOF) *m*/*z*: Calcd. for C_19_H_20_FNNaO_2_ [M + Na]^+^: 336.1376; Found:336.1377.


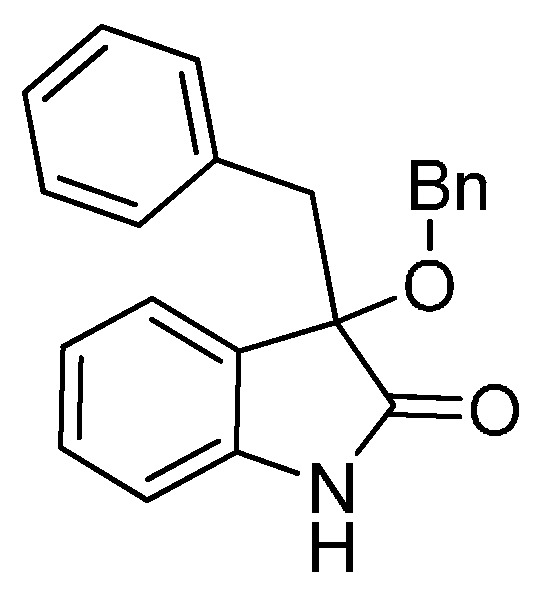


*3-Benzyl-3-(benzyloxy)indolin-2-one* (**3af**). Light orange solid, m.p. 168.8–170.3 °C; yield 84%; ^1^H-NMR (CDCl_3_) δ: 3.18 (d, *J* = 12.8 Hz, 1H), 3.41 (d, *J* = 12.8 Hz, 1H), 4.12 (d, *J* = 8.2 Hz, 1H), 4.25 (d, *J* = 12.8 Hz, 1H), 6.80 (d, J = 8.0 Hz, 1H), 6.97–7.10 (m, 7H), 7.21–7.29 (m, 6H), 8.97 (br s, 1H); ^13^C-NMR (CDCl_3_) δ: 43.8, 67.8, 83.8, 110.4, 122.6, 125.4, 126.8, 127.6, 127.7, 127.8, 128.2, 129.9, 130.7, 133.9, 137.5, 141.1, 178.4; HRMS (ESI-TOF) *m*/*z*: Calcd. for C_22_H_19_NNaO_2_ [M + Na]^+^: 352.1313; Found:352.1313.


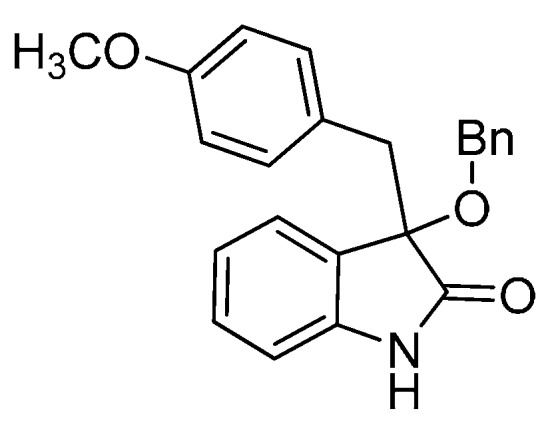


*3-(Benzyloxy)-3-(4-methoxybenzyl)indolin-2-one* (**3bf**). Light orange solid, m.p. 218.1–219.8 °C; yield 85%; ^1^H-NMR (CDCl_3_) δ: 3.12 (d, *J* = 13.2 Hz, 1H), 3.34 (d, *J* = 13.2 Hz, 1H), 3.64 (s, 3H), 4.11 (d, *J* = 10.8 Hz, 1H), 4.23 (d, *J* = 10.4 Hz, 1H), 6.58–6.61 (m, 2H), 6.79–6.81 (m, 1H), 6.87–6.89 (m, 2H), 7.05–7.10 (m, 2H), 7.22–7.29 (m, 6H), 8.88 (br s, 1H); ^13^C-NMR (CDCl_3_) δ: 43.0, 55.1, 68.0, 84.0, 110.6, 113.2, 122.8, 125.5, 126.0, 127.1, 127.8, 128.0, 128.3, 130.0, 131.8, 137.7, 141.2, 158.5, 178.7; HRMS (ESI-TOF) *m*/*z*: Calcd. for C_23_H_21_NNaO_3_ [M + Na]^+^: 382.1419; Found:382.1421.


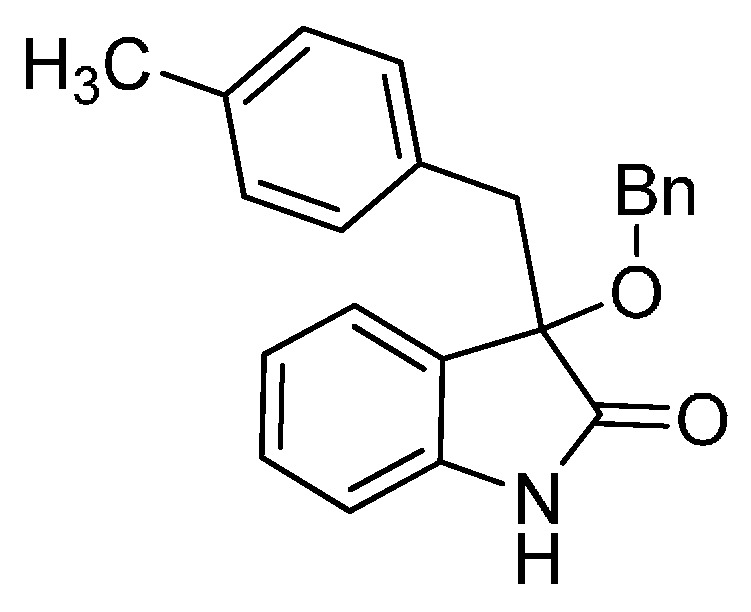


*3-(Benzyloxy)-3-(4-methylbenzyl)indolin-2-one* (**3cf**). Light orange solid, m.p. 132.2–134.3 °C; yield 80%; ^1^H-NMR (CDCl_3_) δ: 2.10 (s, 3H), 3.06 (d, *J* = 13.2 Hz, 1H), 3.28 (d, *J* = 12.8 Hz, 1H), 4.03 (d, *J* = 10.4 Hz, 1H), 4.16 (d, *J* = 10.8 Hz, 1H), 6.72 (d, *J* = 8.0 Hz, 1H), 6.78 (s, 4H), 6.96–7.02 (m, 2H), 7.14–7.20 (m, 6H), 8.93 (br s, 1H); ^13^C-NMR (CDCl_3_) δ: 21.0, 43.3, 67.8, 83.8, 110.4, 122.6, 125.3, 126.9, 127.6, 127.8, 128.2, 128.4, 129.8, 130.5, 130.7, 136.2, 137.6, 141.1, 178.5; HRMS (ESI-TOF) *m*/*z*: Calcd. for C_23_H_21_NNaO_2_ [M + Na]^+^: 366.1470; Found:366.1474.


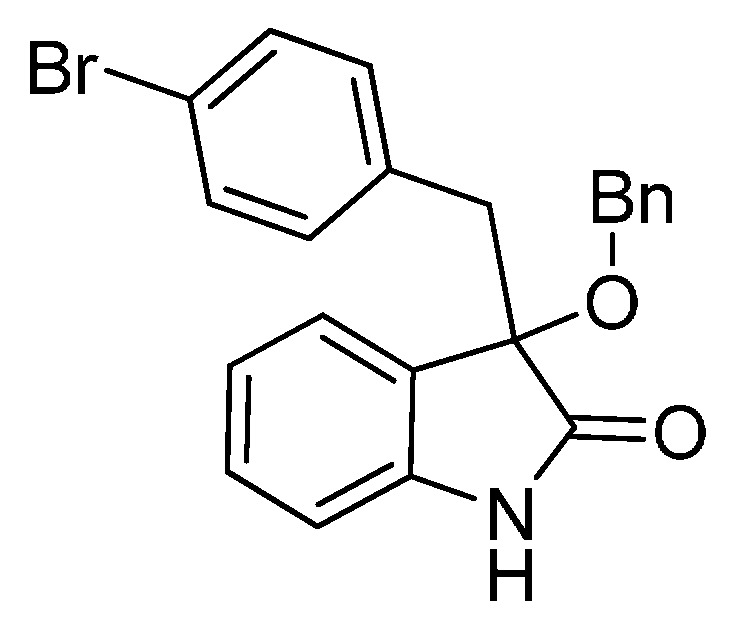


*3-(Benzyloxy)-3-(4-bromobenzyl)indolin-2-one* (**3df**). Light orange solid, m.p. 201.2–203.2 °C; yield 82%; ^1^H-NMR (DMSO-*d*_6_) δ: 3.08 (d, *J* = 10.0 Hz, 1H), 3.27 (d, *J* = 10.0 Hz, 1H), 3.99 (d, *J* = 8.4 Hz, 1H), 4.12 (d, *J* = 8.4 Hz, 1H), 6.71 (d, *J* = 6.0 Hz, 1H), 6.86 (d, *J* = 6.4 Hz, 1H), 7.19–7.22 (m, 1H), 7.26–7.31 (m, 9H); ^13^C-NMR (DMSO-*d*_6_) δ: 41.4, 66.1, 82.3, 109.5, 119.5, 121.4, 124.5, 125.6, 127.0, 127.1, 127.7, 129.5, 130.0, 132.0, 133.2, 137.2, 141.8, 175.6; HRMS (ESI-TOF) *m*/*z*: Calcd. for C_22_H_18_BrNNaO_2_ [M + Na]^+^: 430.0419; Found:430.0423.


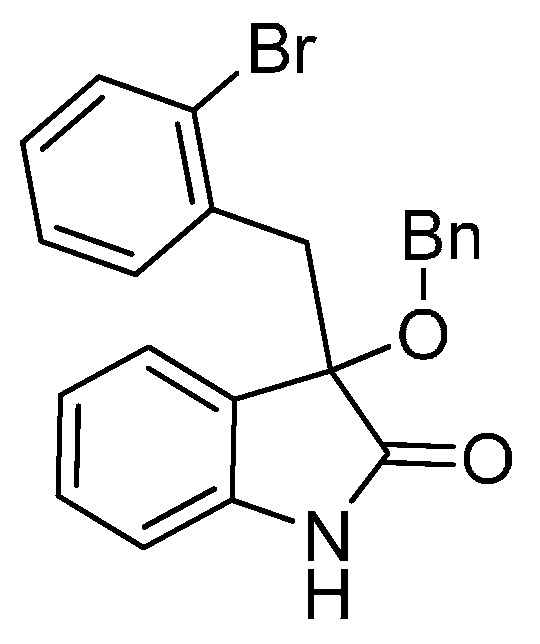


*3-(Benzyloxy)-3-(2-bromobenzyl)indolin-2-one* (**3ef**). Light orange solid, m.p. 158.8–160.1 °C; yield 81%; ^1^H-NMR (CDCl_3_) δ: 3.42 (d, *J* = 14.0 Hz, 1H), 3.58 (d, *J* = 14.0 Hz, 1H), 4.12 (d, *J* = 10.8 Hz, 1H), 4.30 (d, *J* = 10.8 Hz, 1H), 6.70 (d, *J* = 7.6 Hz, 1H), 6.91–6.95 (m, 2H), 7.07–7.09 (m, 1H), 7.21–7.30 (m, 7H), 7.42 (d, *J* = 8.1 Hz, 1H), 7.56 (d, *J* = 8.0 Hz, 1H), 9.35 (br s, 1H); ^13^C-NMR (CDCl_3_) δ: 42.2, 67.8, 82.9, 110.7, 122.8, 125.8, 126.3, 126.6, 127.0, 127.7, 127.8, 128.3, 128.7, 130.0, 132.5, 133.1, 134.6, 137.8, 141.0, 179.0; HRMS (ESI-TOF) *m*/*z*: Calcd. for C_22_H_18_BrNNaO_2_ [M + Na]^+^: 430.0419; Found:430.0418.


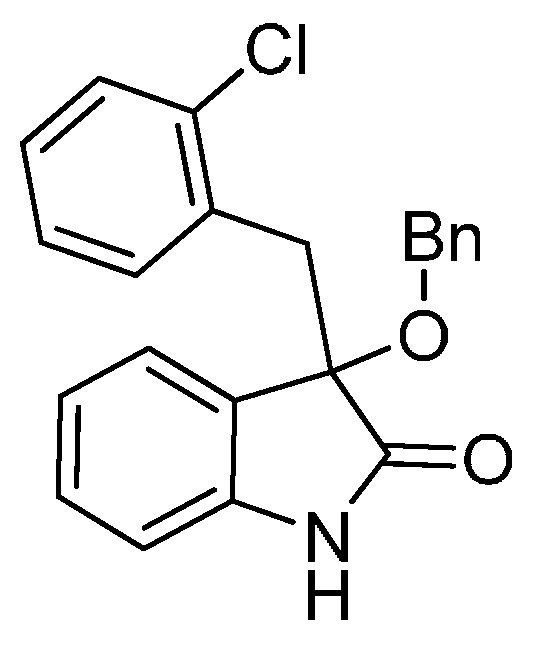


*3-(Benzyloxy)-3-(2-chlorobenzyl)indolin-2-one* (**3ff**). Light orange solid, m.p. 145.3–147.2 °C; yield 82%; ^1^H-NMR (CDCl_3_) δ: 3.42 (d, *J* = 14.0 Hz, 1H), 3.56 (d, *J* = 13.6 Hz, 1H), 4.12 (d, *J* = 10.8 Hz, 1H), 4.29 (d, *J* = 10.8 Hz, 1H), 6.80 (d, *J* = 7.2 Hz, 1H), 6.89–6.96 (m, 2H), 7.12–7.15 (m, 2H), 7.20–7.28 (m, 7H), 7.48–7.50 (m, 1H), 9.34 (br s, 1H); ^13^C-NMR (CDCl_3_) δ: 39.6, 67.7, 82.9, 110.5, 122.6, 125.5, 126.1, 126.3, 127.6, 128.2, 128.3, 129.1, 129.9, 132.6, 132.9, 135.3, 137.6, 140.8, 178.7; HRMS (ESI-TOF) *m*/*z*: Calcd. for C_22_H_18_ClNNaO_2_ [M + Na]^+^: 386.0924; Found:386.0925.


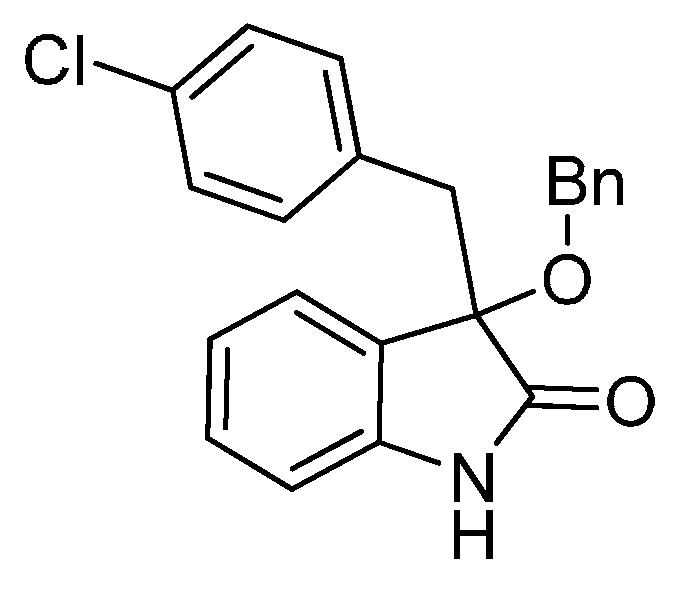


*3-(Benzyloxy)-3-(4-chlorobenzyl)indolin-2-one* (**3gf**). Light orange solid, m.p. 110.3–112.8 °C; yield 83%; ^1^H-NMR (DMSO–*d*_6_) δ: 3.09 (d, *J* = 12.8 Hz, 1H), 3.29 (d, *J* = 12.8 Hz, 1H), 3.99 (d, *J* = 10.8 Hz, 1H), 4.13 (d, *J* = 10.8 Hz, 1H), 6.71 (d, *J* = 8.0 Hz, 1H), 6.92 (d, *J* = 8.4 Hz, 2H), 7.00–7.03 (m, 1H), 7.15–7.30 (m, 9H), 10.5 (br s, 1H); ^13^C-NMR (DMSO-*d*_6_) δ: 41.4, 66.1, 82.4, 109.5, 121.4, 124.5, 125.7, 127.0, 127.1, 127.7, 129.5, 131.0, 131.6, 132.9, 137.2, 141.8, 175.6; HRMS (ESI-TOF) *m*/*z*: Calcd. for C_22_H_18_ClNNaO_2_ [M + Na]^+^: 386.0924; Found:386.0924.


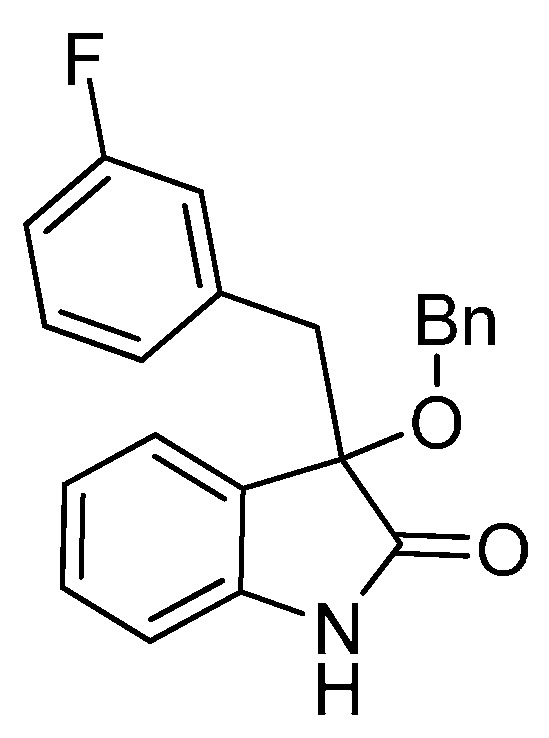


*3-(Benzyloxy)-3-(3-fluorobenzyl)indolin-2-one* (**3hf**). Light orange solid, m.p. 153.2–155.1 °C; yield 75%; ^1^H-NMR (CDCl_3_, 500 MHz) δ: 3.16 (d, *J* = 10.4 Hz, 1H), 3.39 (d, *J* = 13.4 Hz, 1H), 4.13 (d, *J* = 11.0 Hz, 1H), 4.27 (d, *J* = 10.5 Hz, 1H), 6.72–6.74 (m, 1H), 6.76–6.78 (m, 1H), 6.81–6.84 (m, 2H), 7.04–7.06 (m, 3H), 7.23–7.29 (m, 6H); ^13^C-NMR (CDCl_3_, 125 MHz) δ: 43.4, 67.9, 83.4, 110.5, 113.7 (d, *J_CF_* = 20.1 Hz), 117.4 (d, *J_CF_* = 21.3 Hz), 122.8, 125.3, 126.5, 127.7, 127.8, 128.3, 129.0, 129.1, 130.1, 136.5, 137.4, 141.0, 162.1 (d, *J_CF_* = 243.8 Hz), 178.2; HRMS (ESI-TOF) *m*/*z*: Calcd. for C_22_H_18_FNNaO_2_ [M + Na]^+^: 370.1219; Found:370.1221.


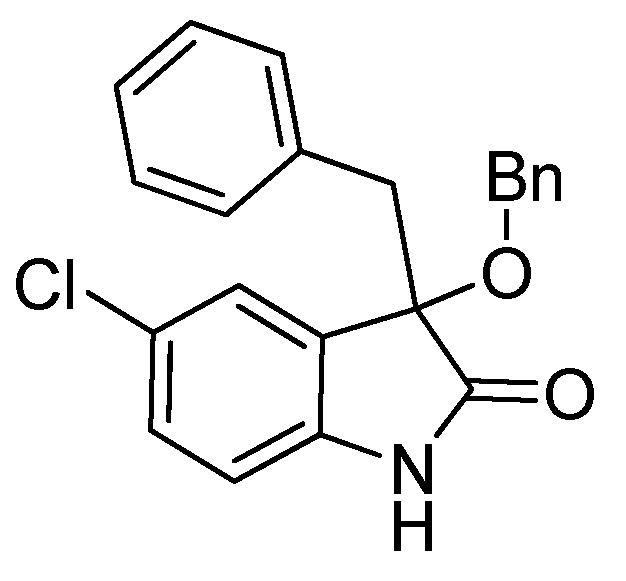


*3-Benzyl-3-(benzyloxy)-5-chloroindolin-2-one* (**3if**). Light orange solid, m.p. 217.3–220.5 °C; yield 82%; ^1^H-NMR (DMSO–*d*_6_) δ: 3.09 (d, *J* = 12.4 H, 1H), 3.33 (d, *J* = 12.8 Hz, 1H), 4.03 (d, *J* = 10.8 Hz, 1H), 4.16 (d, *J* = 10.8 Hz, 1H), 6.68 (d, *J* = 8.8 Hz, 1H), 6.92–6.95 (m, 2H), 7.11–7.13 (m, 3H), 7.22–7.32 (m, 7H), 10.6 (br s, 1H); ^13^C-NMR (DMSO-*d*_6_) δ: 42.4, 66.8, 83.2, 111.3, 125.2, 125.9, 126.8, 127.5, 127.6, 127.7, 128.2, 128.5, 129.7, 130.3, 133.9, 137.6, 141.2, 176.0; HRMS (ESI-TOF) *m*/*z*: Calcd. for C_22_H_18_ClNNaO_2_ [M + Na]^+^: 386.0924; Found:386.0926.


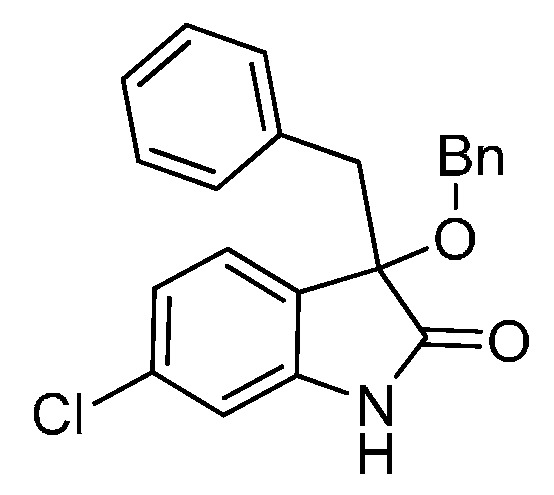


*3-Benzyl-3-(benzyloxy)-6-chloroindolin-2-one* (**3jf**). Light orange solid, m.p. 165.1–168.5 °C; yield 85%; ^1^H-NMR (CDCl_3_) δ: 3.04 (d, *J* = 13.2 Hz, 1H), 3.31 (d, *J* = 13.2 Hz, 1H), 4.01 (d, *J* = 10.8 Hz, 1H), 4.16 (d, *J* = 10.8 Hz, 1H), 6.75–6.76 (m, 1H), 6.84–6.94 (m, 4H), 7.00–7.06 (m, 3H), 7.16–7.23 (m, 5H), 9.04 (br s, 1H); ^13^C-NMR (CDCl_3_) δ: 43.6, 67.9, 83.4, 111.2, 122.7, 125.1, 126.4, 127.0, 127.8, 128.3, 130.7, 133.6, 135.5, 137.2, 142.1, 178.5; HRMS (ESI-TOF) *m*/*z*: Calcd. for C_22_H_18_ClNNaO_2_ [M + Na]^+^: 386.0924; Found: 386.0925.


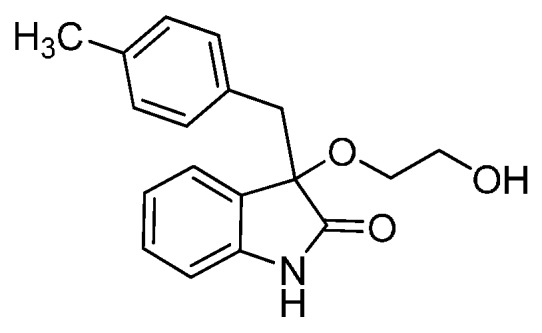


*3-(2-Hydroxyethoxy)-3-(4-methylbenzyl)indolin-2-one* (**3cg**). Light orange solid, m.p. 211.6–213.4 °C; yield 83%; ^1^H-NMR (DMSO-*d*_6_) δ: 2.14 (s, 3H), 2.95–3.03 (m, 2H), 3.08–3.12 (m, 1H), 3.17 (d, *J* = 10.0 Hz, 1H), 3.38–3.42 (m, 2H), 4.57 (br s, 1H), 6.61 (d, *J* = 6.4 Hz, 1H), 6.73 (d, *J* = 6.4 Hz, 2H), 6.86 (d, *J* = 6.0 Hz, 2H), 6.96–6.99 (m, 1H), 7.14–7.17 (m, 1H), 7.21 (d, *J* = 5.6 Hz, 1H), 10.3 (br s, 1H); ^13^C-NMR (DMSO-*d*_6_) δ: 20.1, 41.7, 59.6, 65.9, 82.5, 109.2, 121.2, 124.5, 126.3, 127.7, 129.2, 129.6, 130.6, 135.0, 141.8, 175.8; HRMS (ESI-TOF) *m*/*z*: Calcd. for C_18_H_19_NNaO_3_ [M + Na]^+^: 320.1263; Found:320.1267.


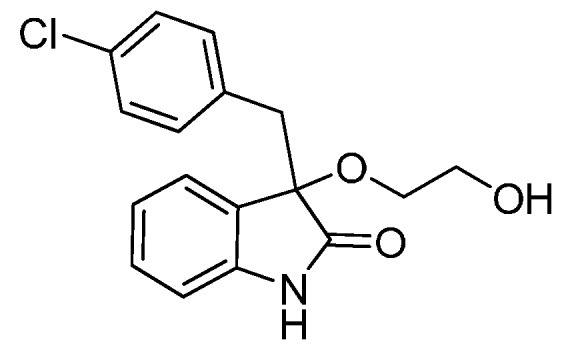


*3-(4-Chlorobenzyl)-3-(2-hydroxyethoxy)indolin-2-one* (**3gg**). Light orange solid, m.p. 205.3–207.3 °C; yield 87%; ^1^H-NMR (CDCl_3_) δ: 2.93–2.97 (m, 1H), 3.03 (d, *J* = 6.4 Hz, 1H), 3.07–3.11 (m, 1H), 3.21 (d, *J* = 6.4 Hz, 1H), 3.39–3.43 (m, 2H), 4.56–4.58 (m, 1H), 6.64–6.65 (m, 1H), 6.88 (d, *J* = 6.4 Hz, 2H), 6.97–7.00 (m, 1H), 7.13–7.19 (m, 4H), 10.4 (br s, 1H); ^13^C-NMR (CDCl_3_) δ: 41.3, 59.5, 66.0, 82.2, 109.3, 121.3, 124.5, 125.9, 127.1, 129.4, 130.9, 131.5, 132.9, 141.7, 175.7; HRMS (ESI-TOF) *m*/*z*: Calcd. for C_17_H_16_ClNNaO_3_ [M + Na]^+^: 340.0716; Found:340.0716.


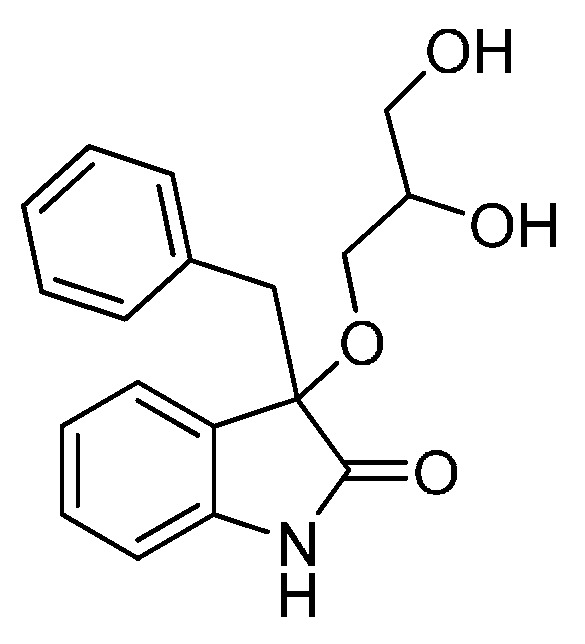


*3-Benzyl-3-(2,3-dihydroxypropoxy)indolin-2-one* *(***3ah**). Light orange oil; yield 51%, 1:1*dr*; ^1^H-NMR (CDCl_3_) δ: 3.03–3.10 (m, 2H), 3.12–3.18 (m, 1H), 3.21–3.32 (m, 2.6 H), 3.45–3.49 (m, 1H), 3.59–3.84 (m, 2.6 H), 6.74–6.77 (m, 1H), 6.92–7.04 (m, 4H), 7.07–7.15 (m, 3H), 7.19–7.23 (m, 1H), 8.65 (br s, 1H); ^13^C -NMR (CDCl_3_) δ: 43.6, 63.4, 63.5, 66.7, 67.7, 70.4, 70.7, 83.6, 83.7, 110.6, 110.7, 122.8, 126.9, 127.7, 130.0, 130.5, 130.6, 133.6, 140.8, 178.7, 178.8; HRMS (ESI-TOF) *m*/*z*: Calcd. for C_18_H_19_NNaO_4_ [M + Na]^+^: 336.1212; Found:336.1215.

## 4. Conclusions

In conclusion, we have developed a highly efficient and eco-friendly method for the synthesis of multisubstituted 3-alkoxylated-2-oxindoles **3** via direct alkoxylation of 3-halooxindoles **1**. A wide variety of multisubstituted 3-alkoxylated-2-oxindole scaffolds were obtained smoothly in good yields (up to 94%) by simple heating in an oil bath at 30 °C for 24 h. A particularly valuable feature of this method was the development of environmentally-friendly chemistry using alcohols **2** as both the substrates and solvents. Moreover, only a catalytic amount of Na_2_CO_3_, a very cheap buck chemical, was used as the catalyst in this transformation, which made this chemistry exceptionally appealing for practical application. 

## Figures and Tables

**Table 1:** Optimization of reaction conditions *^a^*.

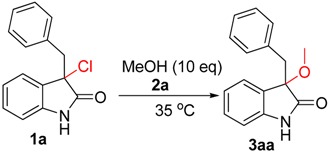				
Entry *^a^*	Solvent	Catalyst (20 mol %)	Time (h)	Yield *^b^* (%)
1 *^c^*	MeOH	DABCO	24	trace
2 *^c^*	MeOH	DBU	24	54
3 *^c^*	MeOH	Et_3_N	24	73
4 *^c^*	MeOH	K_2_CO_3_	24	87
5 *^c^*	MeOH	NaHCO_3_	24	76
6 *^c^*	MeOH	Na_2_CO_3_	24	94
7	MeOH	None	24	<10
8	toluene	Na_2_CO_3_	24	61
9	DCM	Na_2_CO_3_	24	59
10	EtOAc	Na_2_CO_3_	24	10
11	THF	Na_2_CO_3_	24	13
12 *^c^*	CH_3_CN	Na_2_CO_3_	24	51
13 *^c^*	MeOH	Na_2_CO_3_	4	63
14 *^c^*	MeOH	Na_2_CO_3_	6	79
15 *^c,d^*	MeOH	Na_2_CO_3_	15	96
16 *^c,e^*	MeOH	Na_2_CO_3_	48	78
17 *^c,f^*	MeOH	Na_2_CO_3_	72	61

*^a^* Unless otherwise noted, reactions were carried out with 0.4 mmol of **1a**, 2.0 mmol of **2a**, 20 mol % of catalyst in the 4.0 mL of solvent for the specified time. *^b^* Isolated yield after flash chromatography. *^c^* The reaction was carried out using MeOH as substrate and solvent. *^d^* The reaction was carried out using 100 mol % of base Na_2_CO_3_. *^e^* The reaction was carried out using 10 mol % of base Na_2_CO_3_. *^f^* The reaction was carried out using 5 mol % of base Na_2_CO_3_.

**Table 2:** Synthesis of 3-alkoxylated-2-oxindoles **3** *^a,b^*.

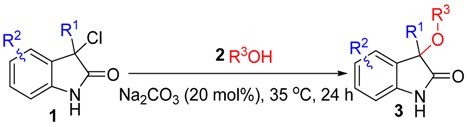
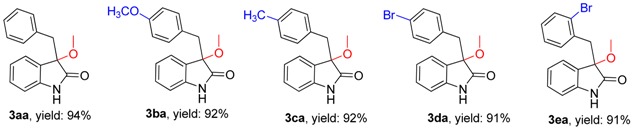
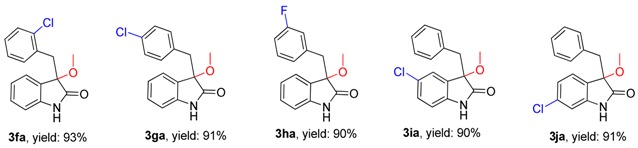
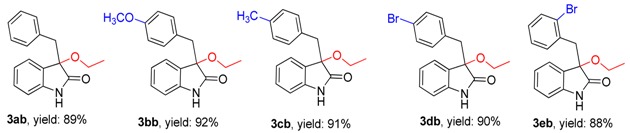
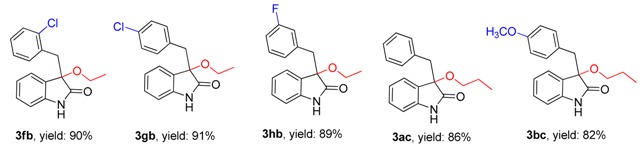
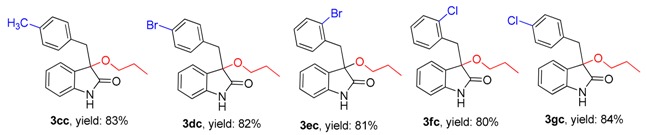
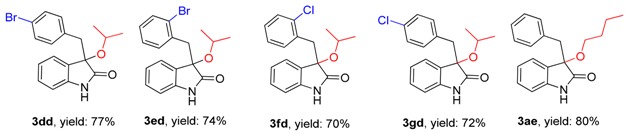
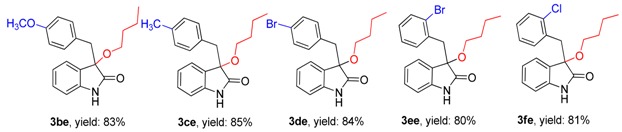
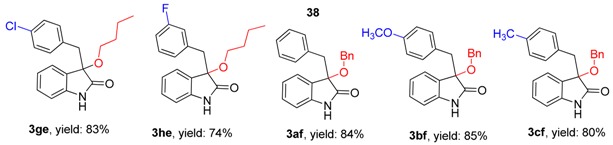
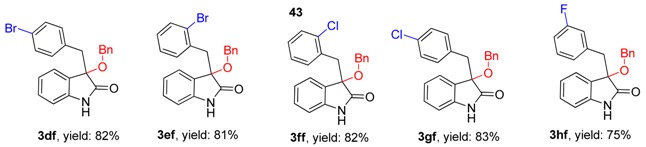
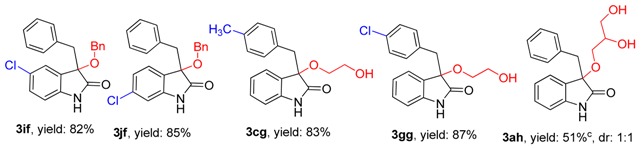

*^a^* Unless otherwise noted, reactions were carried out with 0.4 mmol of **1** and 20 mol % of catalyst Na_2_CO_3_ in the 4.0 mL of alcohol **2** in oil bath at 35 °C for 24 h. *^b^* Isolated yield after flash chromatography. *^c^* This reaction was carried out in oil bath at 45 °C for 24 h.

## Supplementary Materials

Supplementary materials are available online.
