# Supplementary material for: Alcohols as Substrates and Solvents for the Construction of 3-Alkoxylated-2-Oxindoles by Direct Alkoxylation of 3-Halooxindoles
Source: Molecules. 2017 May 13;22(5):801. doi: 10.3390/molecules22050801 (PMC6154289; doi:10.3390/molecules22050801)

Alcohols as Substrates and Solvents for Construction of 3-Alkoxylated-2-Oxindoles by Direct Alkoxylation of 3-Halooxindoles

Bing Lin,[1,3] Zhi-Yong Chen,[1,3] Huan-Huan Liu,[1] Qi-Di Wei,[1] Ting-Ting Feng,[1] Ying Zhou,*[1] Can Wang,[1] Xiong-Li Liu*[1] and Wei-Cheng Yuan[2]

[1] Guizhou Medicine Edicine Edible Plant Resources Research and Development Center, College of Pharmacy, Guizhou University, Guiyang, 550025; nlin@gzu.edu.cn (B.L.)

[2] Key Laboratory for Asymmetric Synthesis & Chirotechnology of Sichuan Province, Chengdu Institute of Organic Chemistry, Chinese Academy of Sciences, Chengdu 610041, China; yuanwc@cioc.ac.cn

[3] These two authors contributed equally to this work.

* Correspondence: yzhou71@yeah.net (Y.Z.); xlliu1@gzu.edu.cn (X.L.L.)

Supporting Information

Table of Contents

1. The Copies of ^{1}H NMR, ^{13}C NMR Spectra for Compounds **3**…………..……………………S2

1. The Copies of ^{1}H NMR, ^{13}C NMR Spectra for Compounds 3.

^{1}H and ^{13}C NMR of 3aa

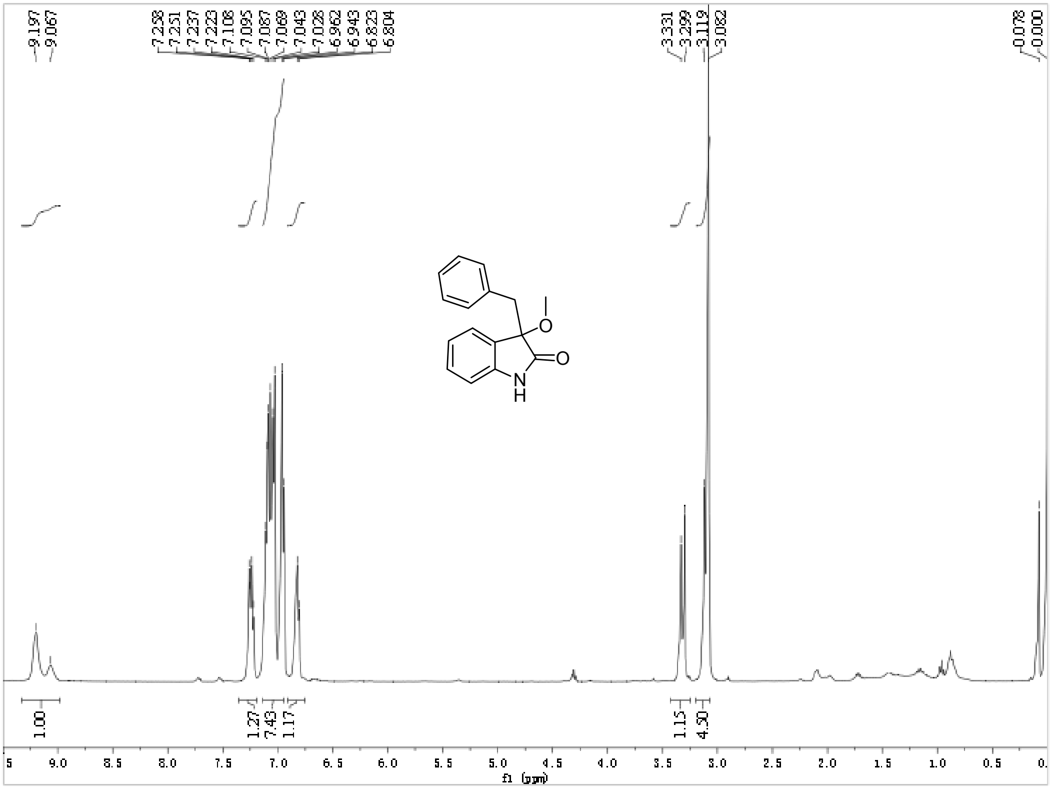

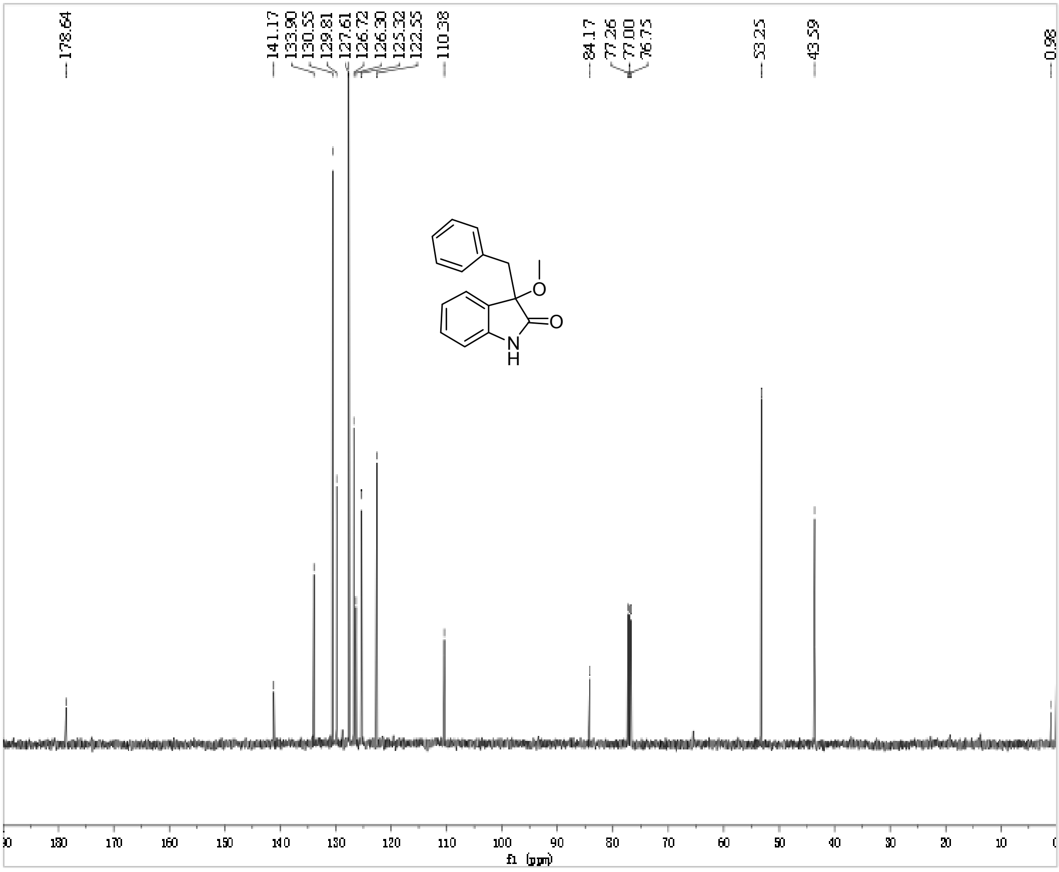

^{1}H and ^{13}C NMR of 3ba

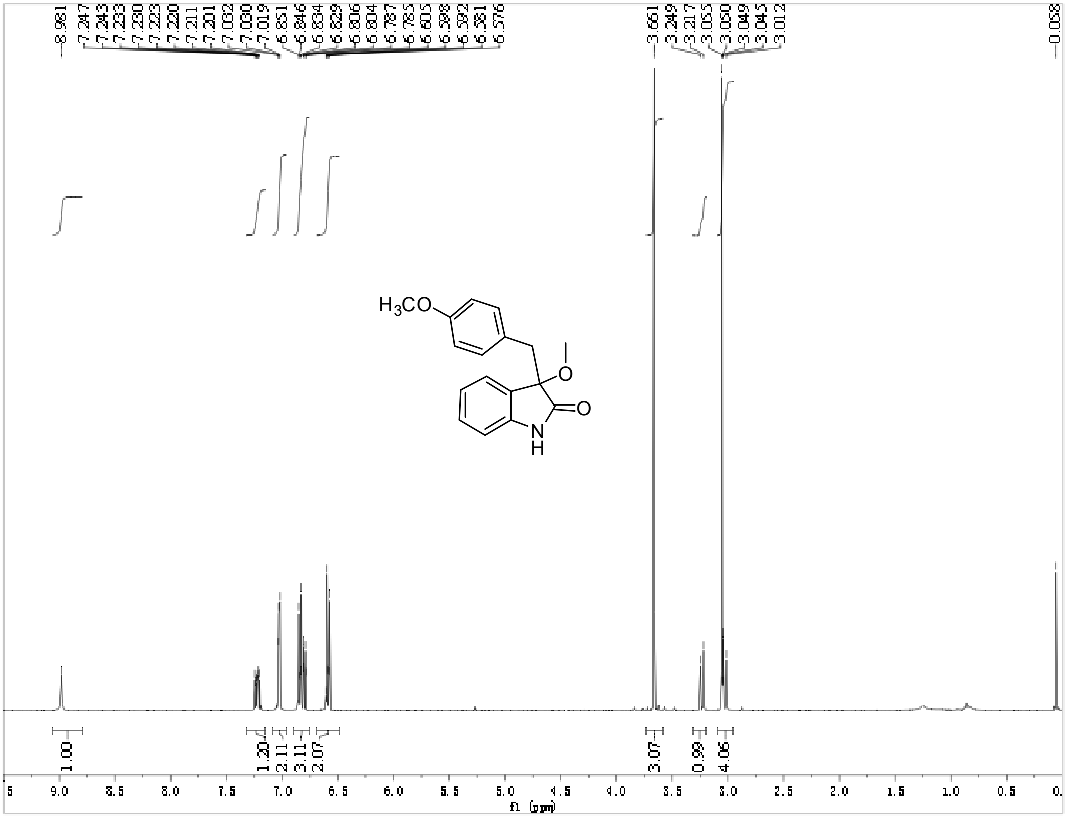

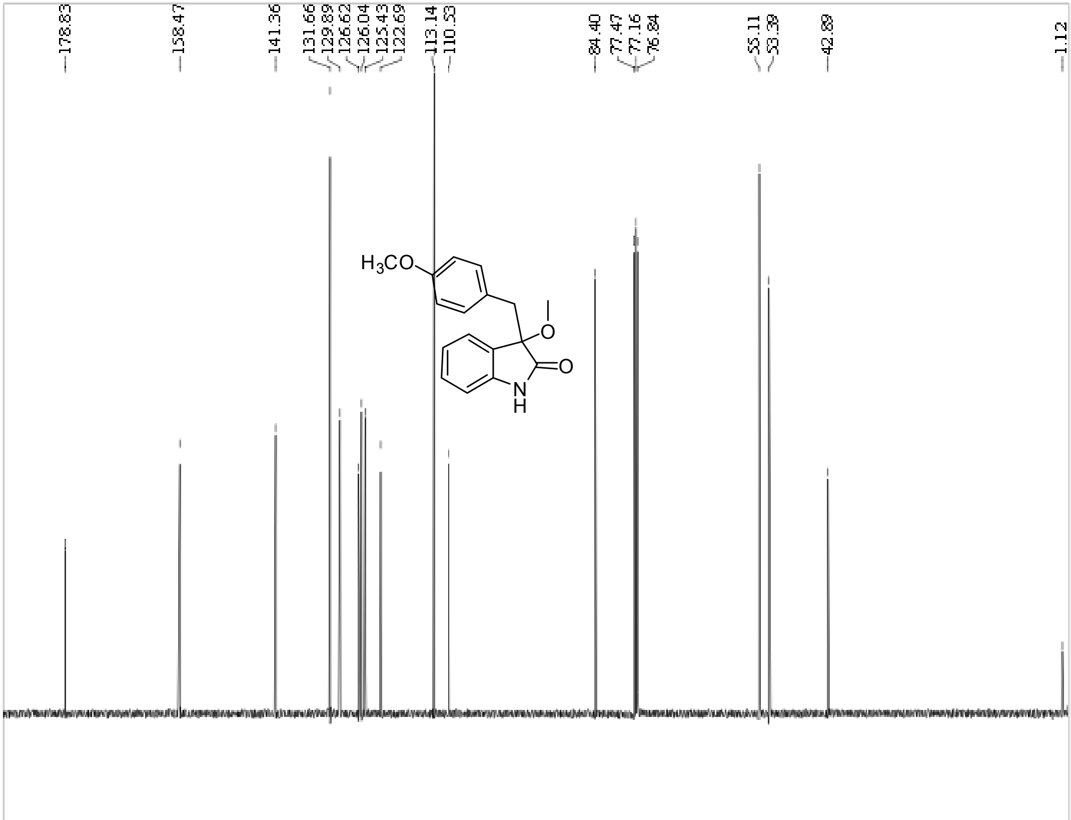

^{1}H and ^{13}C NMR of 3ca

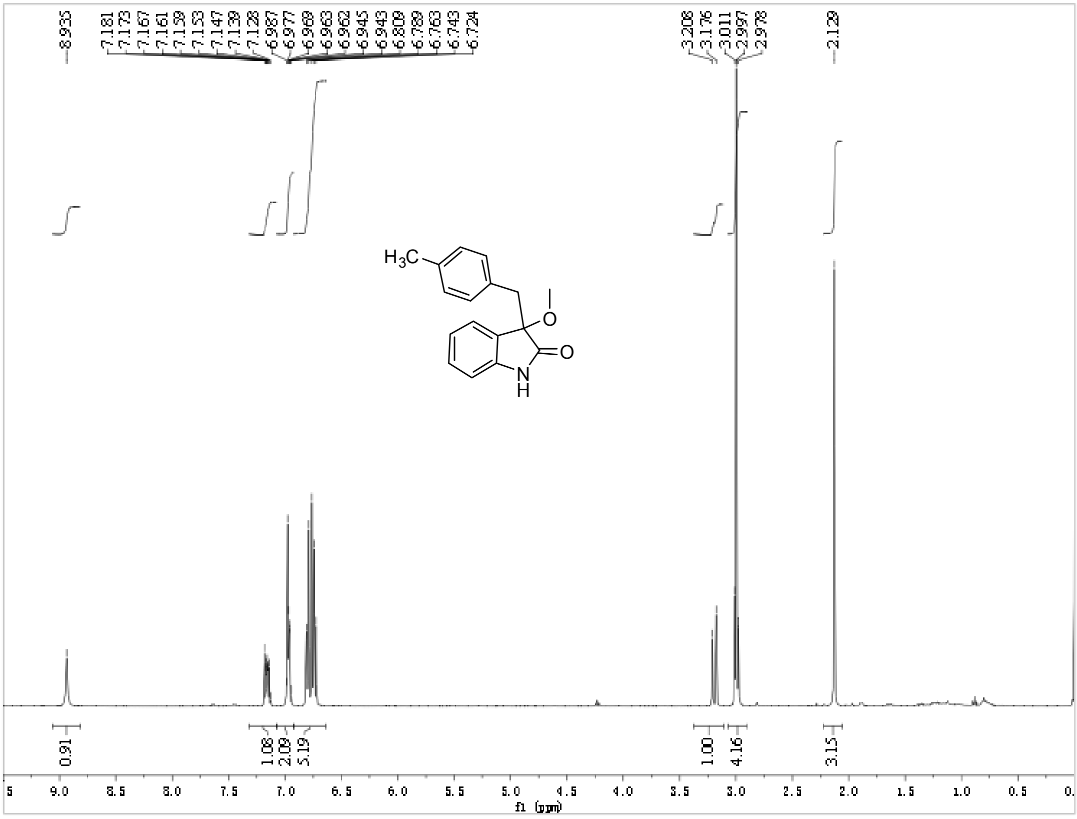

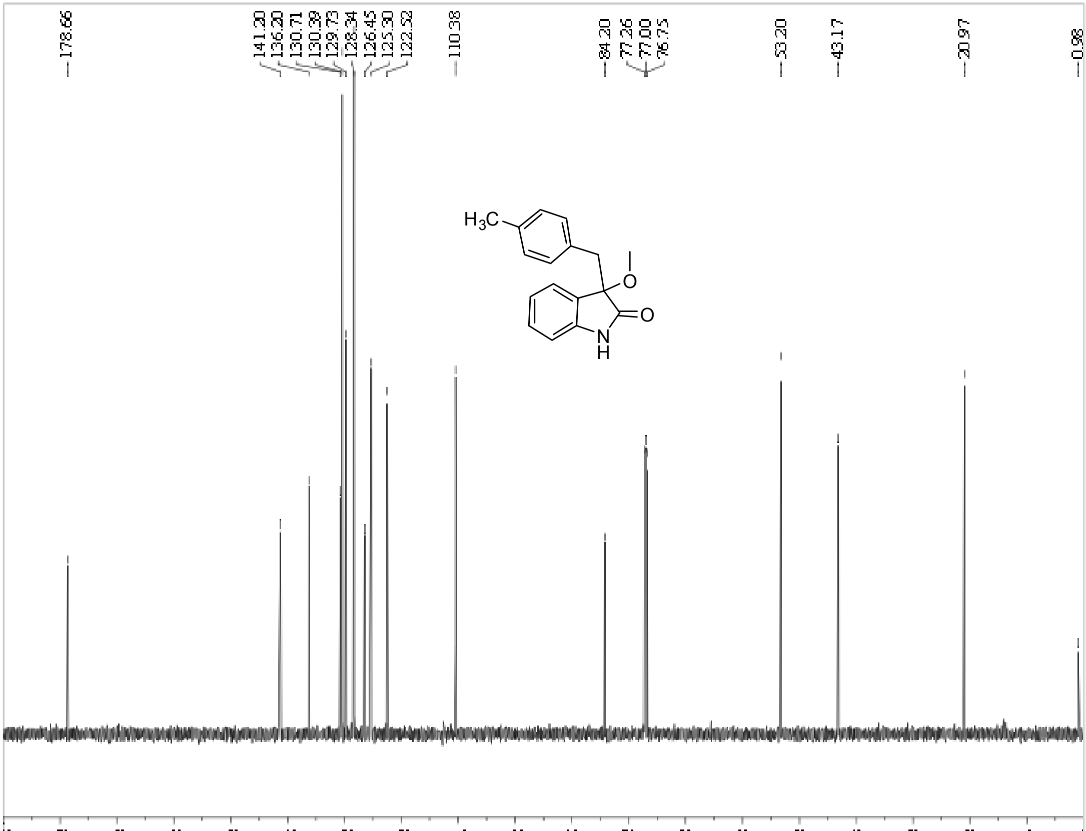

^{1}H and ^{13}C NMR of 3da

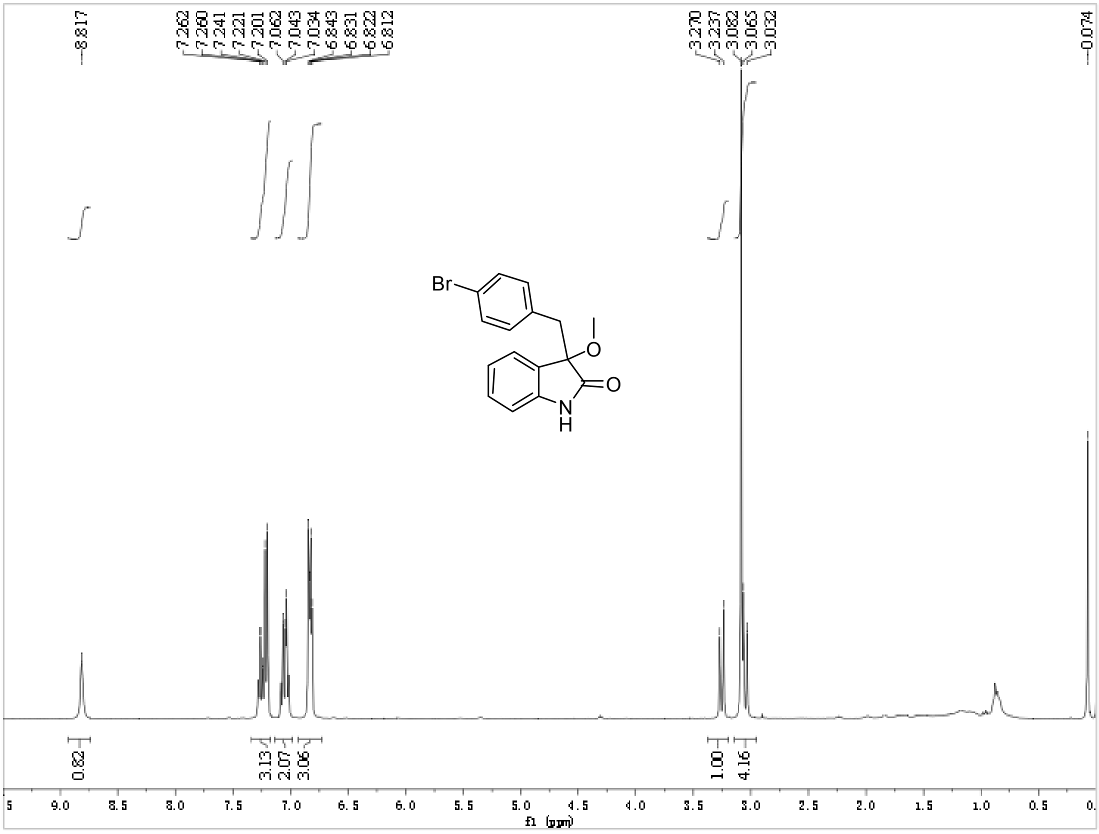

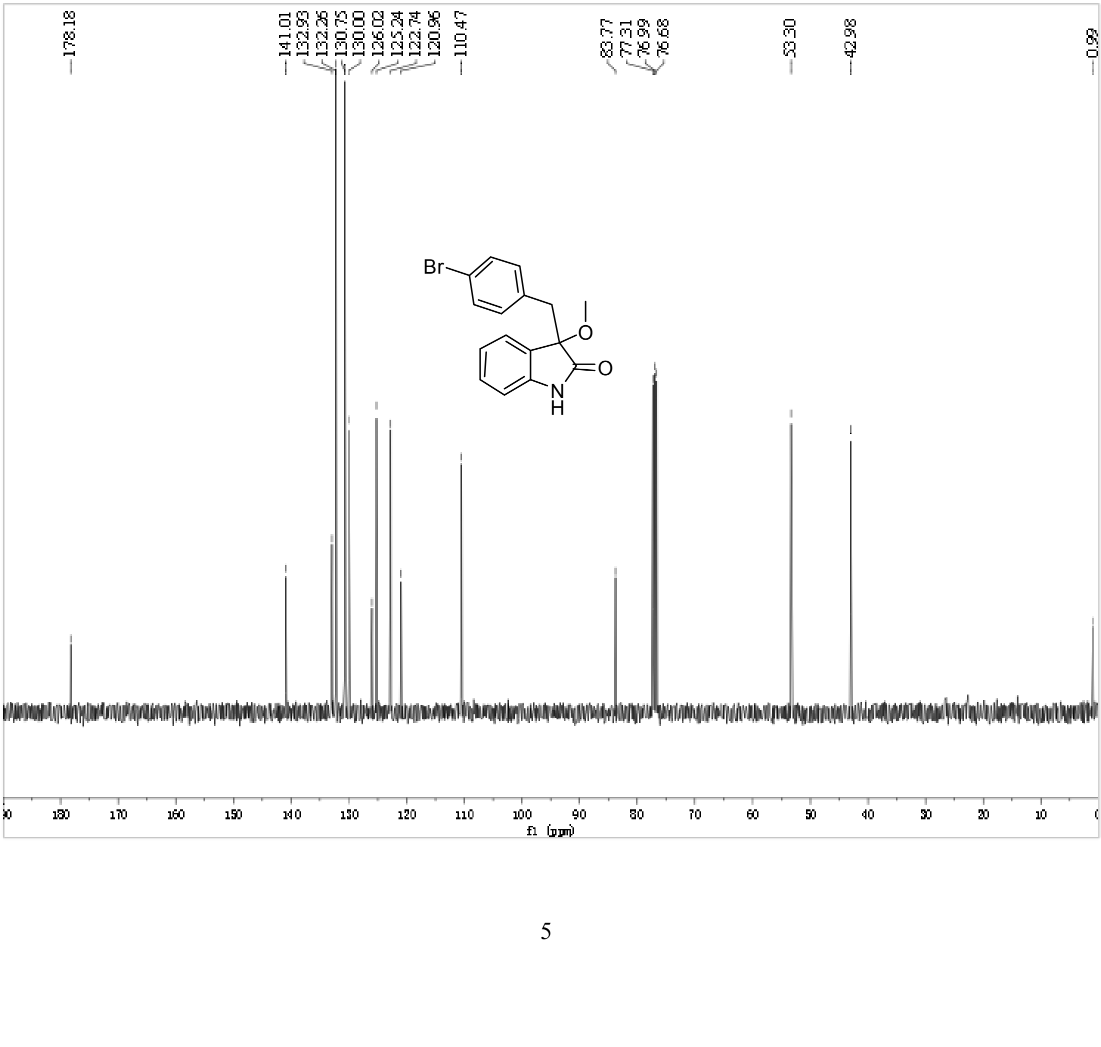

^{1}H and ^{13}C NMR of 3ea

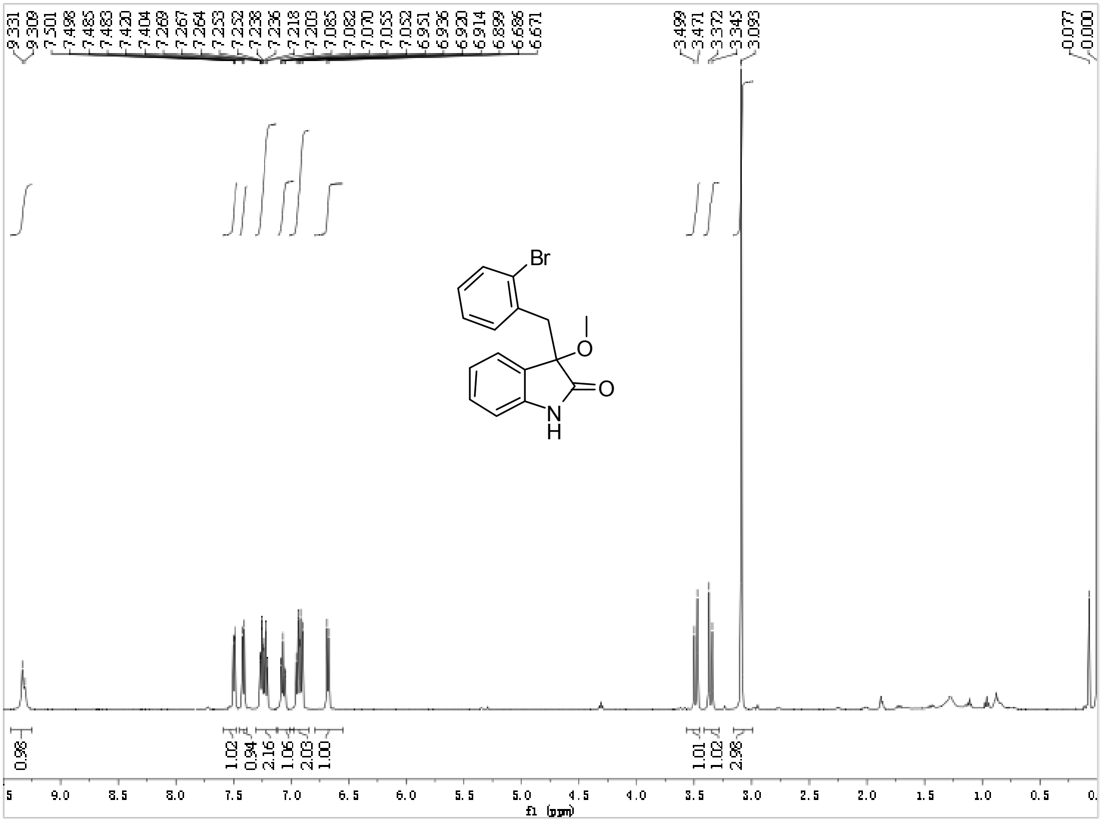

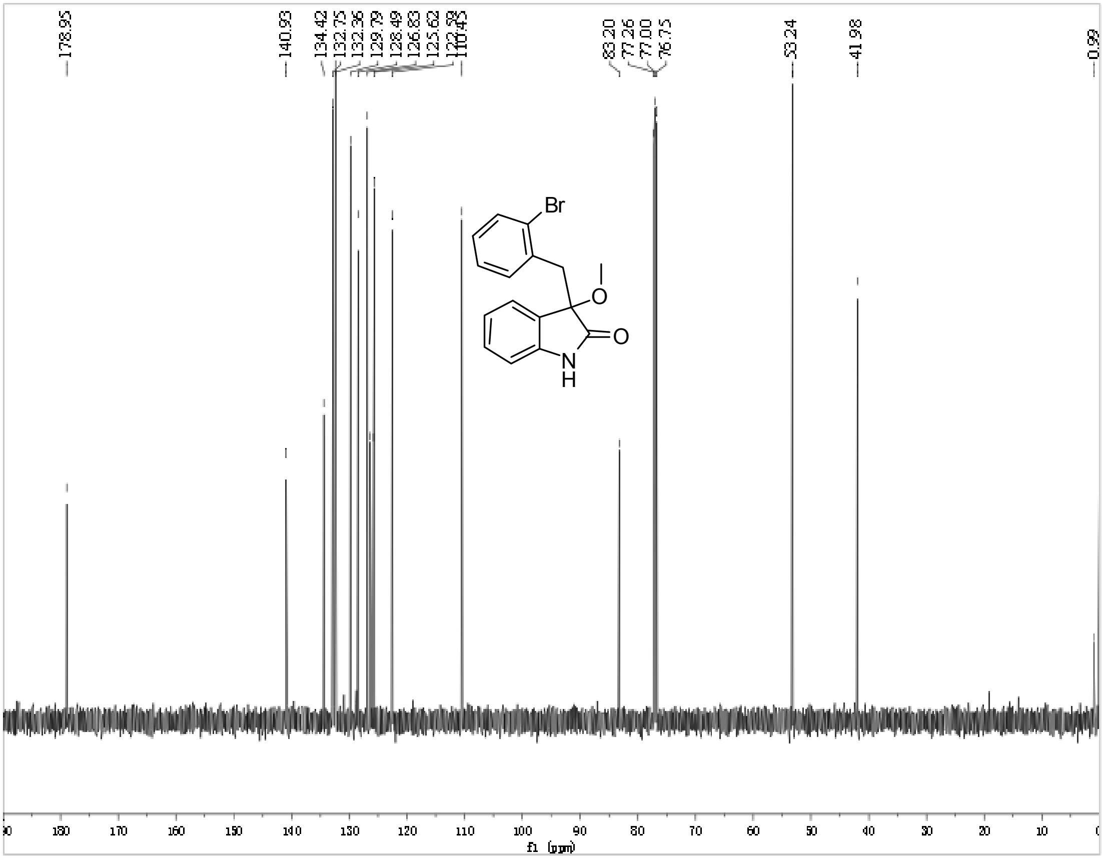

^{1}H and ^{13}C NMR of 3fa

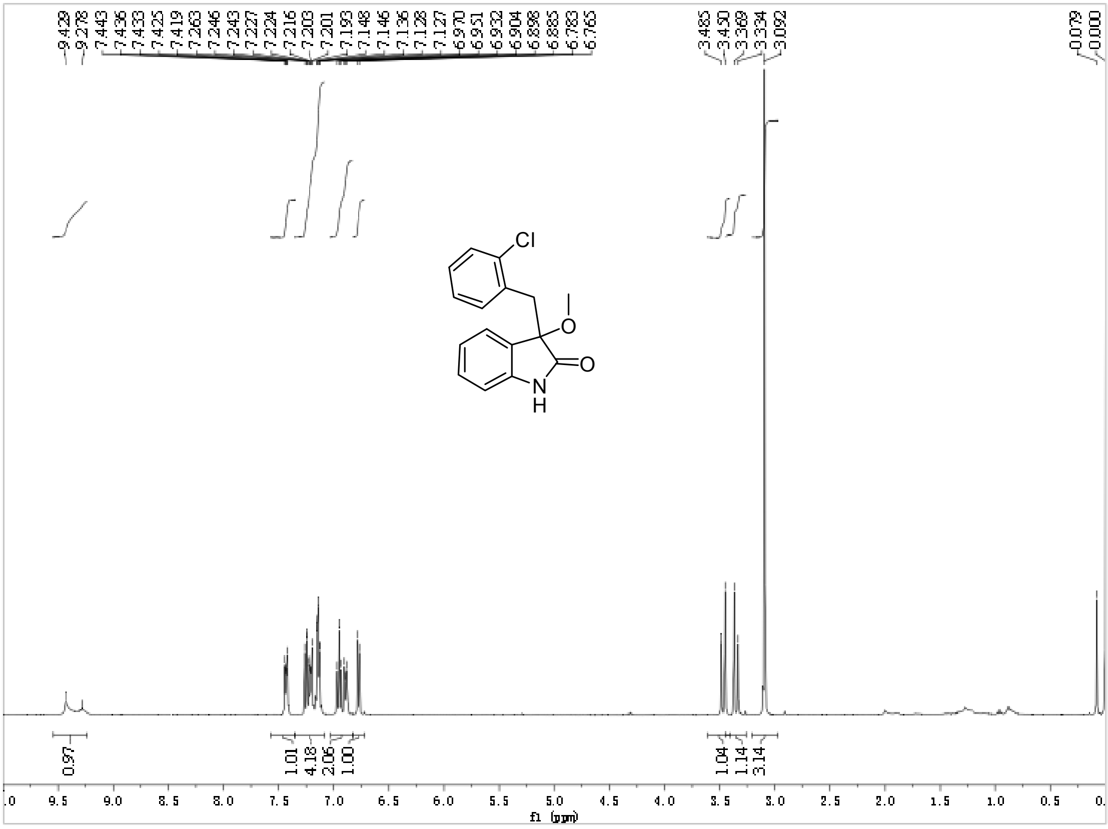

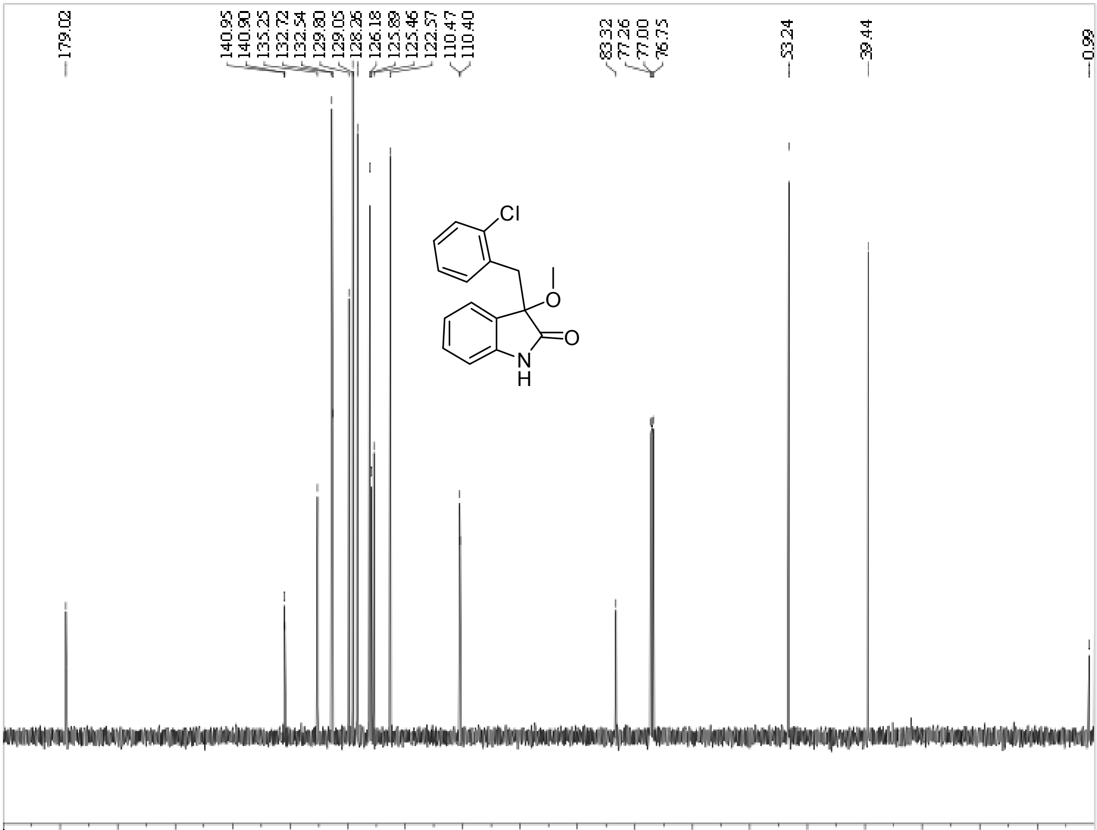

^{1}H and ^{13}C NMR of 3ga

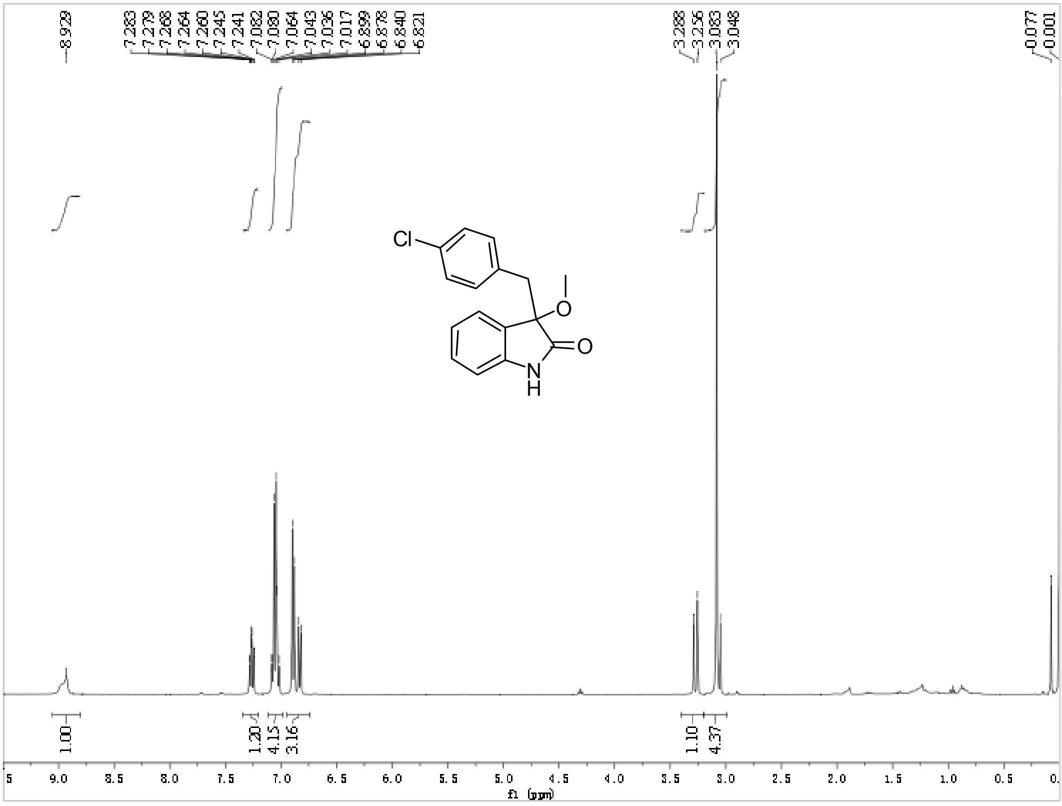

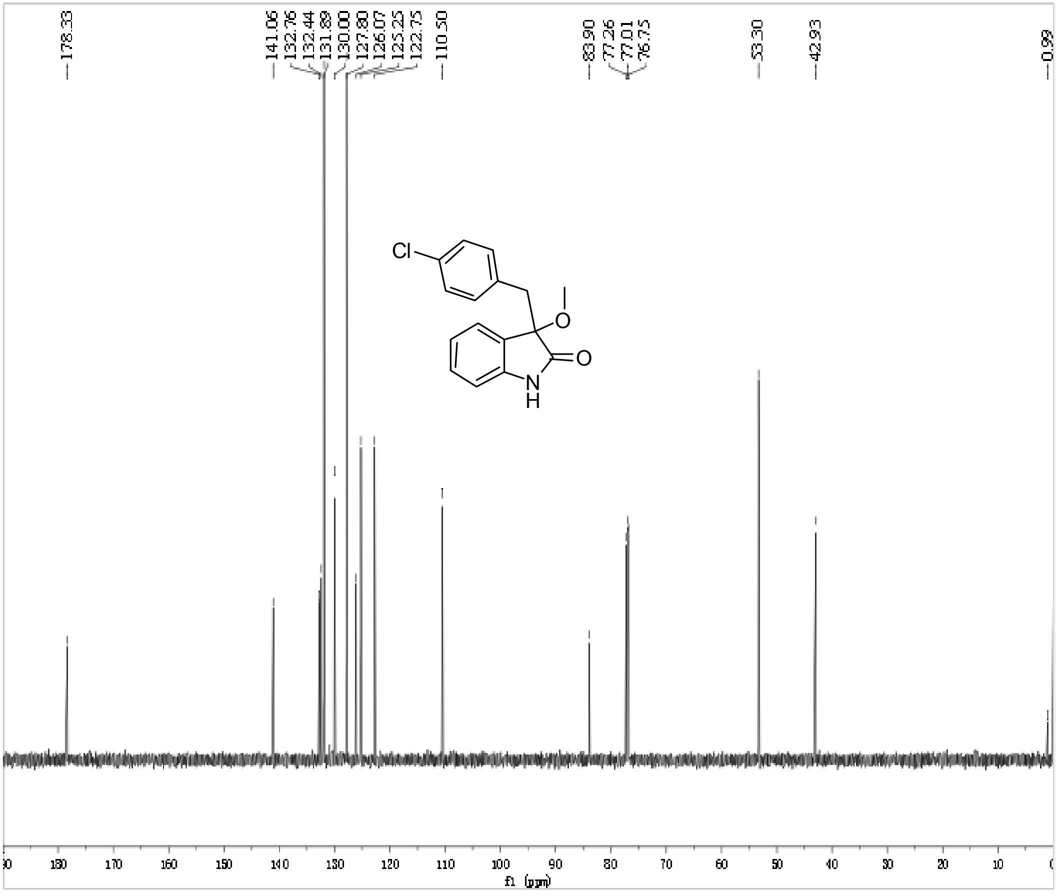

^{1}H and ^{13}C NMR of 3ha

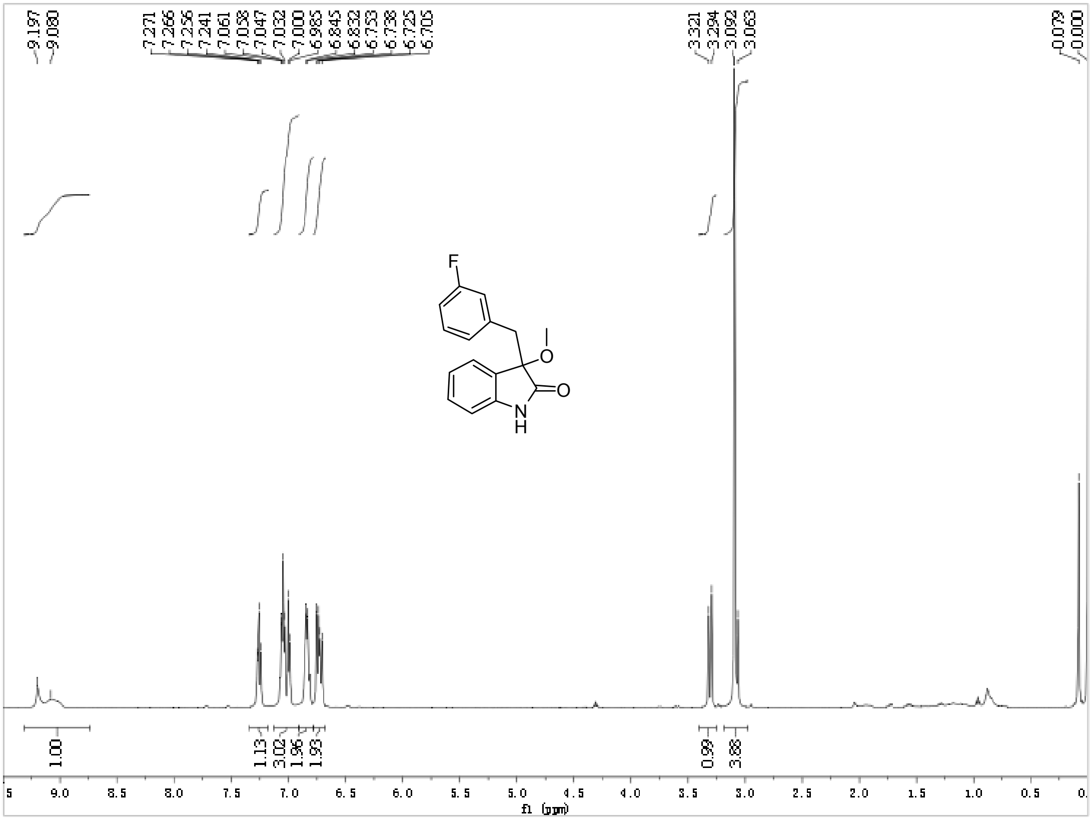

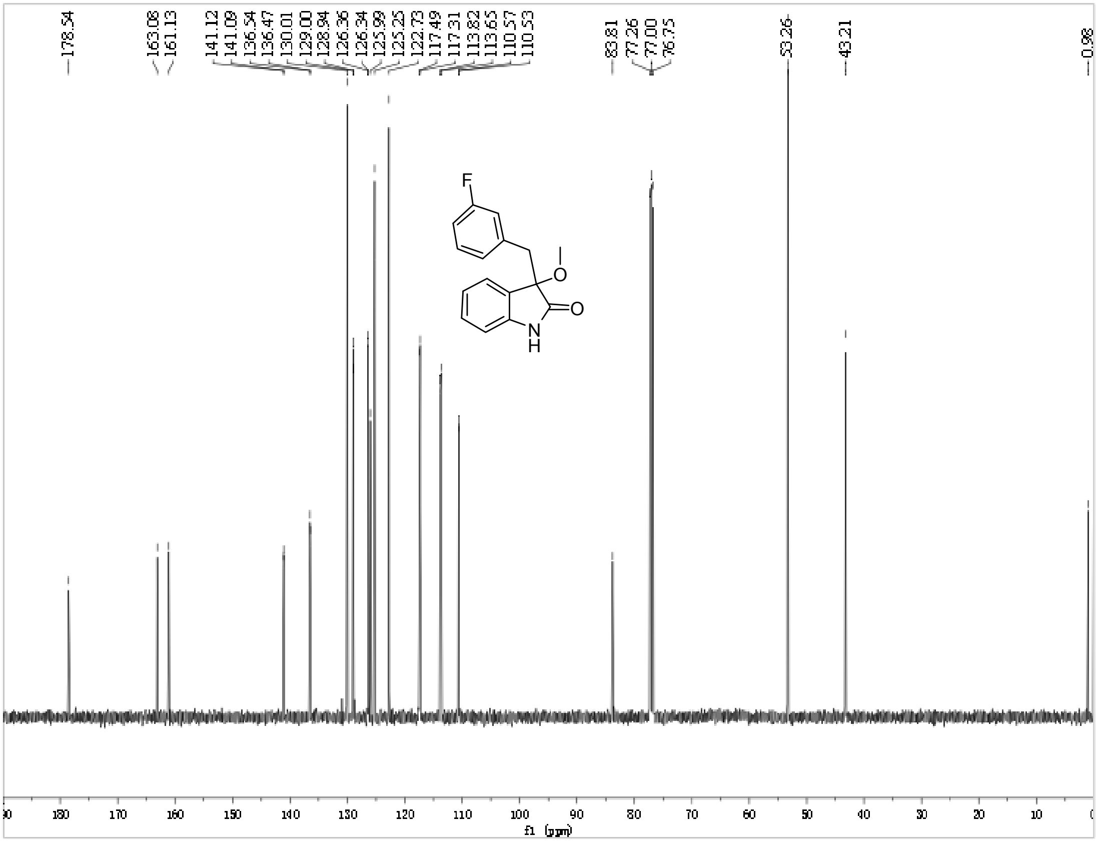

^{1}H and ^{13}C NMR of 3ia

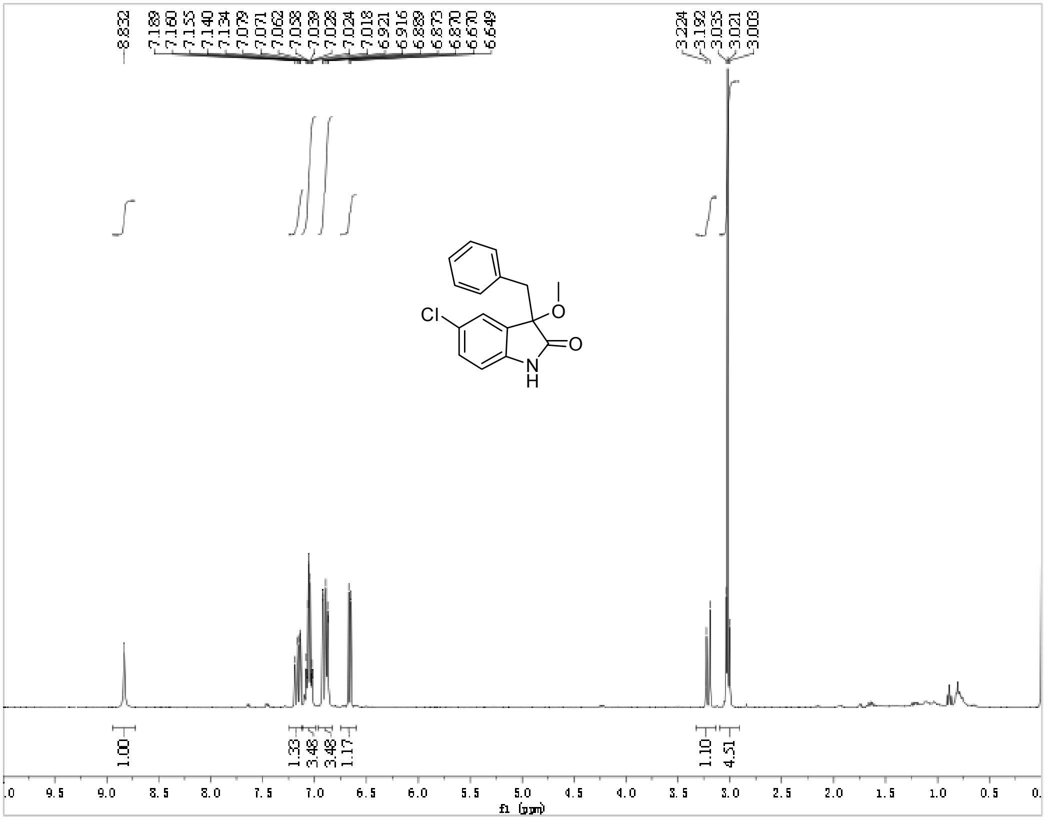

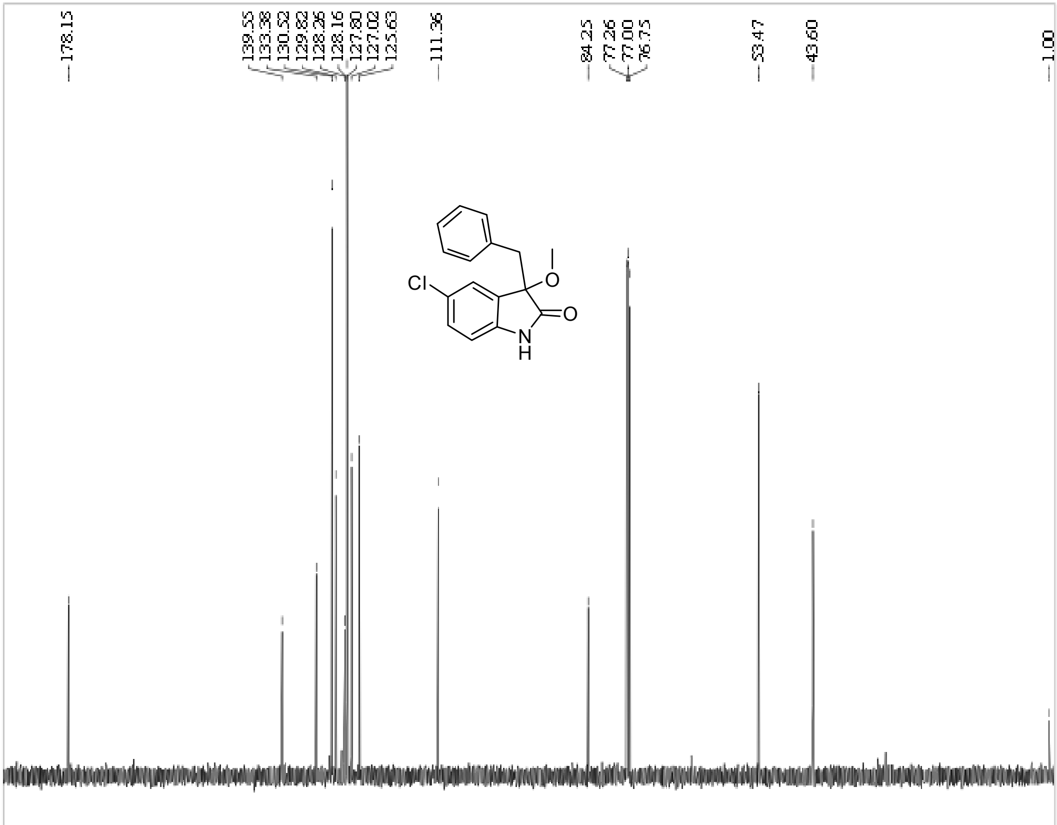

^{1}H and ^{13}C NMR of 3ja

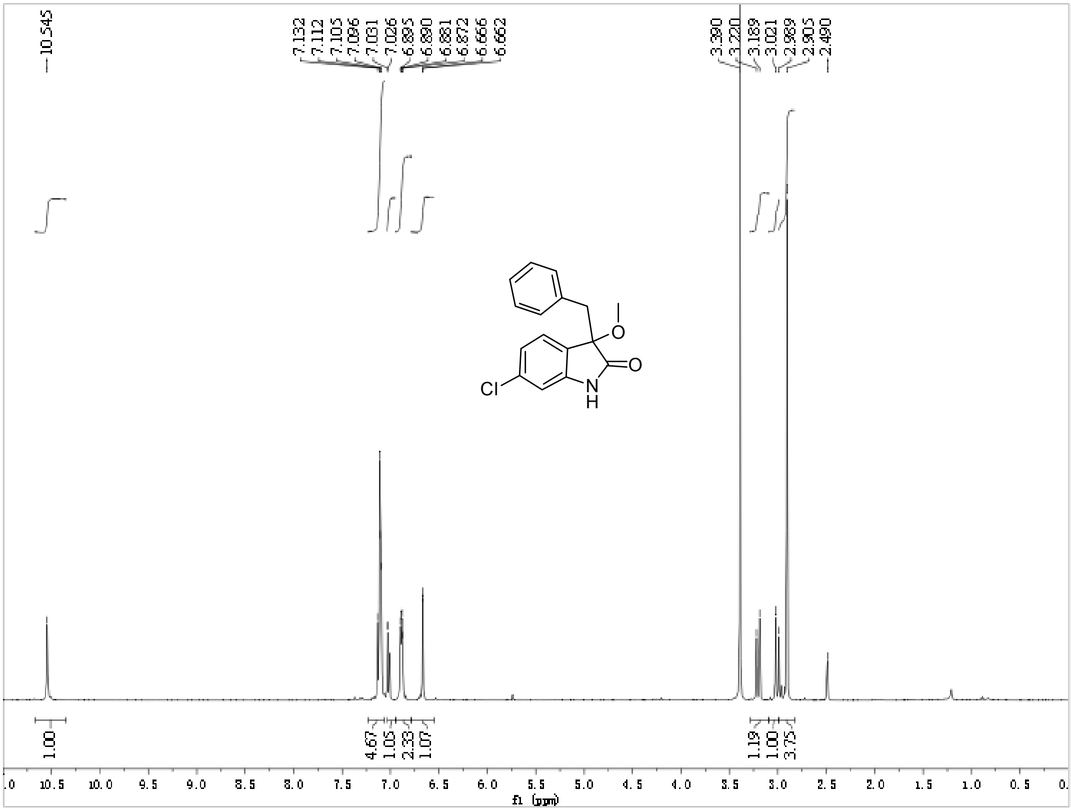

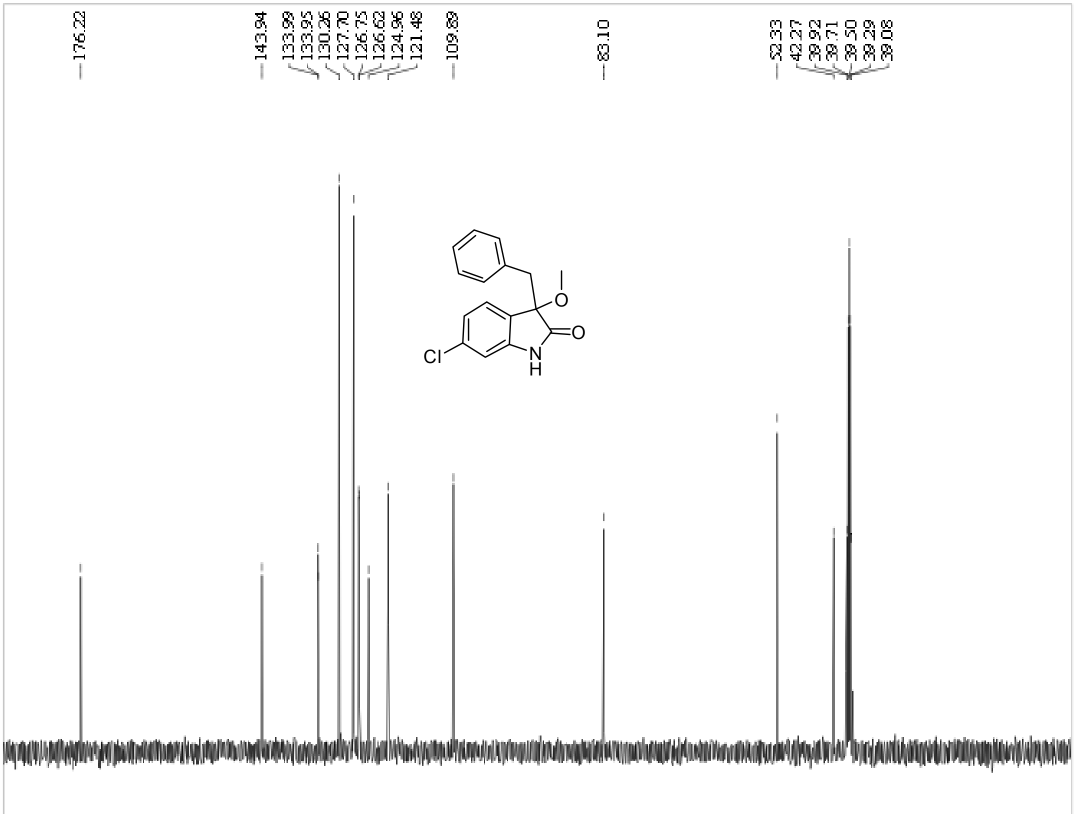

^{1}H and ^{13}C NMR of 3ab

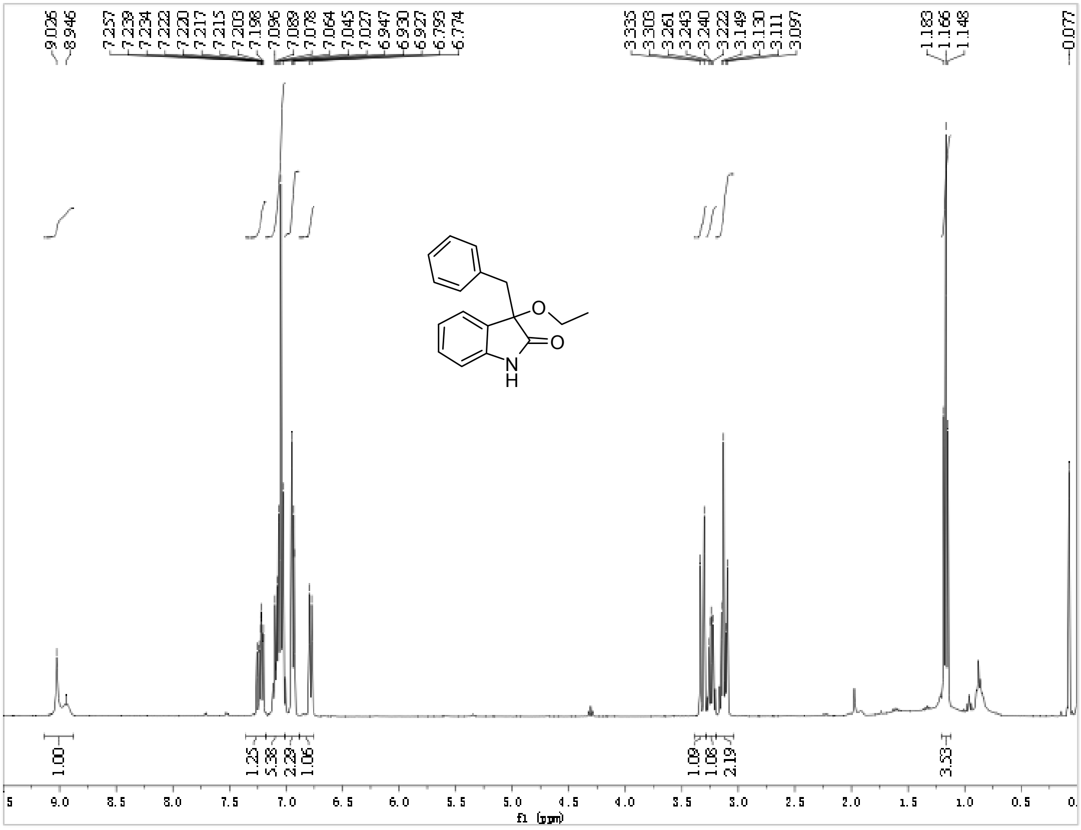

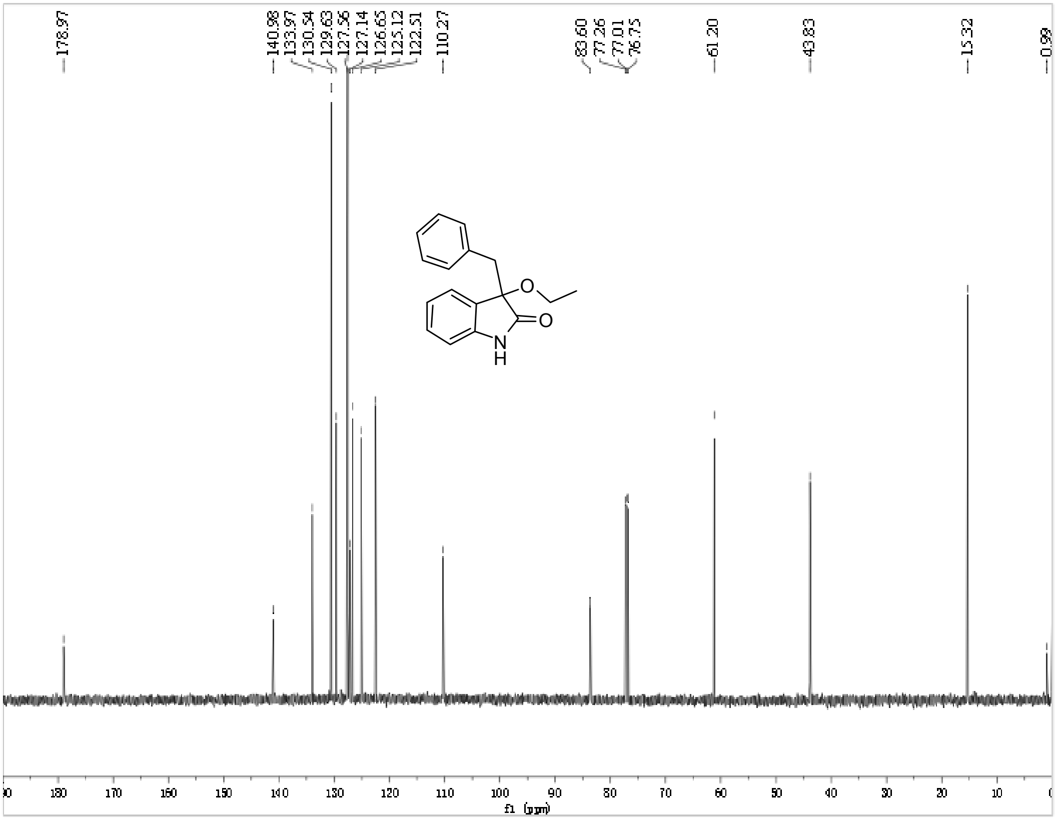

^{1}H and ^{13}C NMR of 3bb

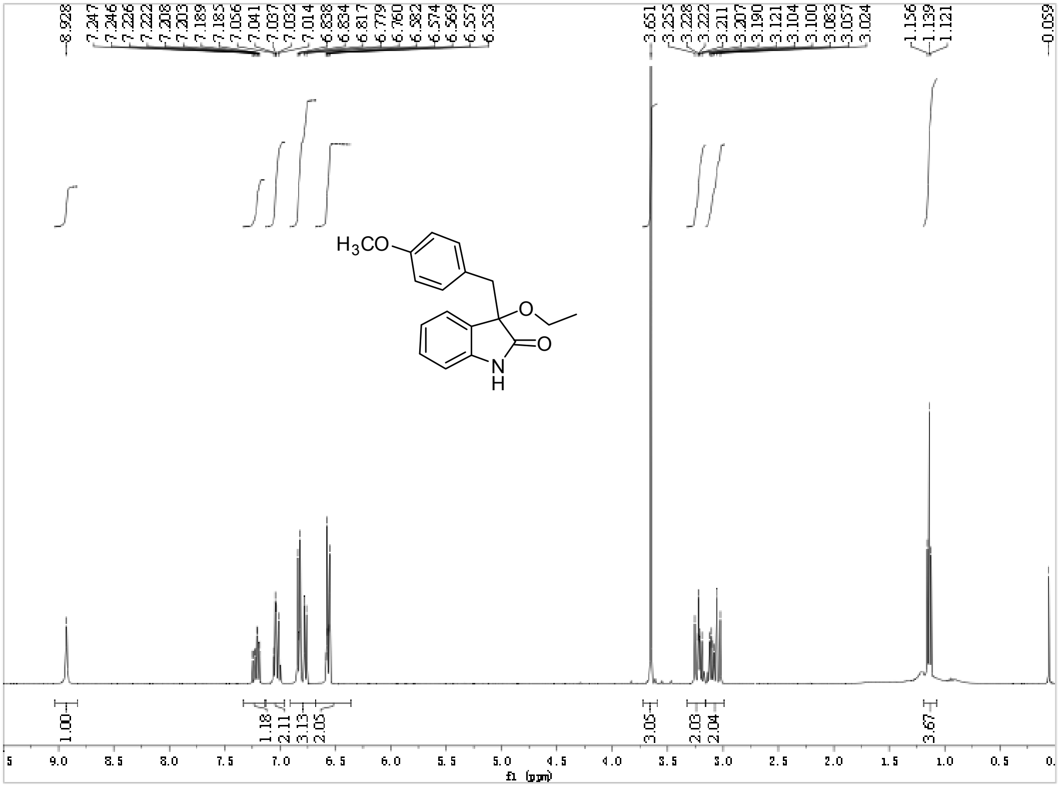

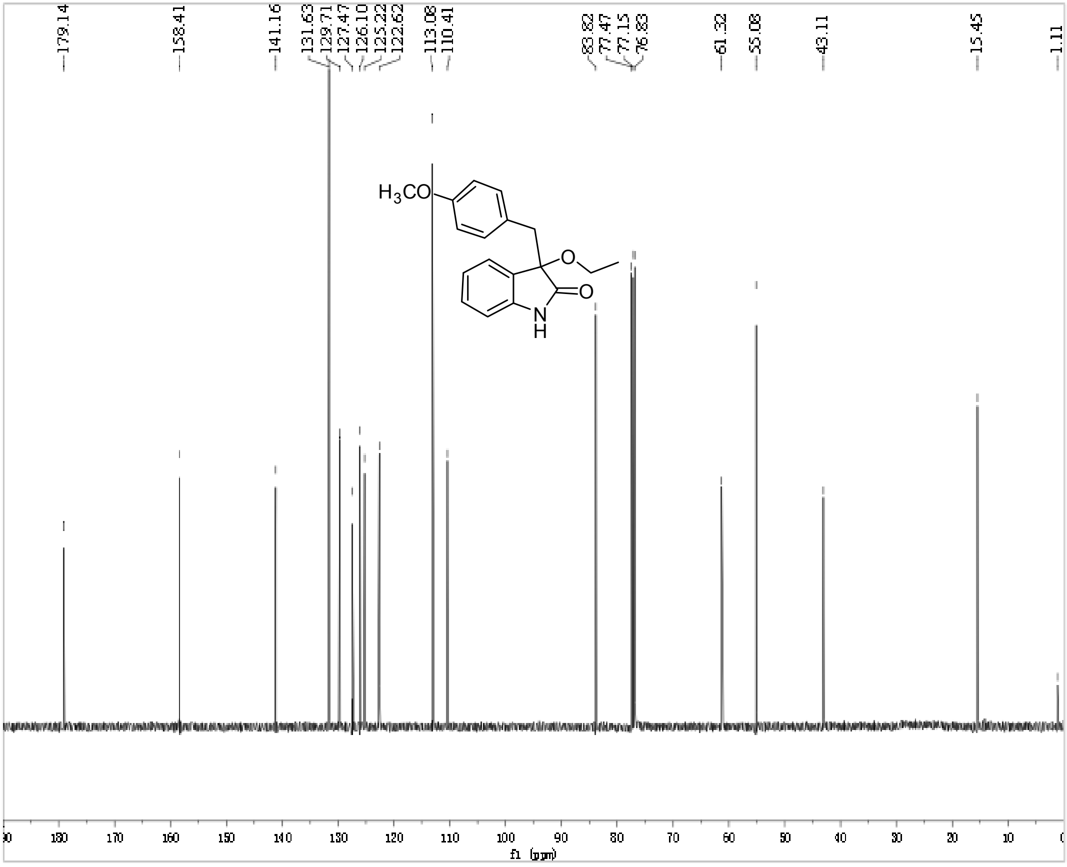

^{1}H and ^{13}C NMR of 3cb

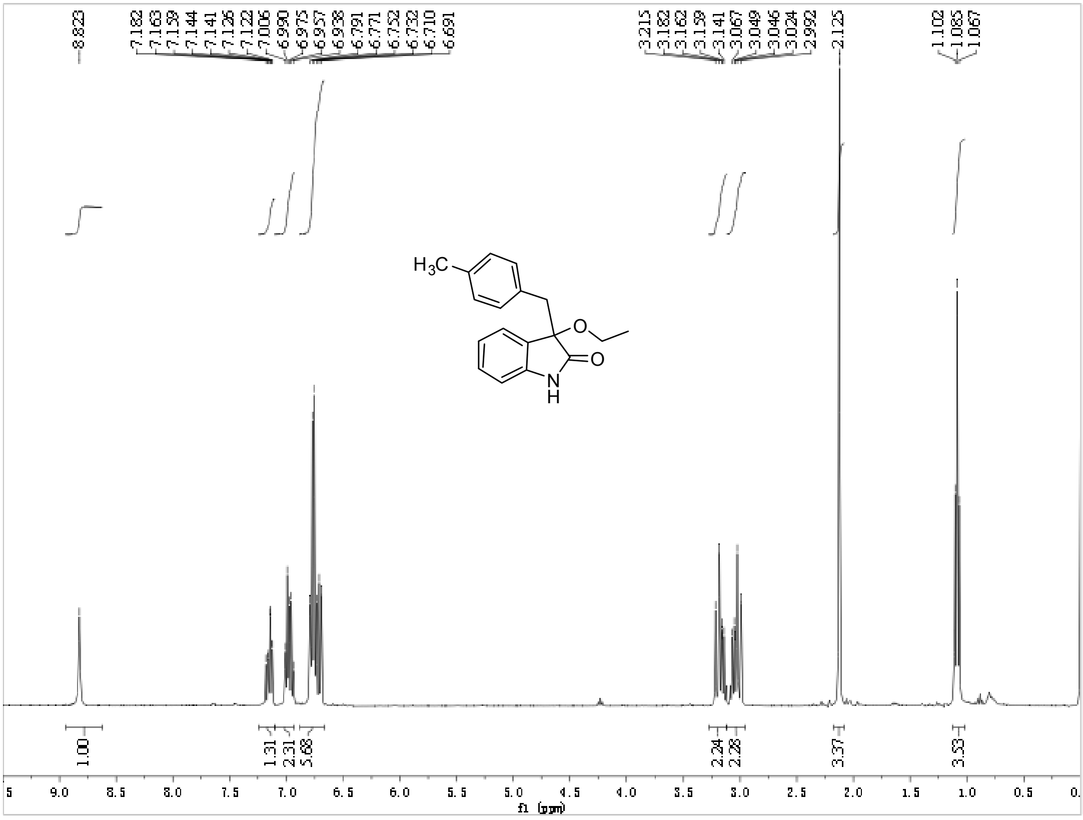

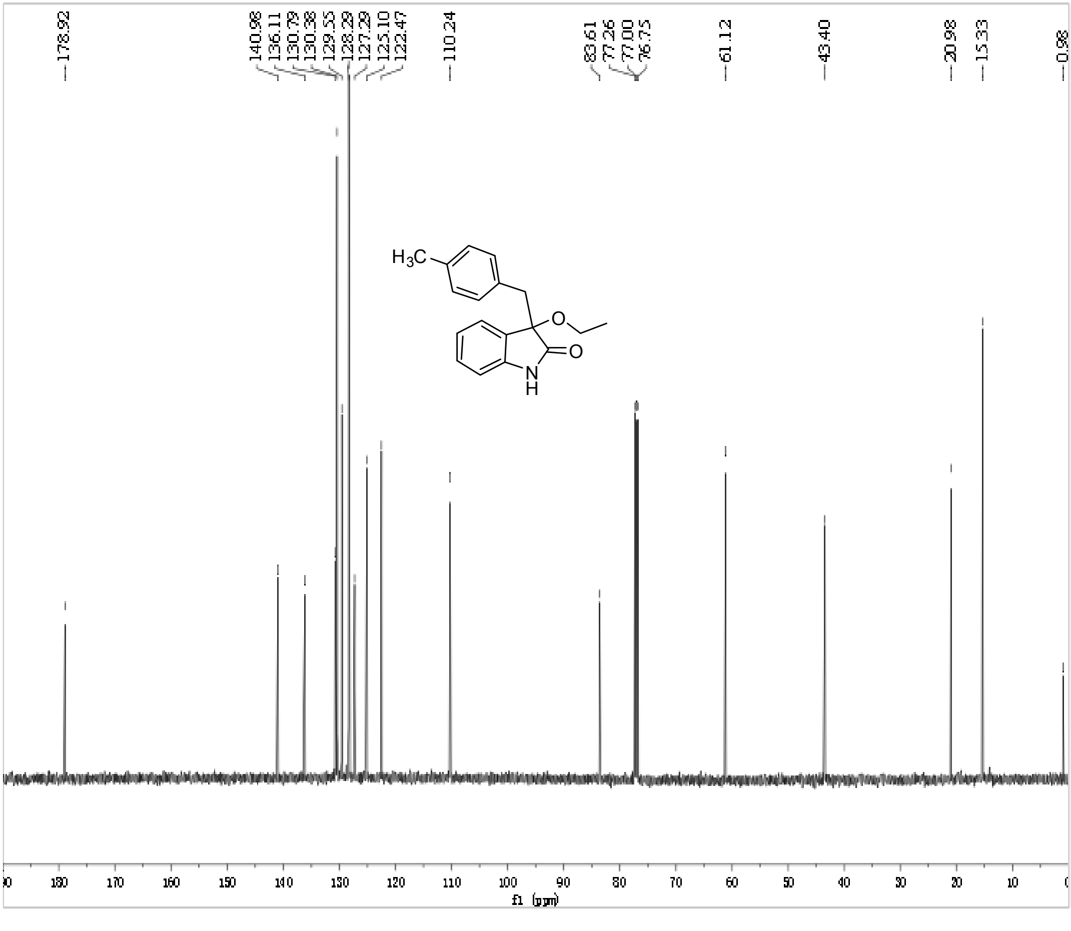

^{1}H and ^{13}C NMR of 3db

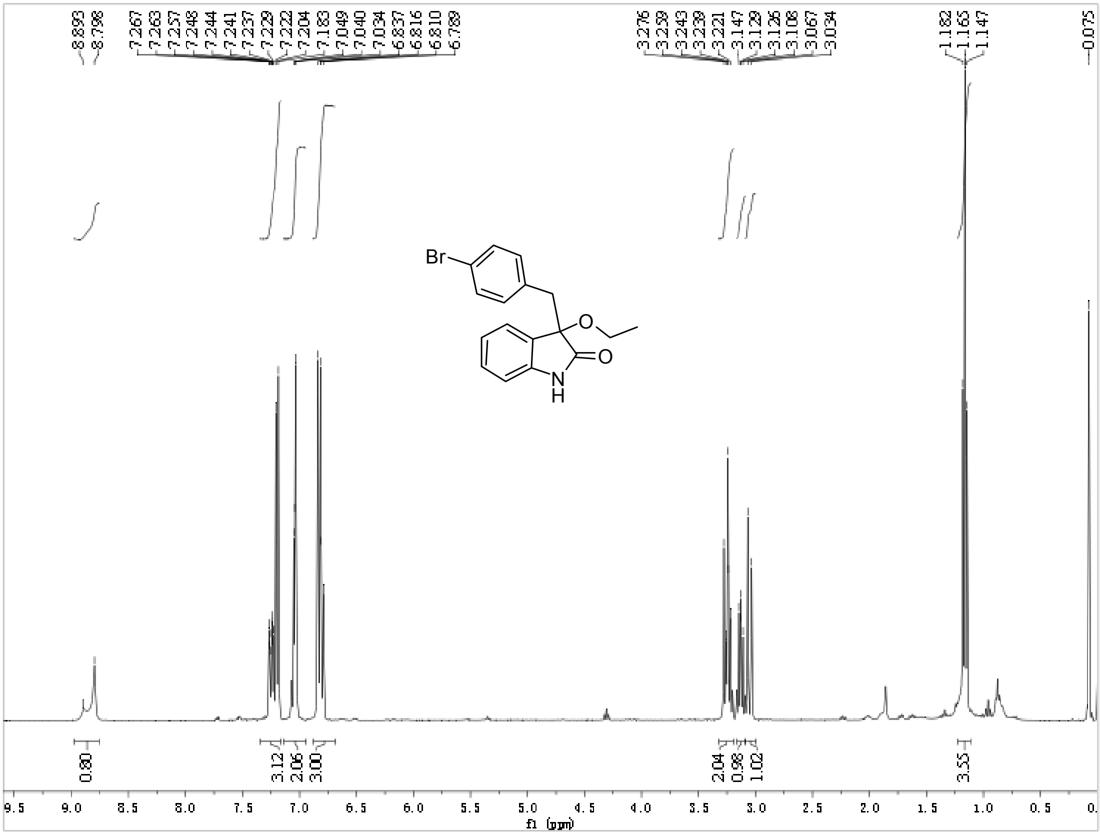

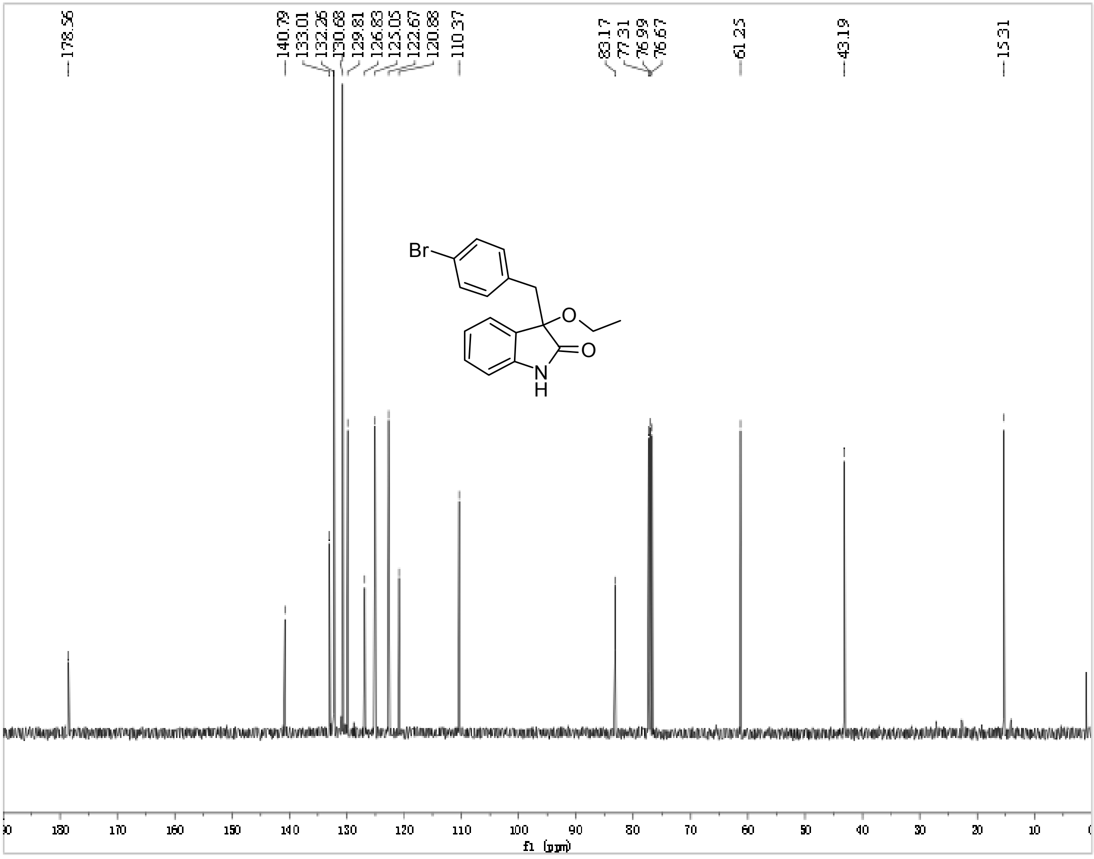

^{1}H and ^{13}C NMR of 3eb

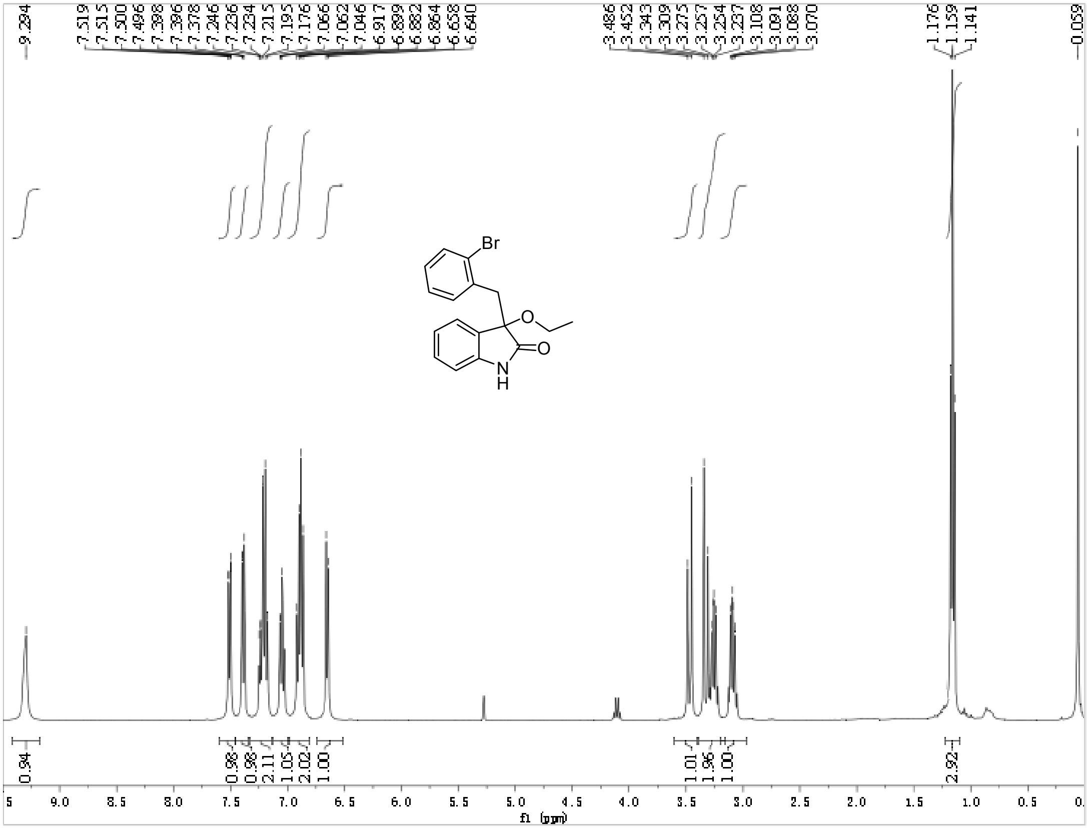

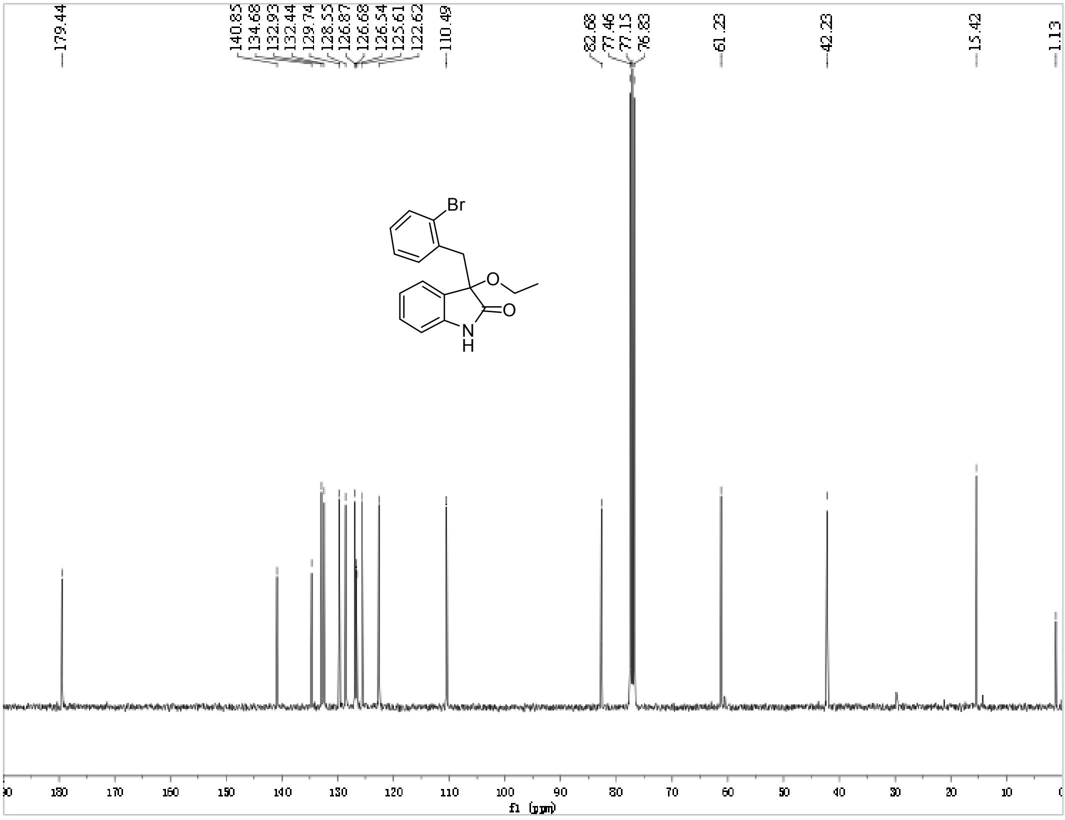

^{1}H and ^{13}C NMR of 3fb

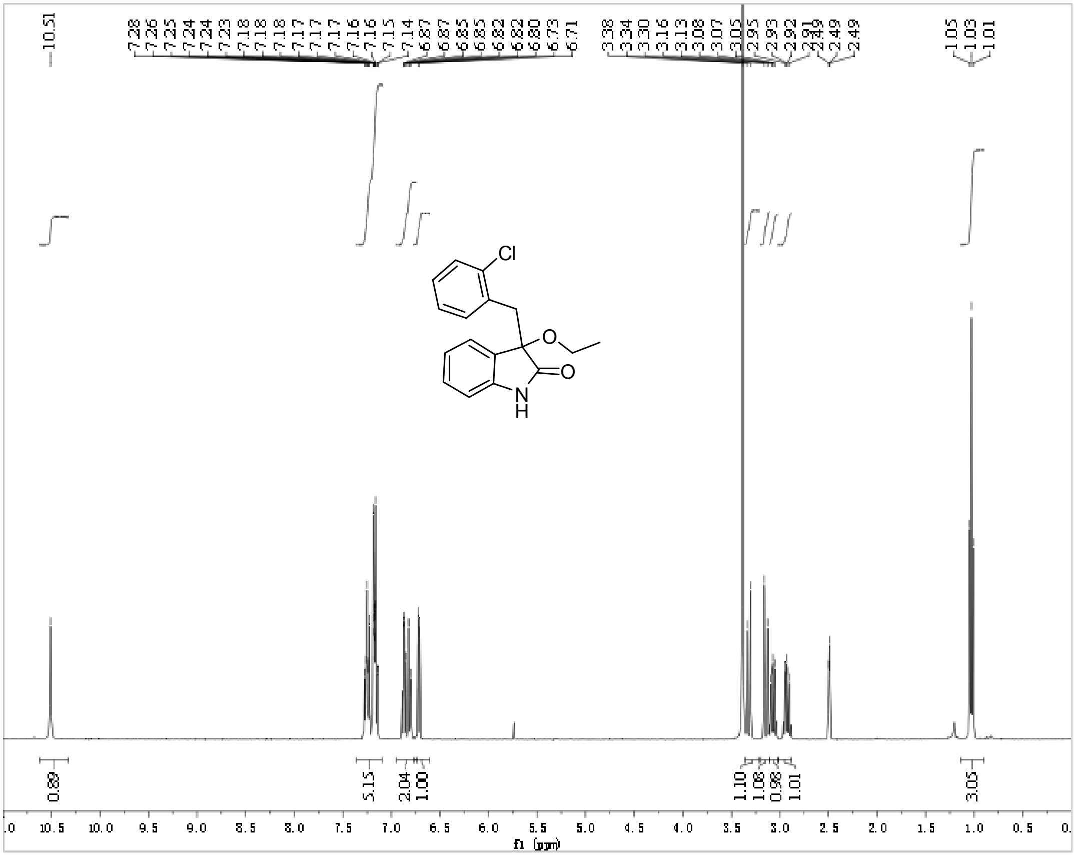

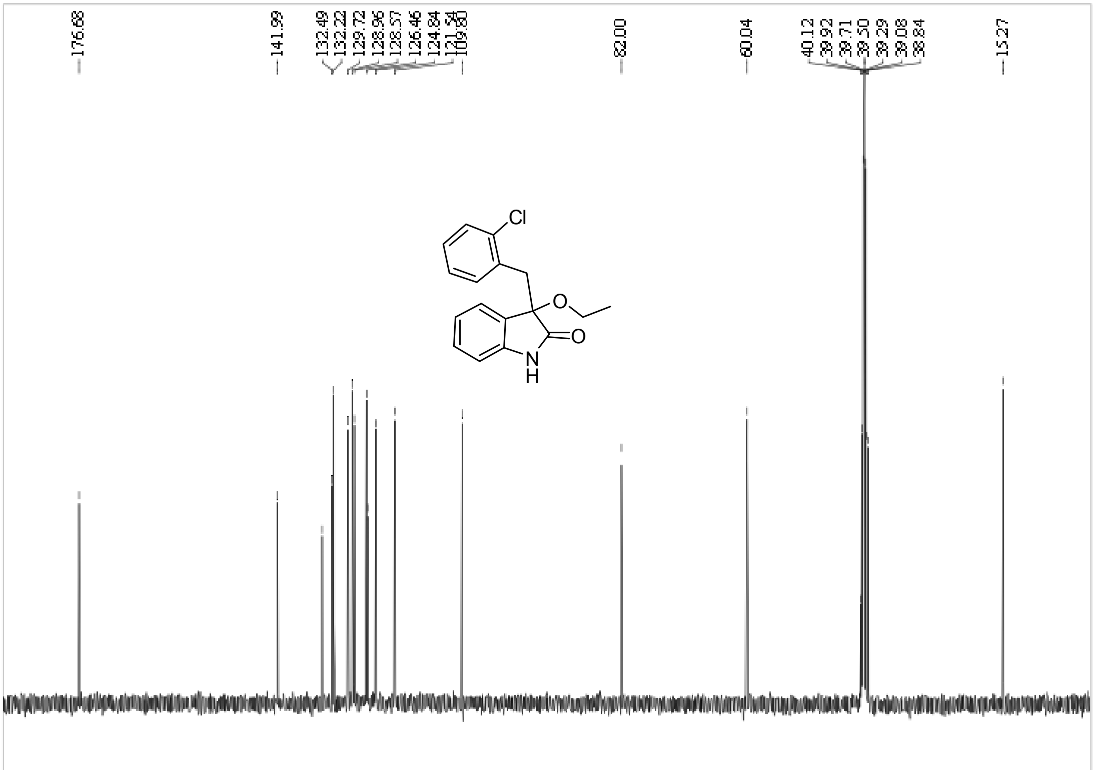

^{1}H and ^{13}C NMR of 3gb

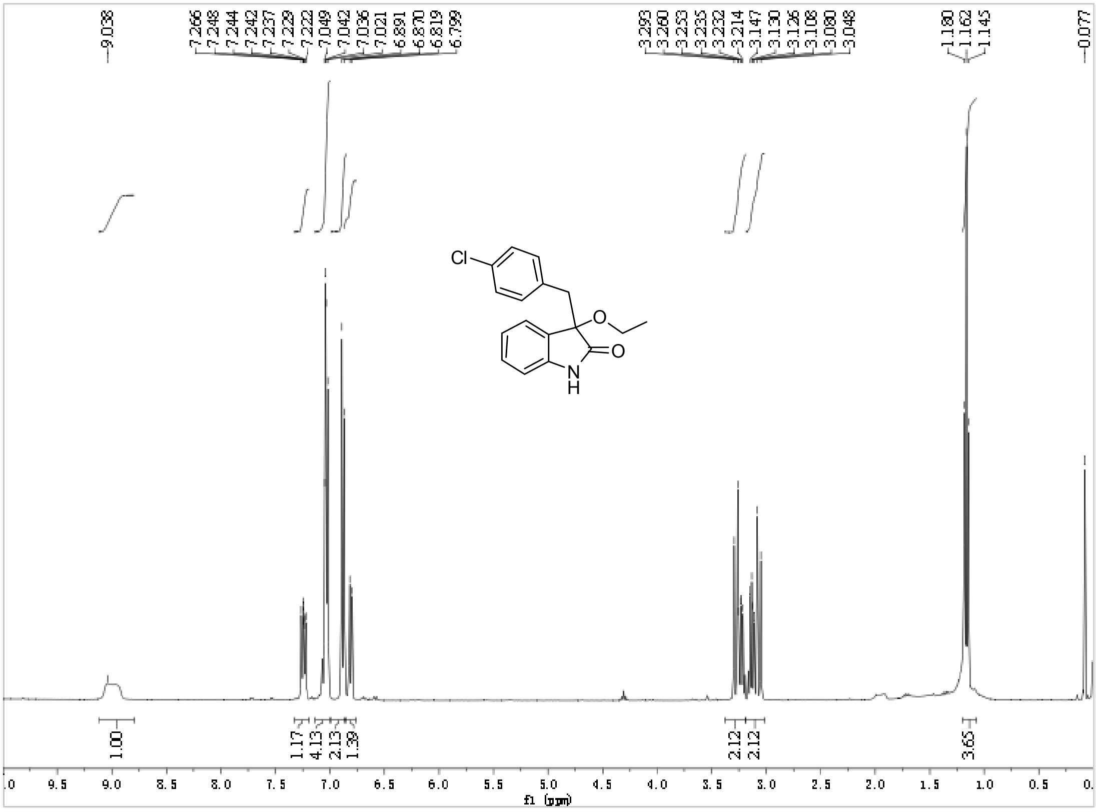

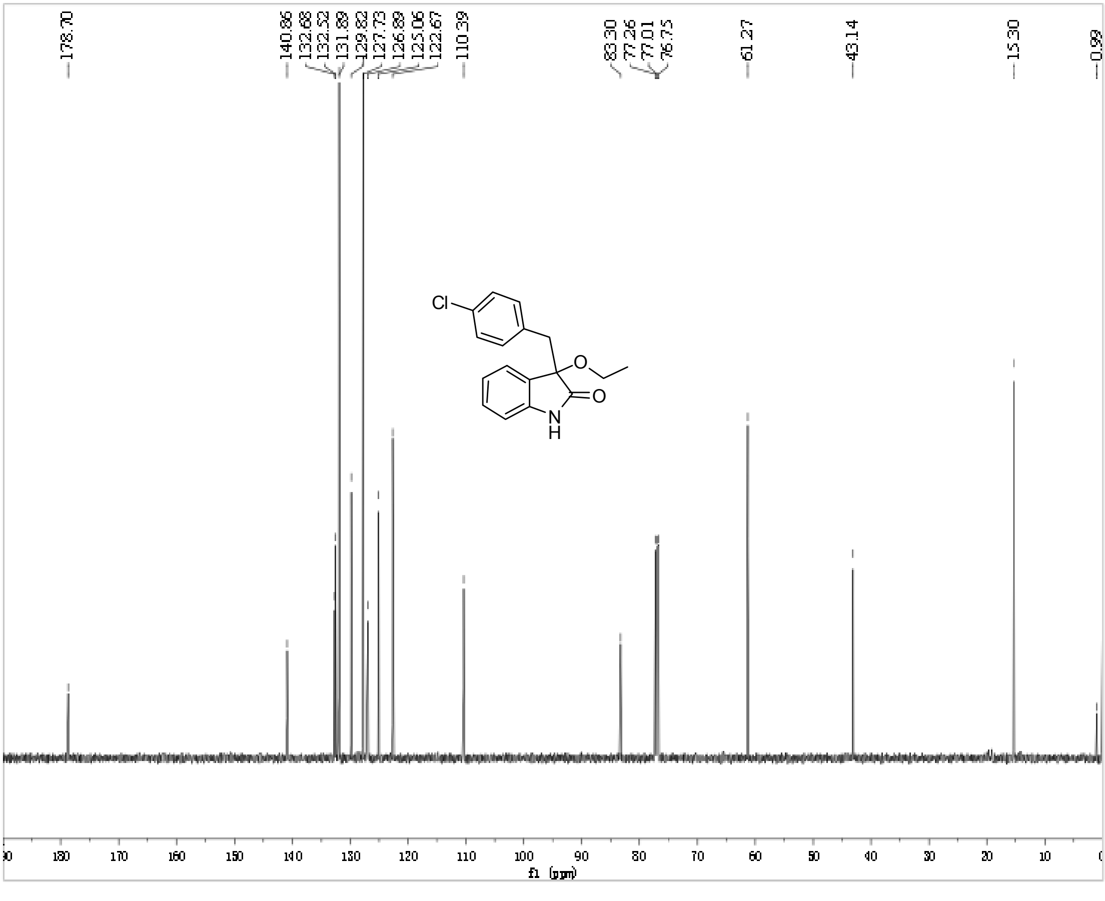

^{1}H and ^{13}C NMR of 3hb

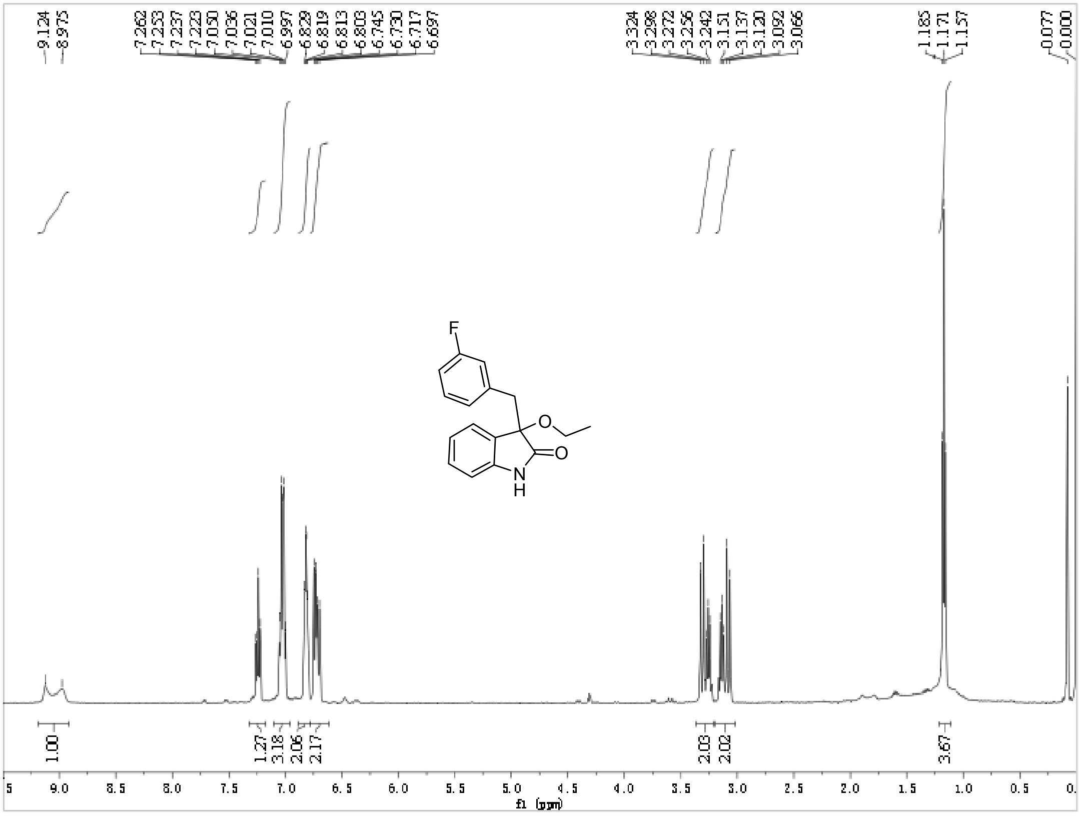

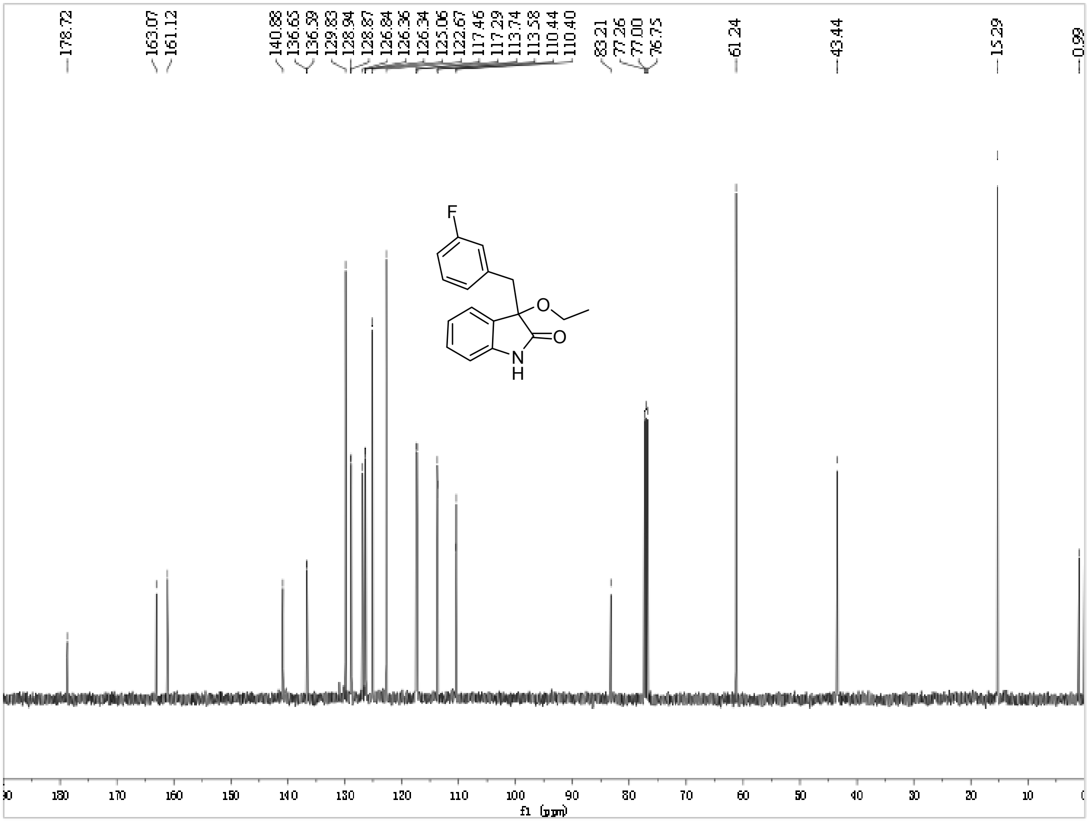

^{1}H and ^{13}C NMR of 3ac

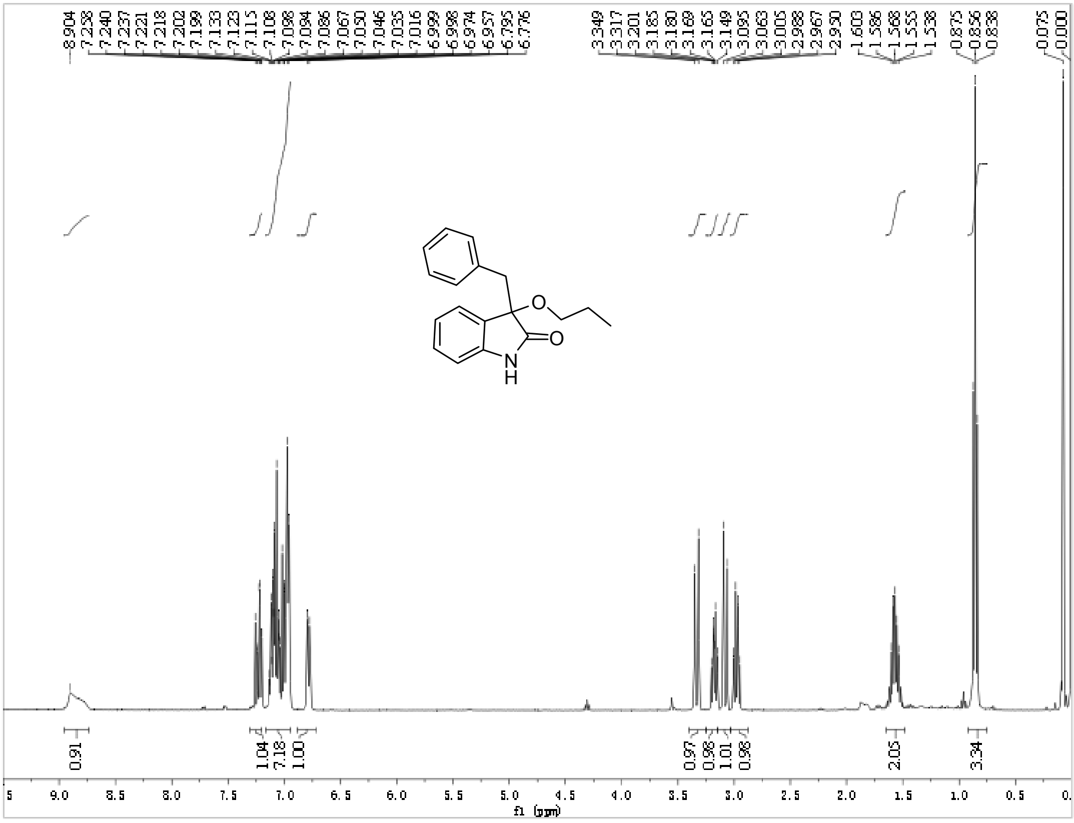

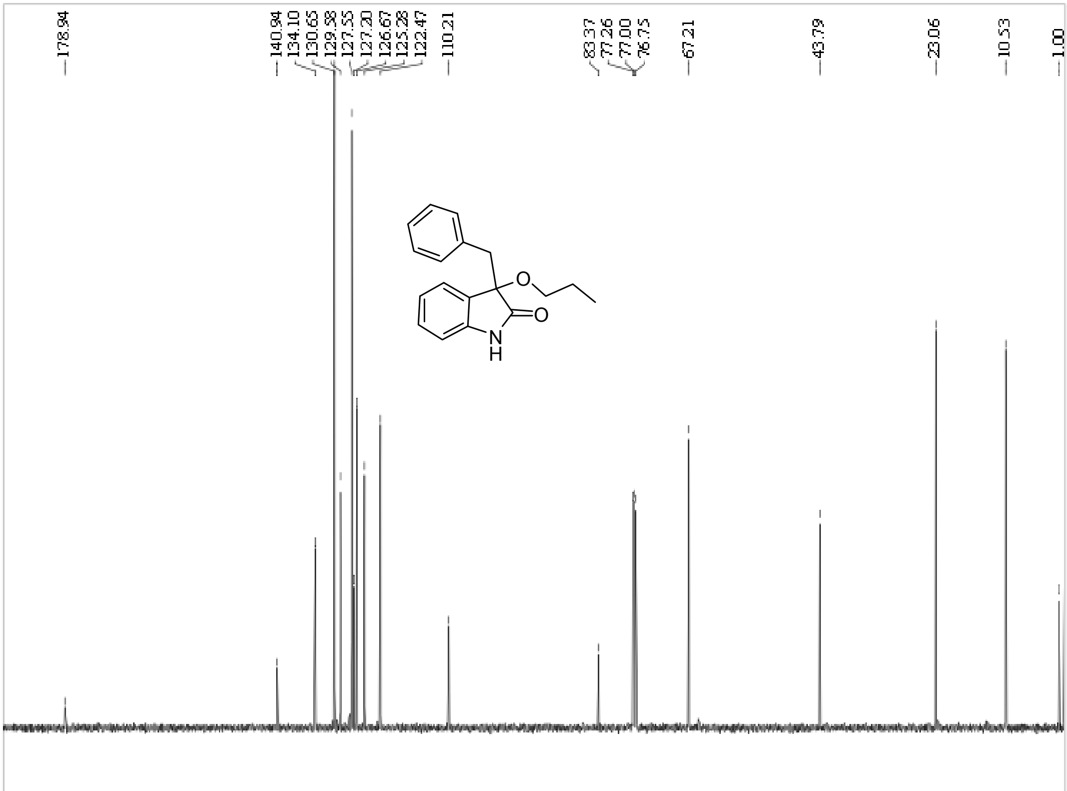

^{1}H and ^{13}C NMR of 3bc

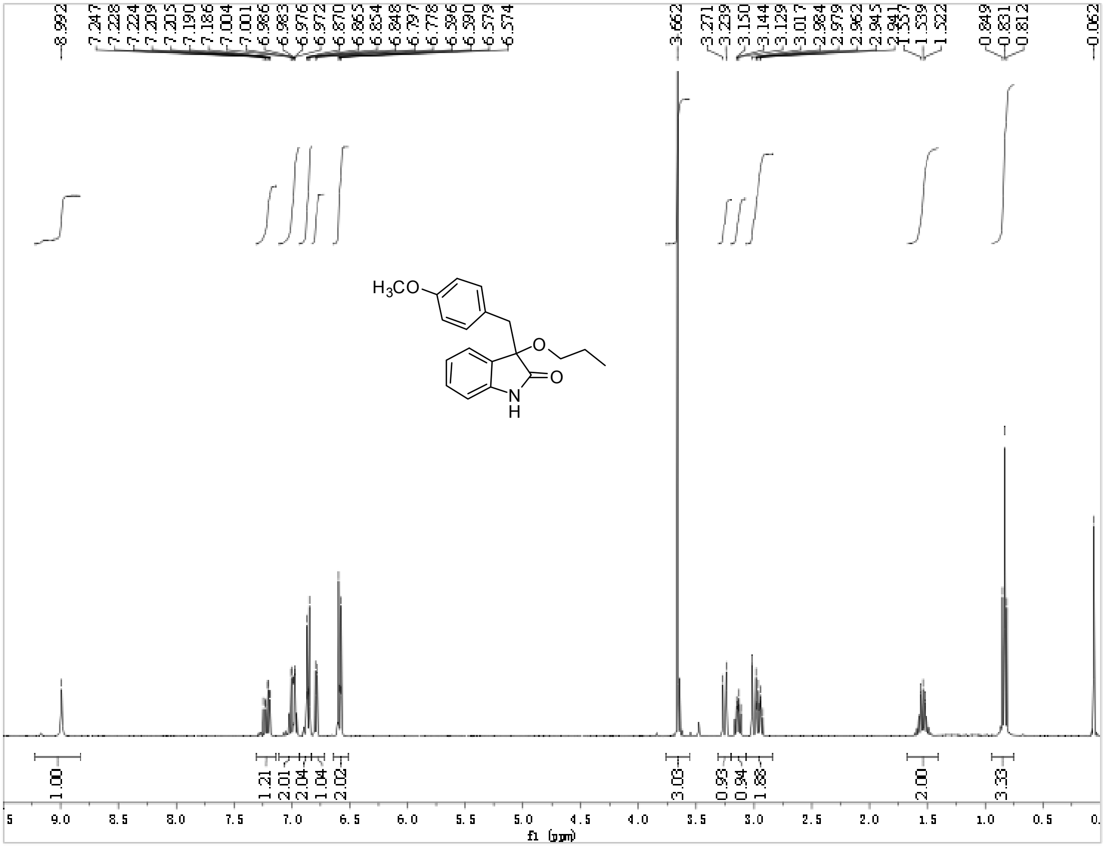

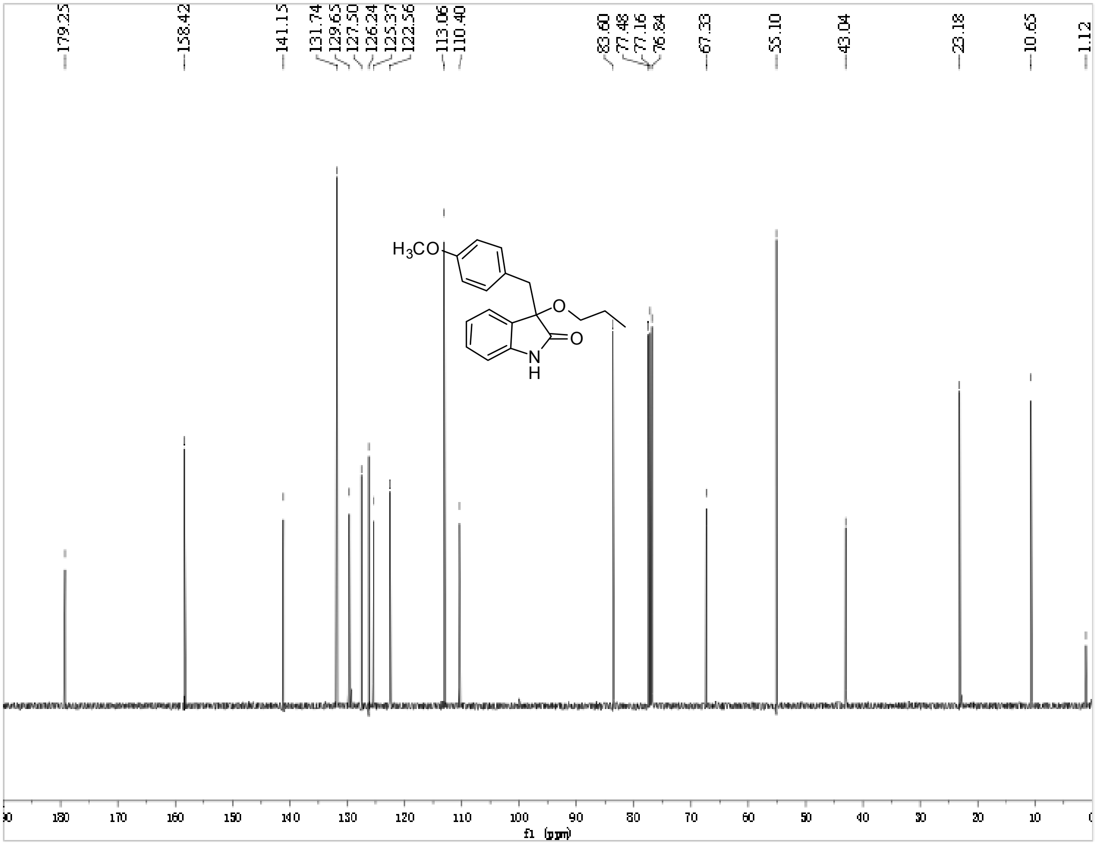

^{1}H and ^{13}C NMR of 3cc

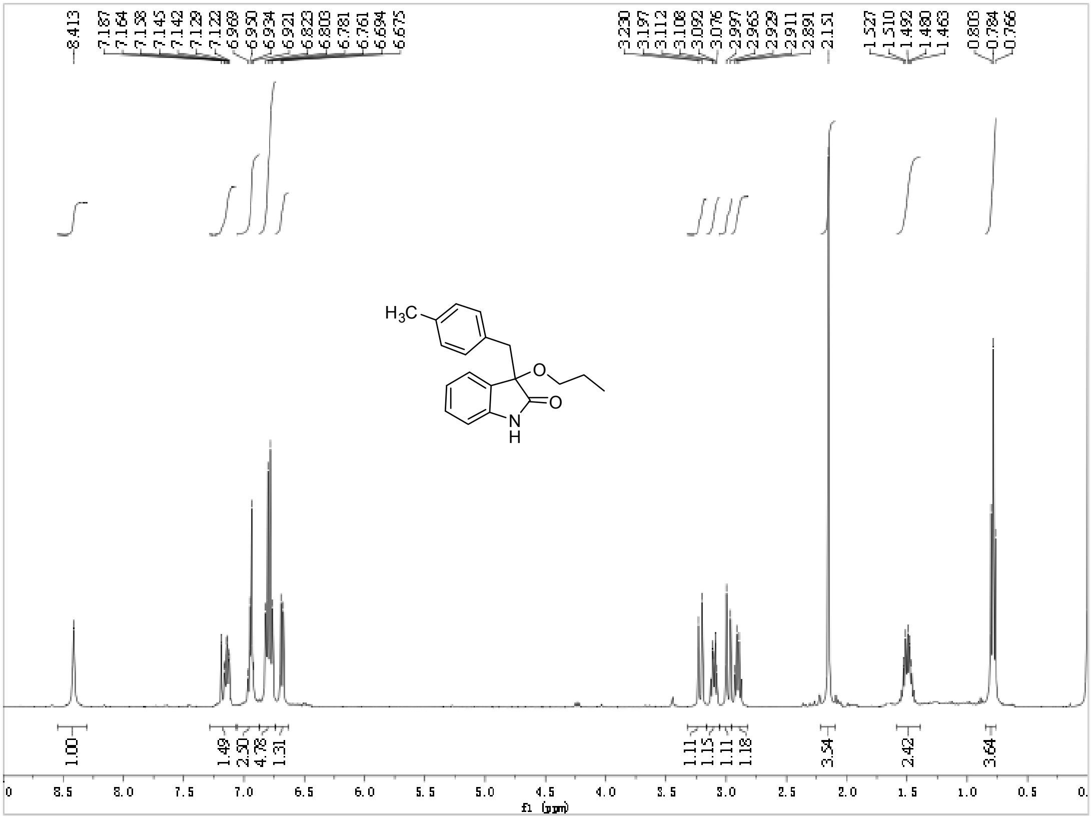

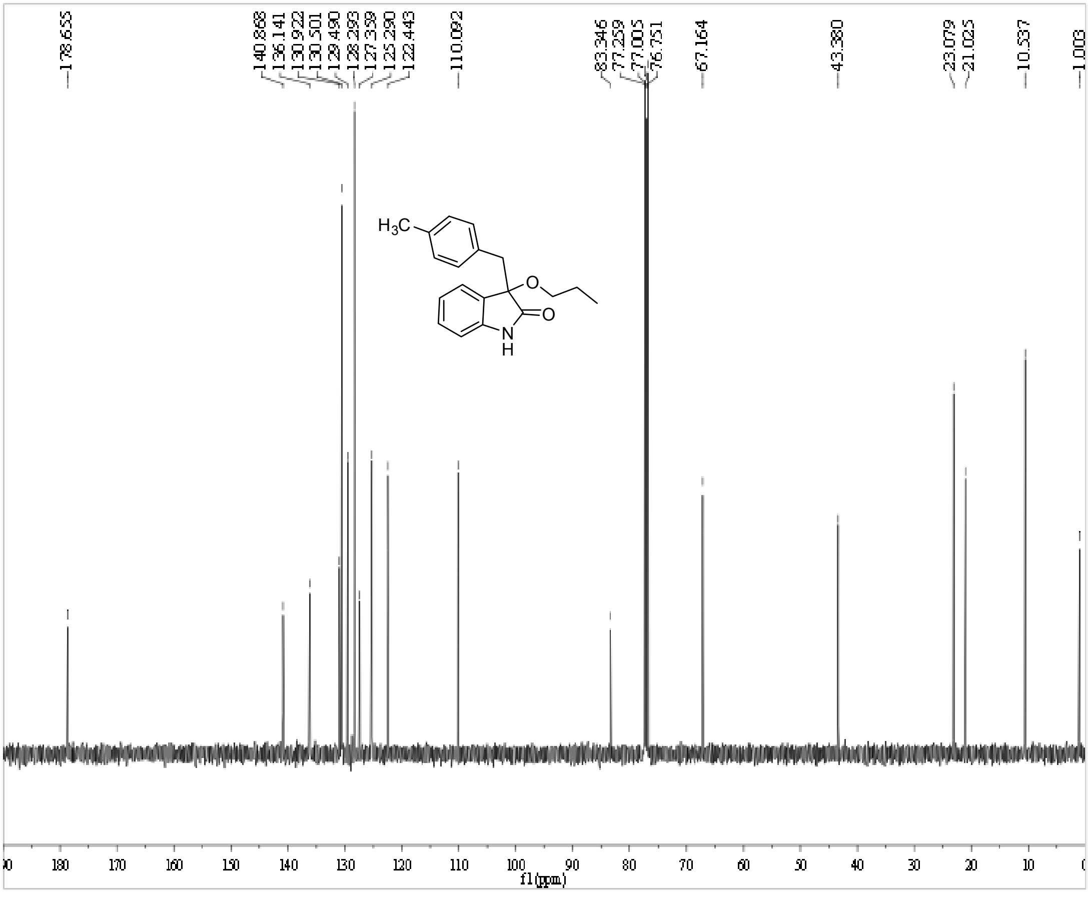

^{1}H and ^{13}C NMR of 3dc

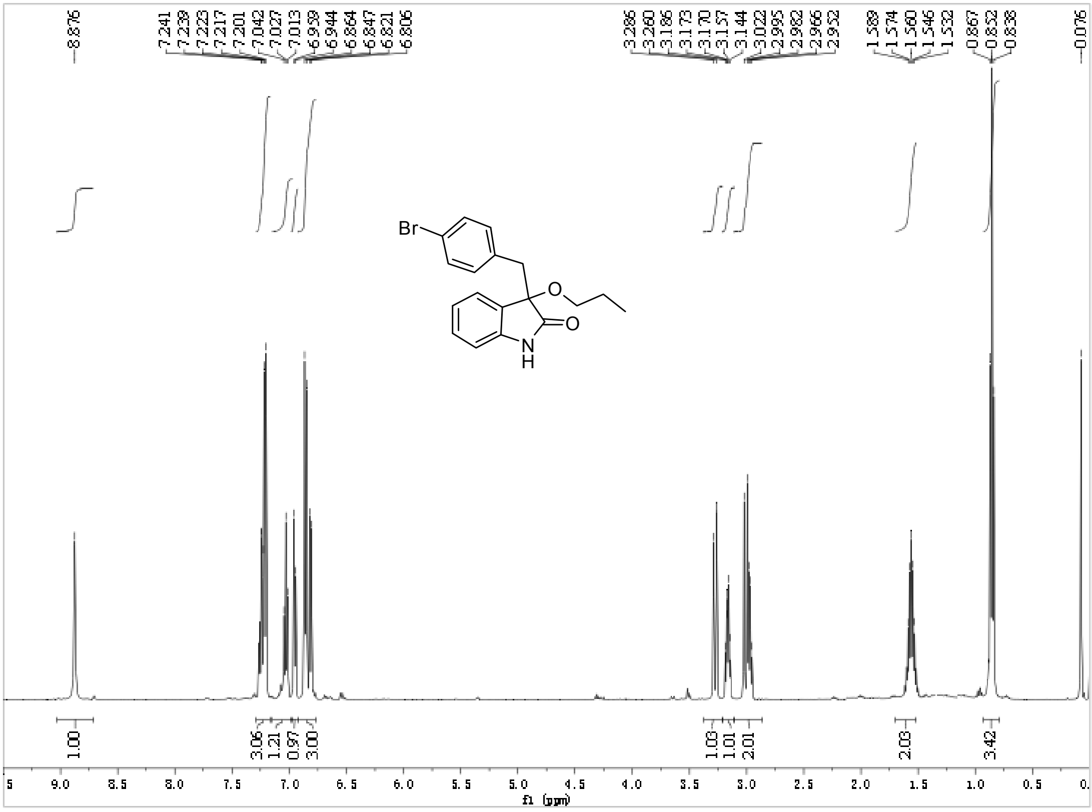

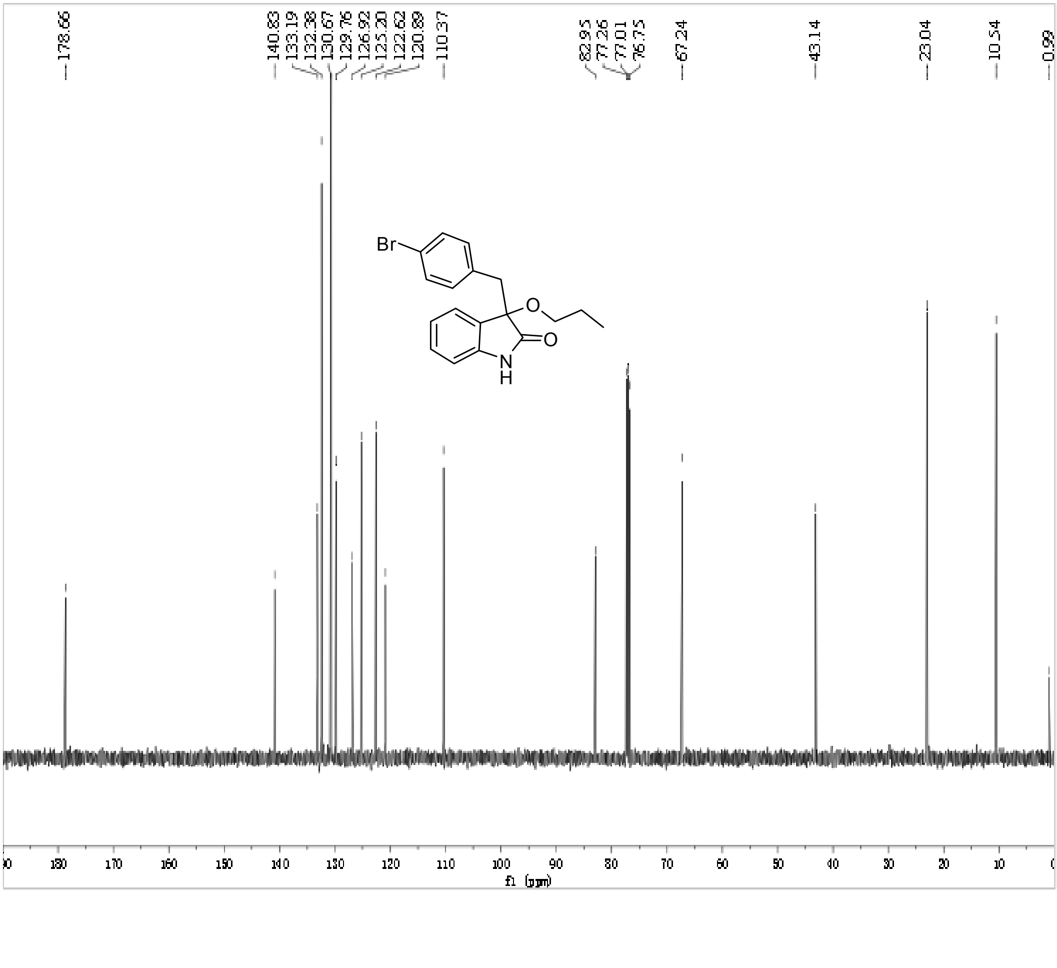

^{1}H and ^{13}C NMR of 3ec

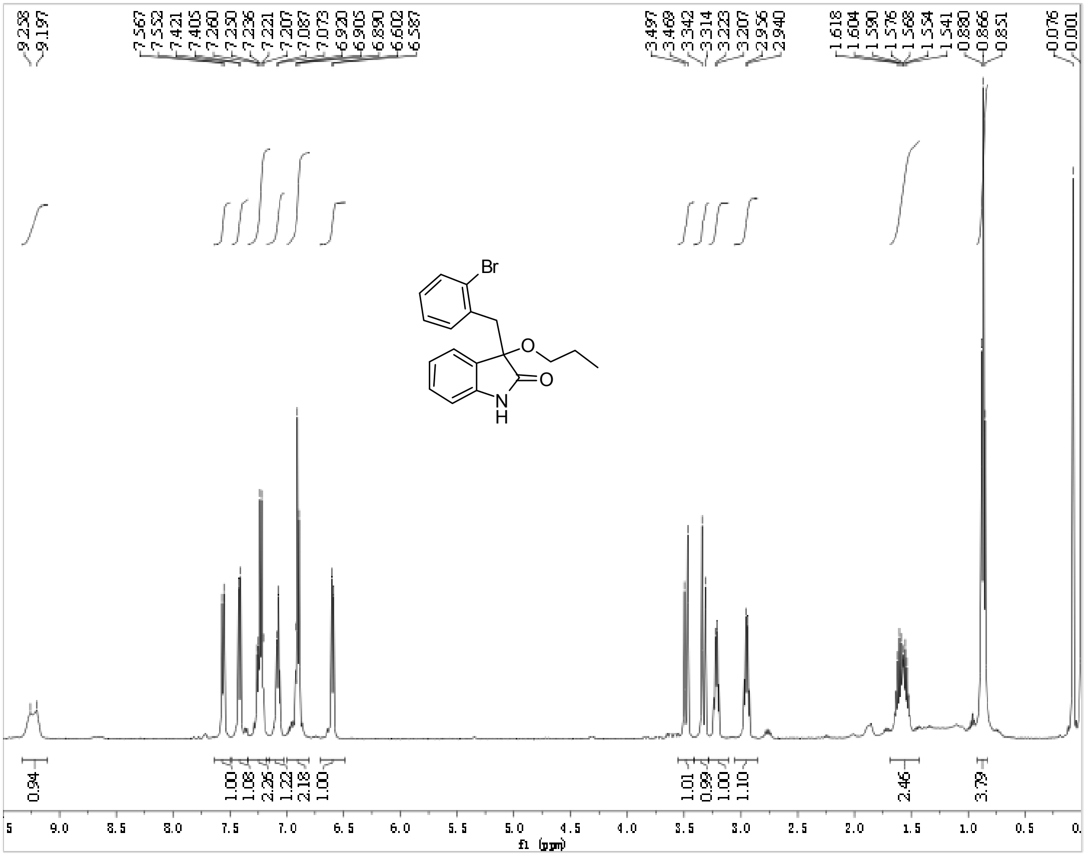

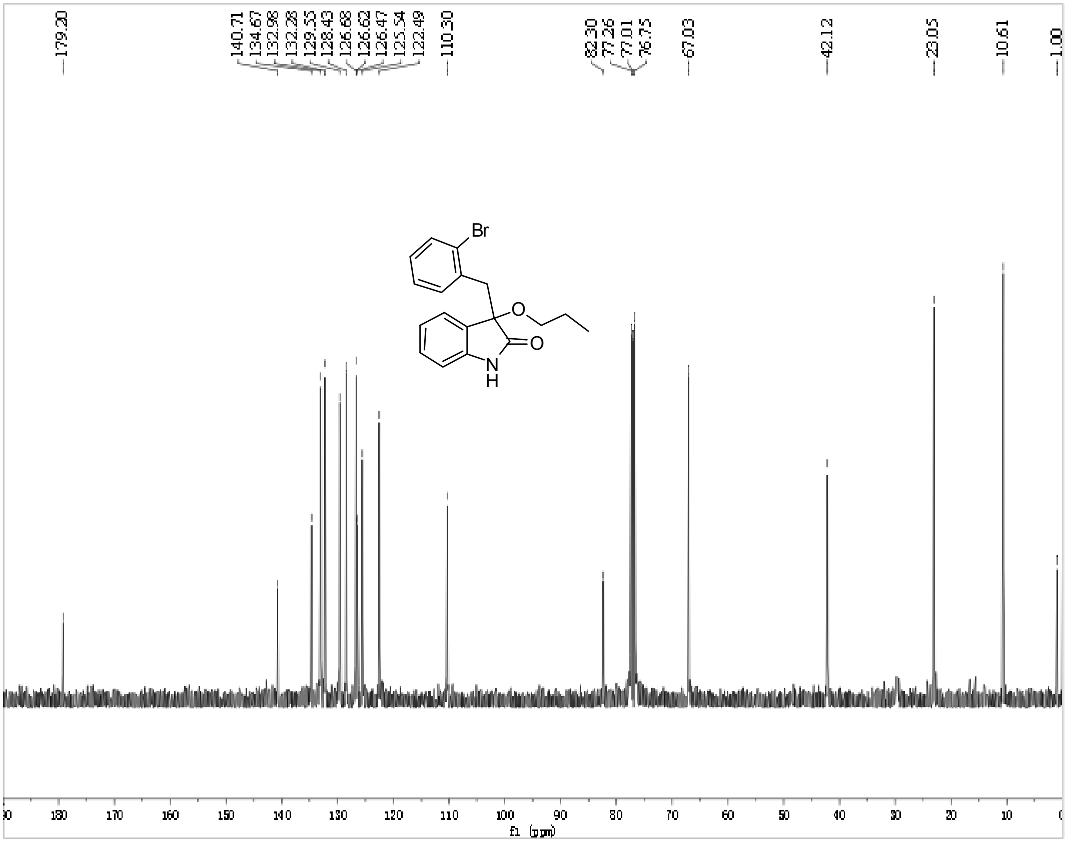

^{1}H and ^{13}C NMR of 3fc

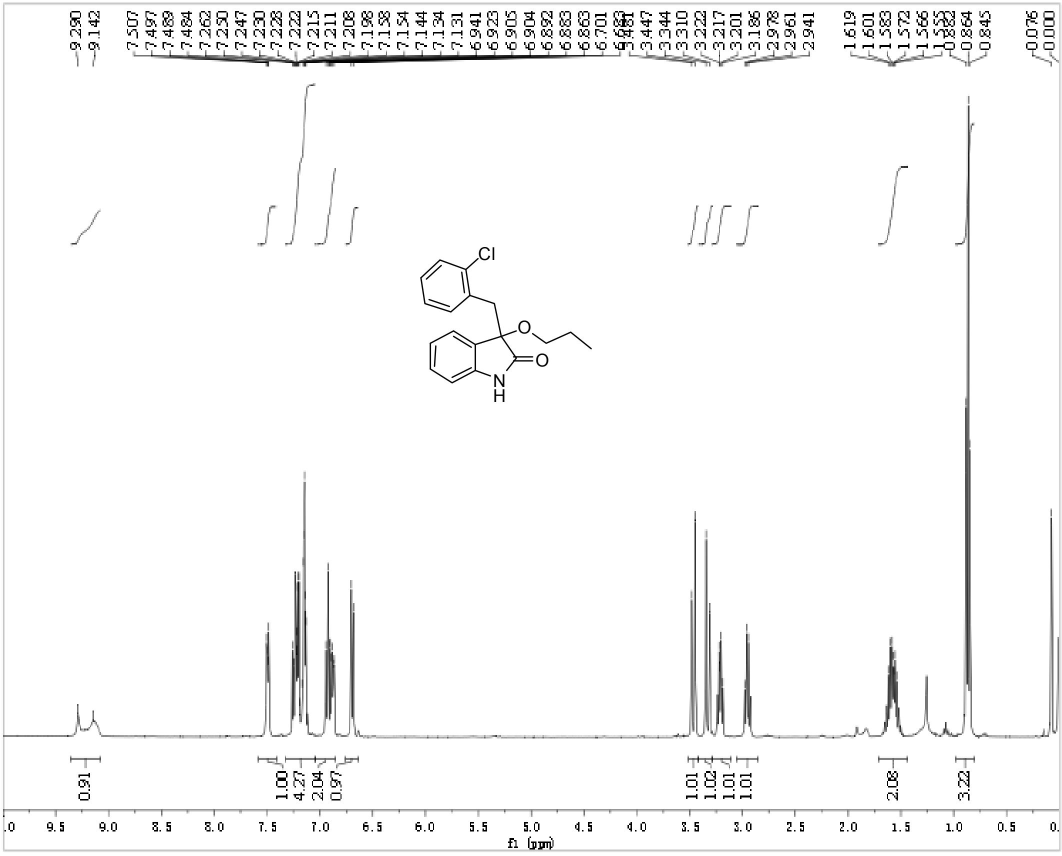

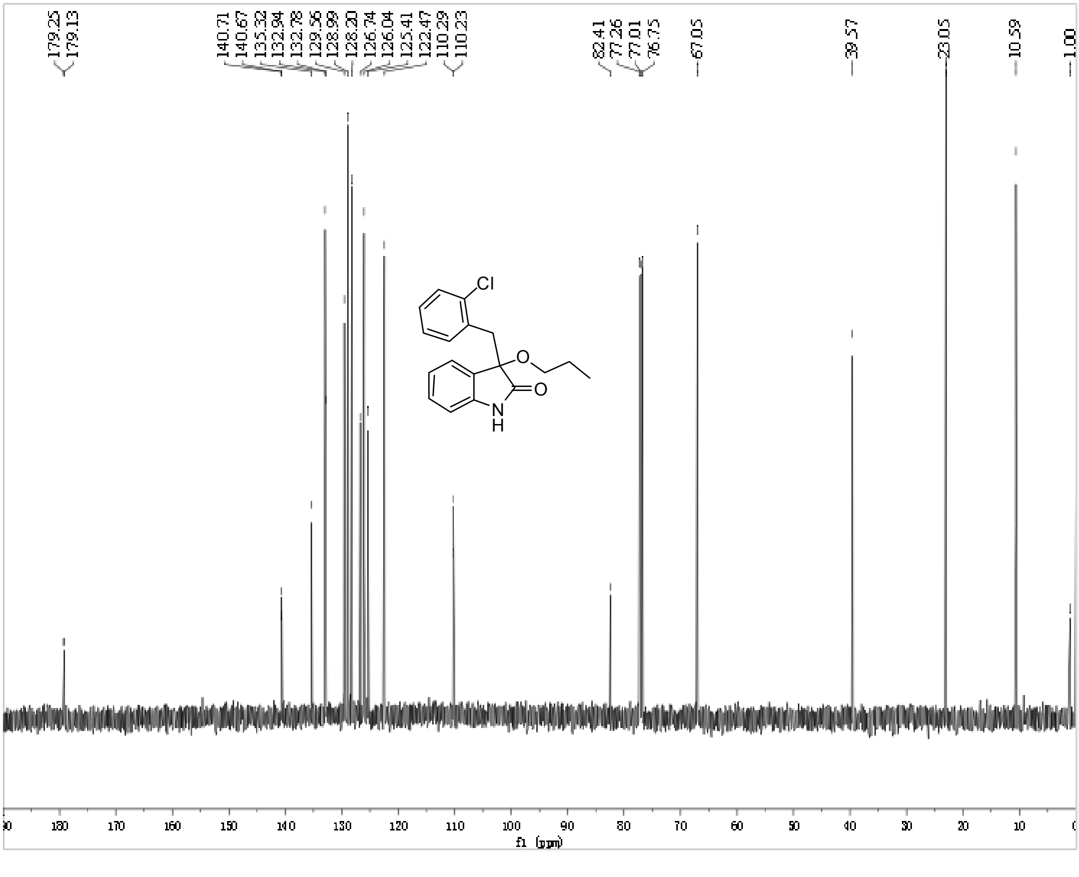

^{1}H and ^{13}C NMR of 3gc

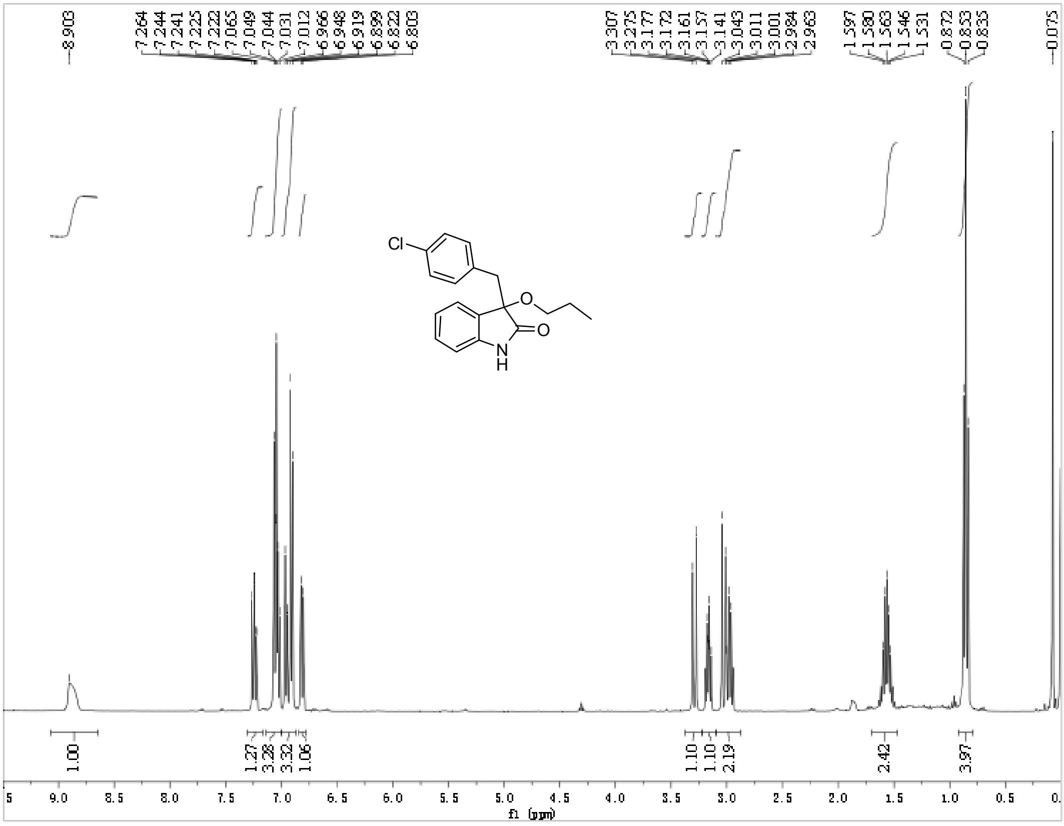

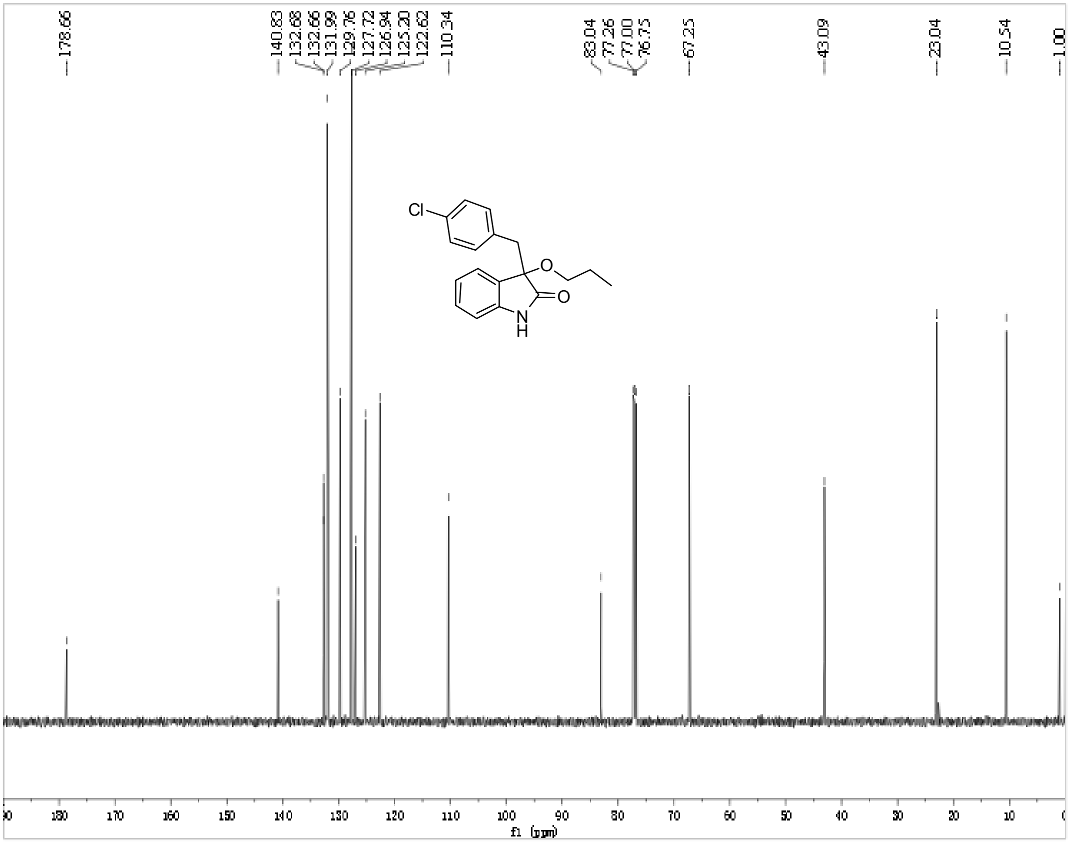

^{1}H and ^{13}C NMR of 3dd

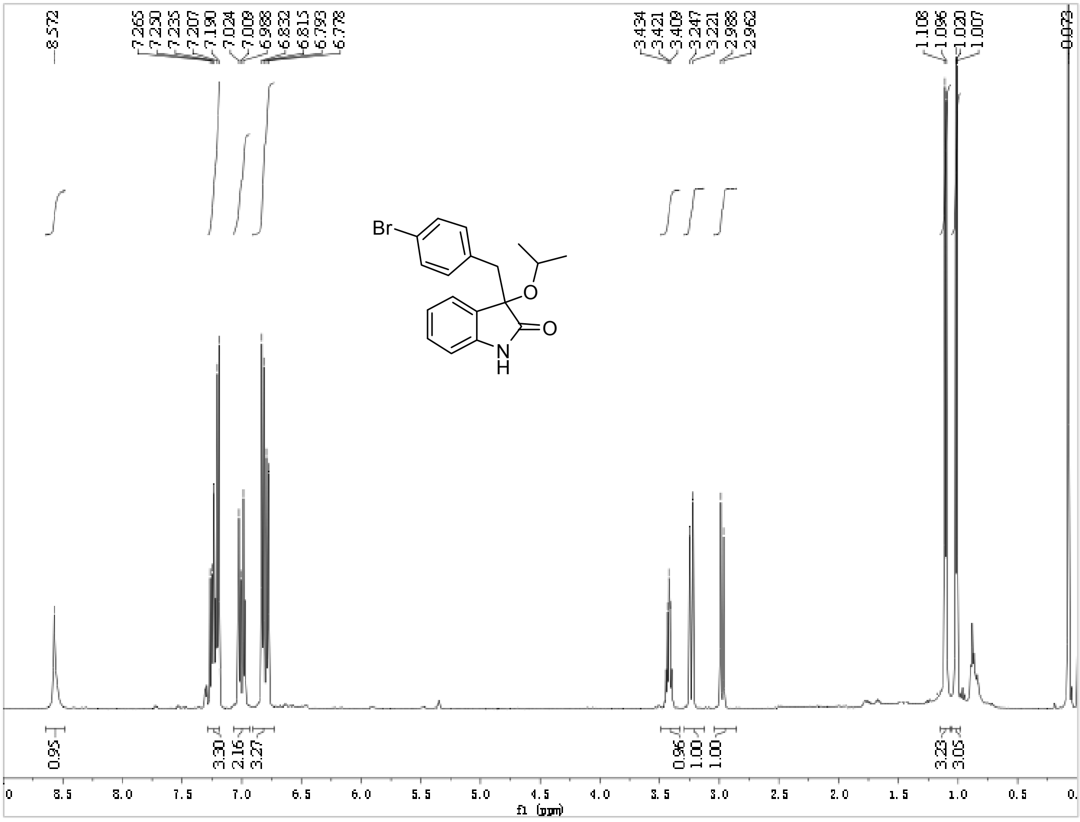

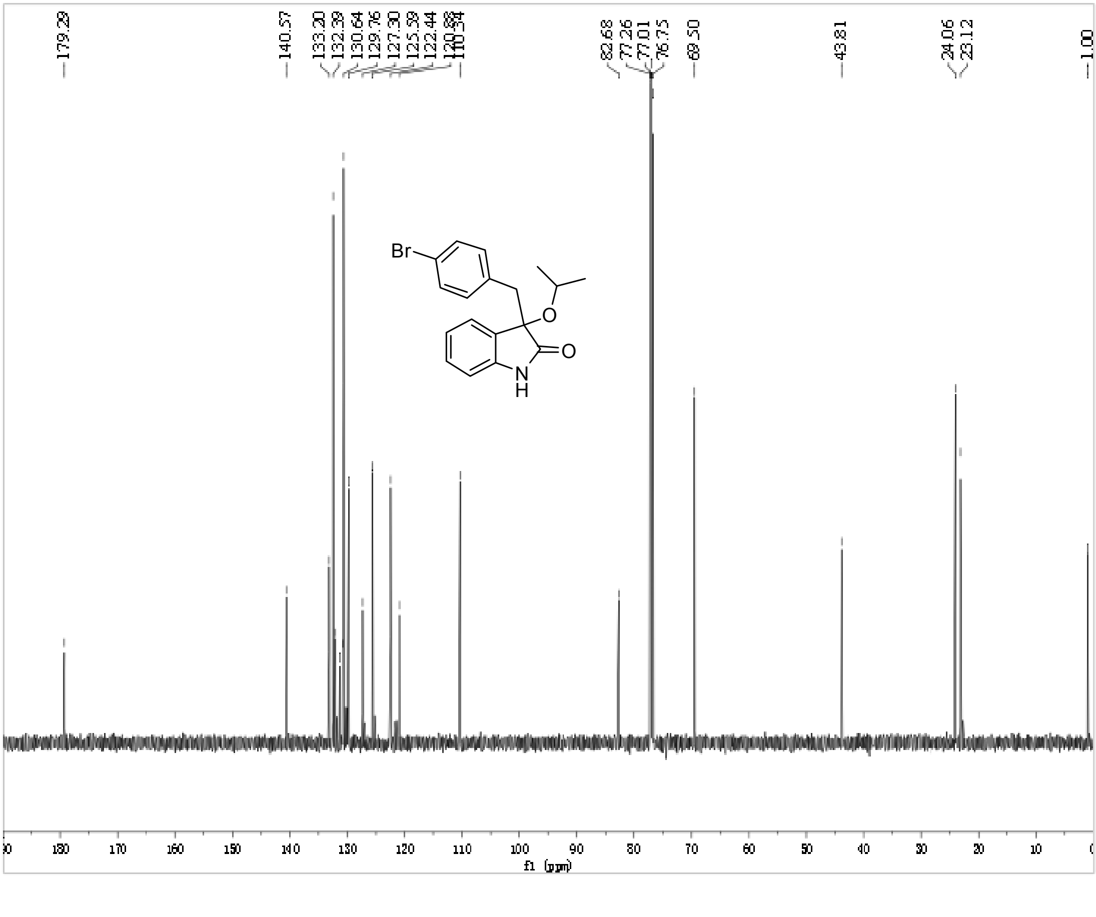

^{1}H and ^{13}C NMR of 3ed

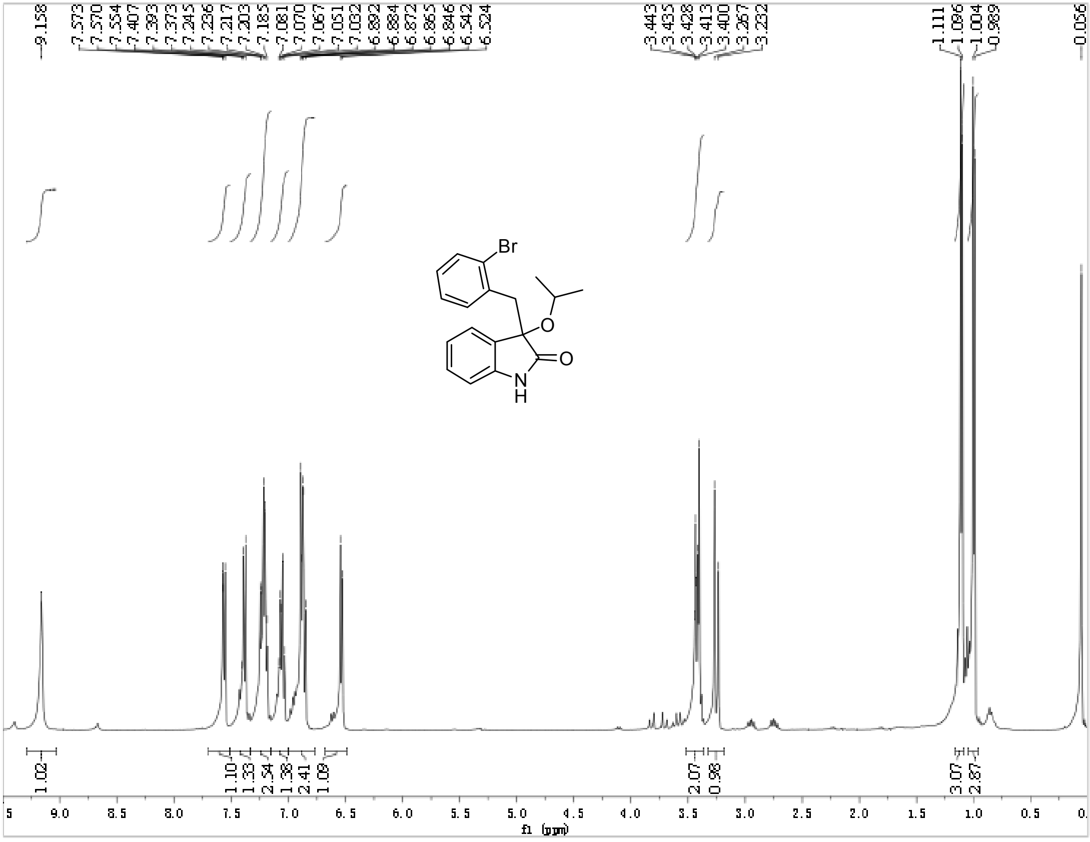

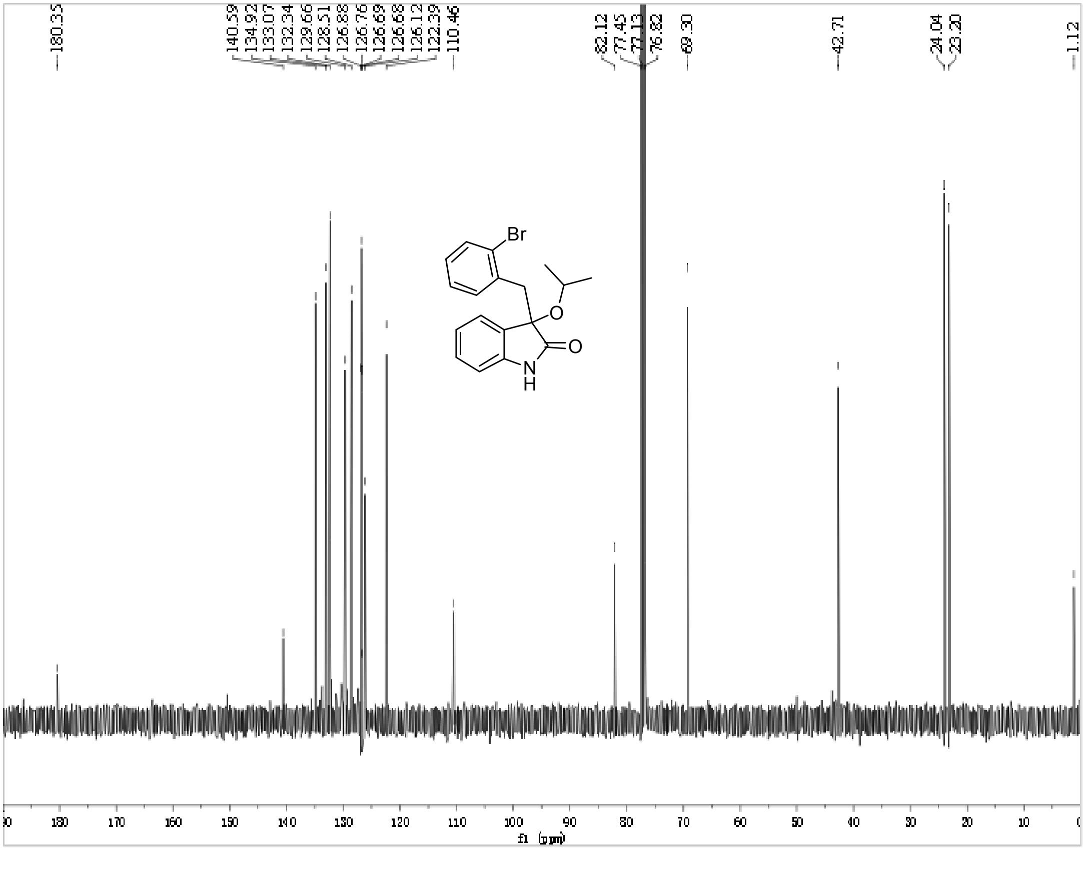

^{1}H and ^{13}C NMR of 3fd

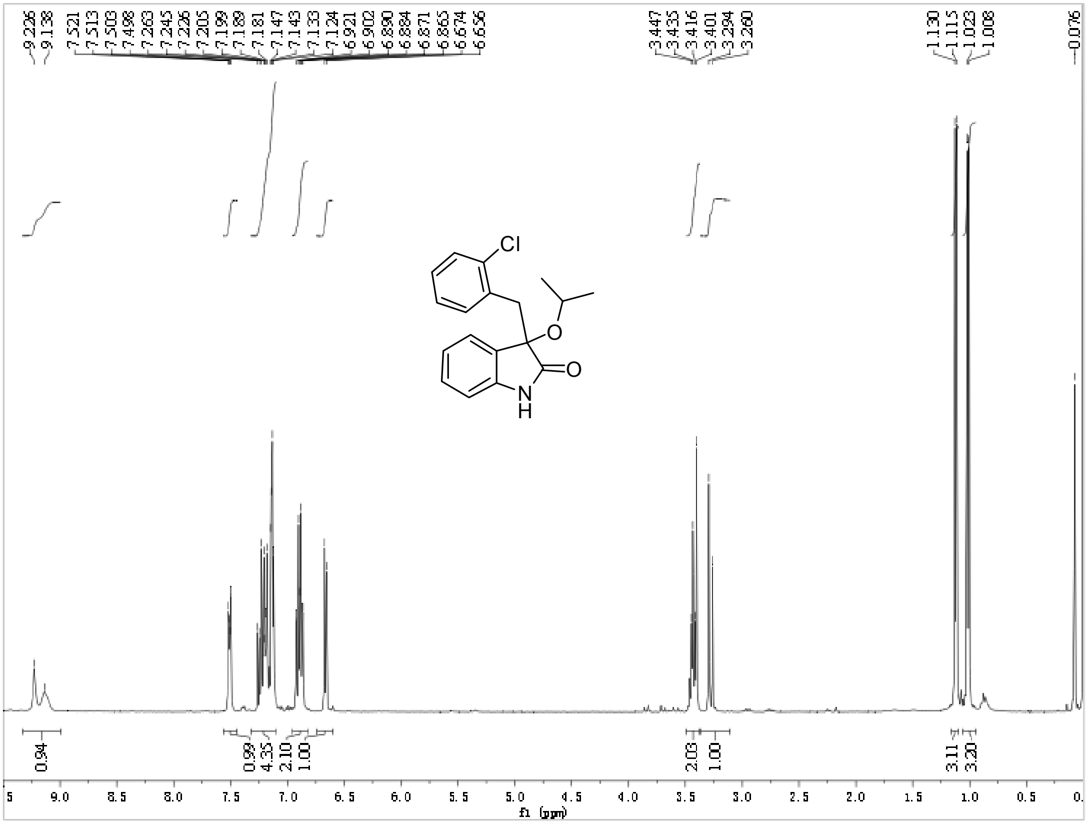

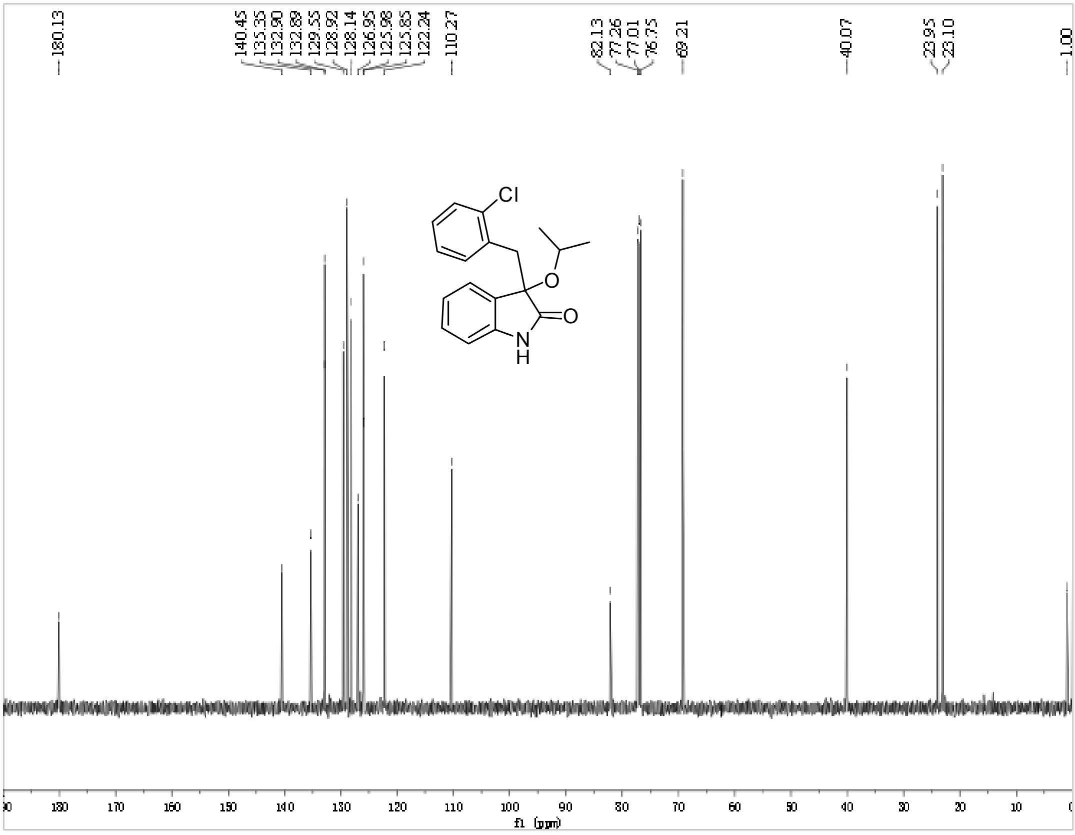

^{1}H and ^{13}C NMR of 3gd

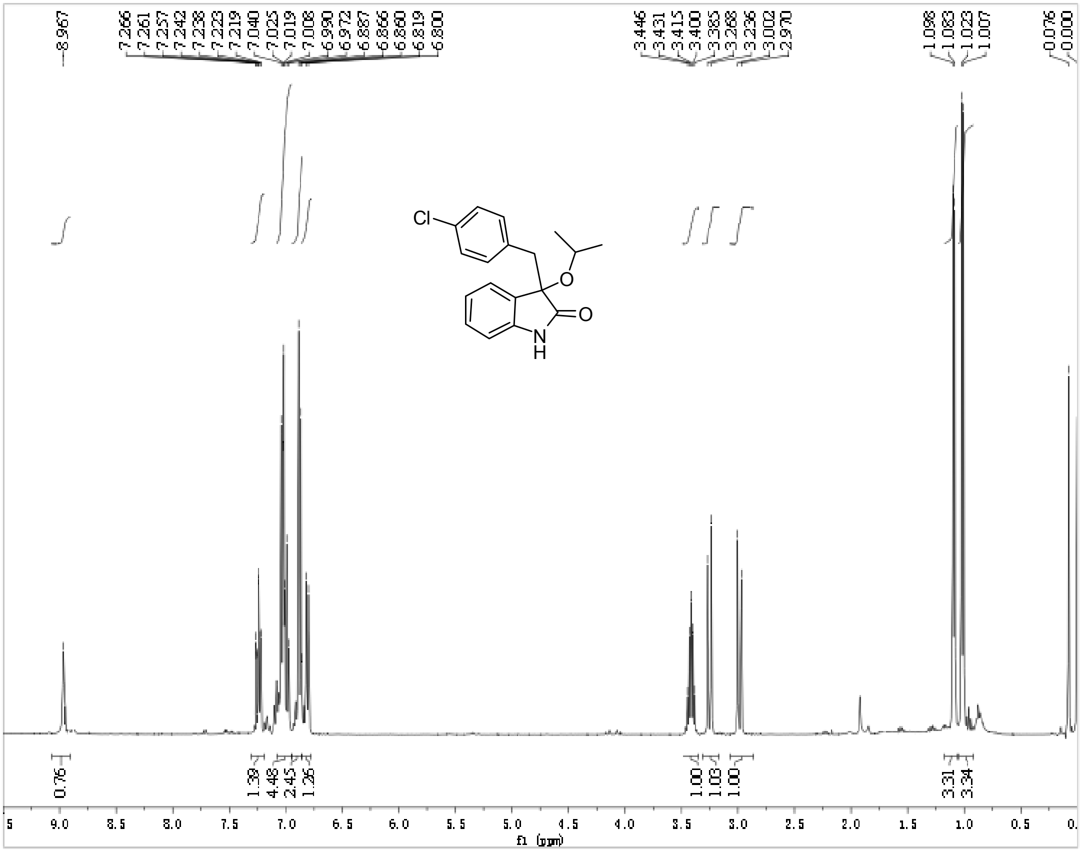

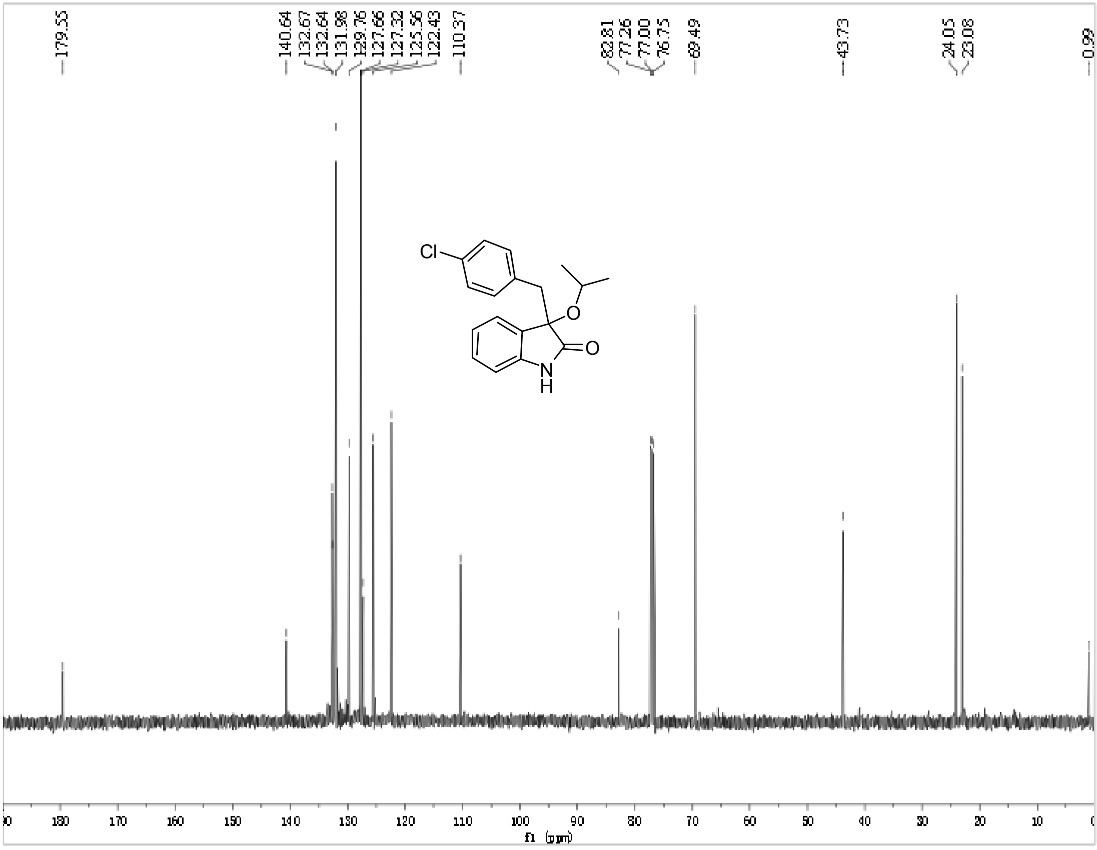

^{1}H and ^{13}C NMR of 3ae

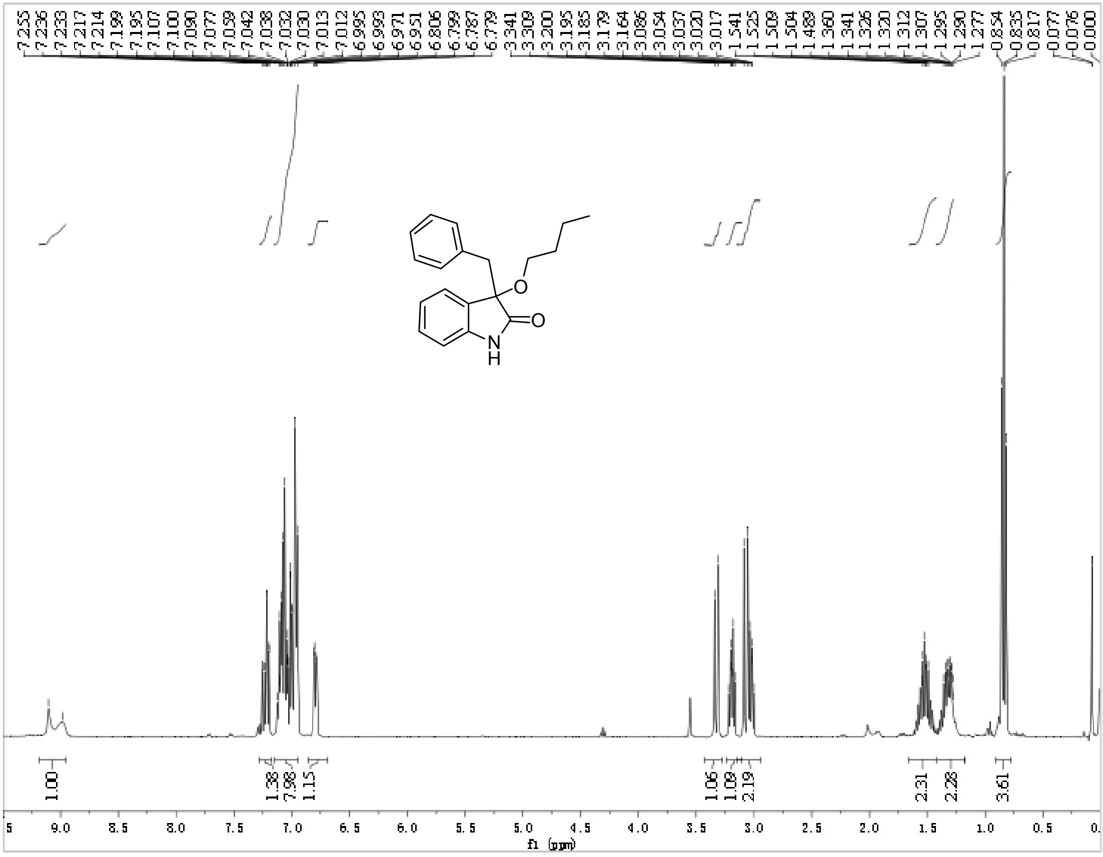

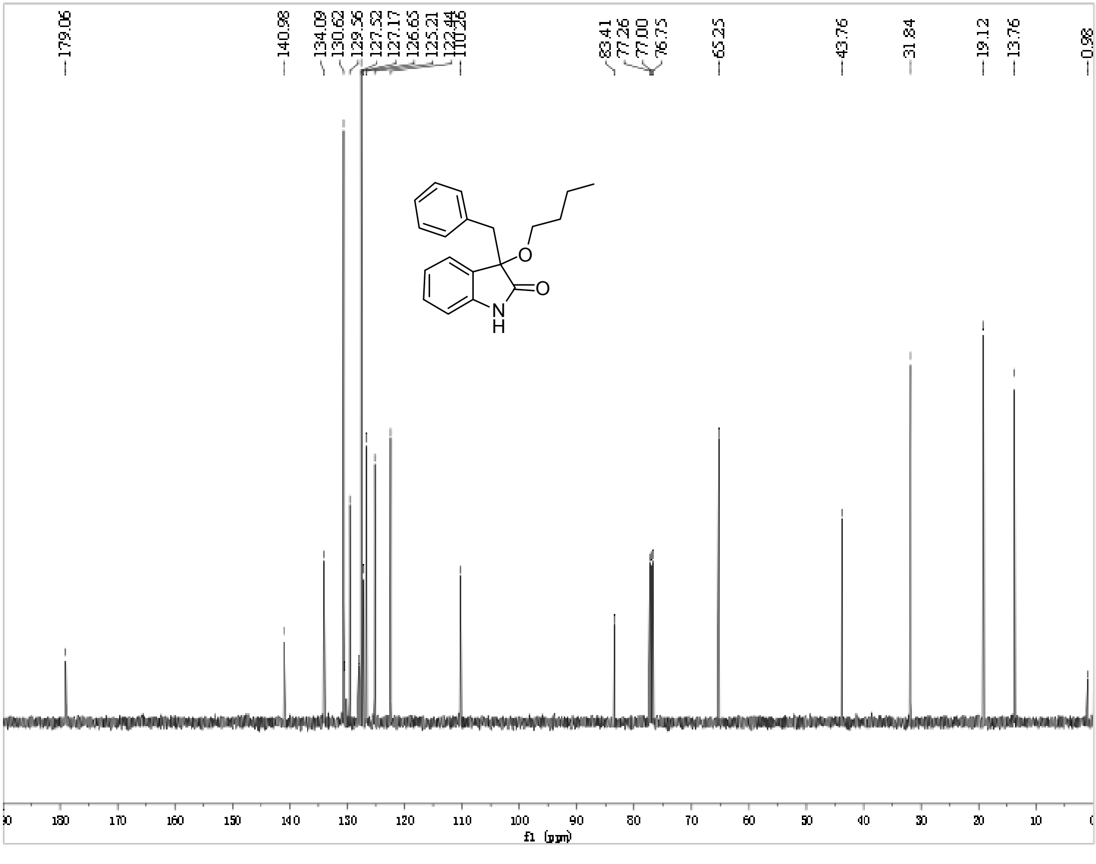

^{1}H and ^{13}C NMR of 3be

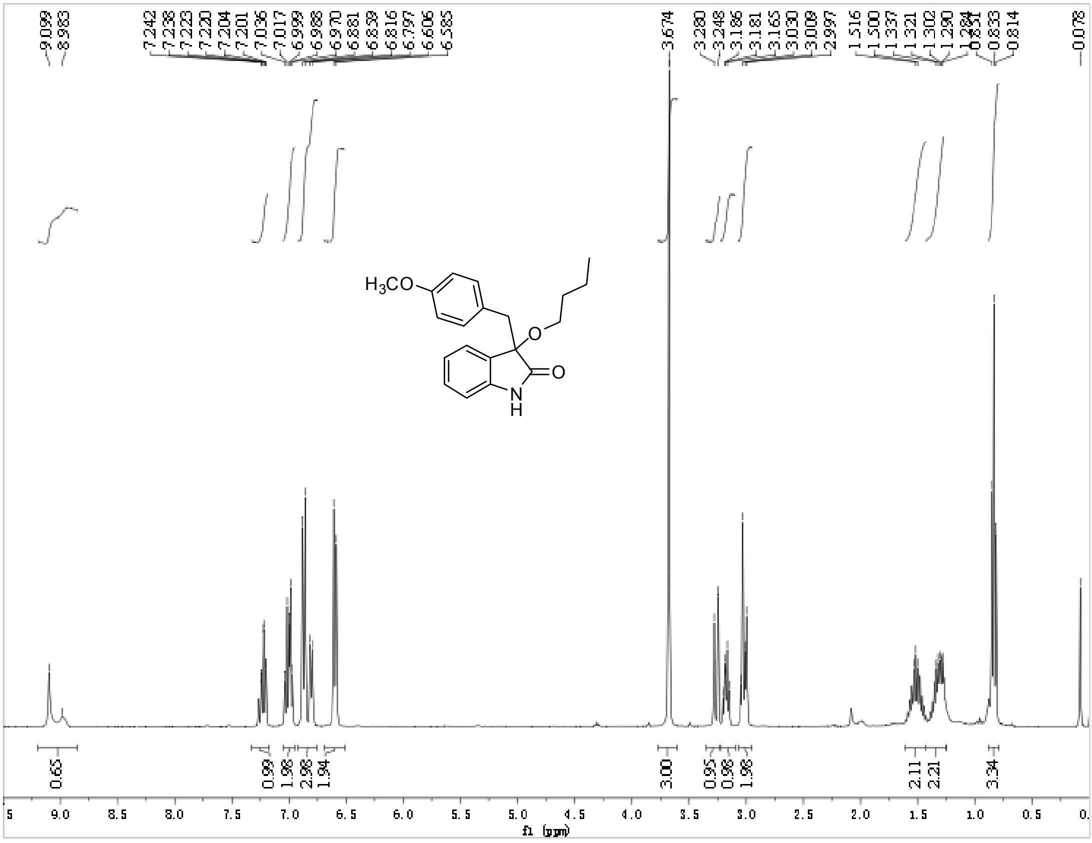

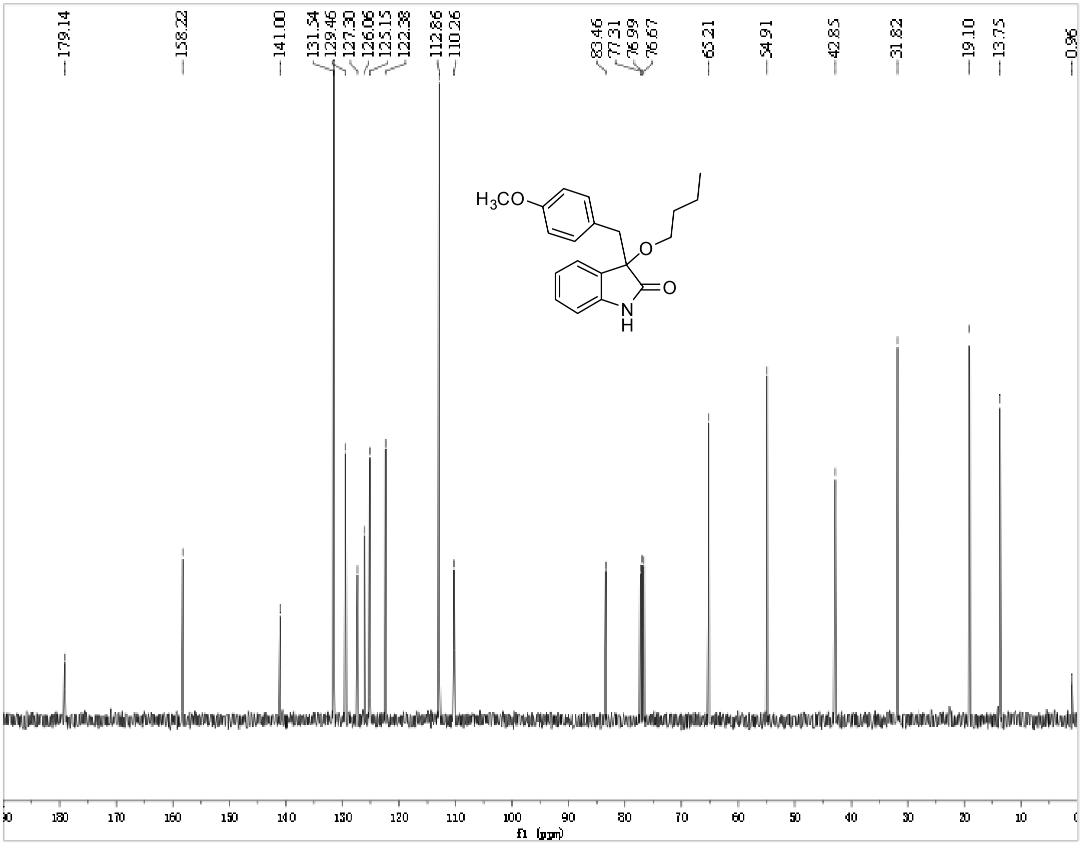

^{1}H and ^{13}C NMR of 3ce

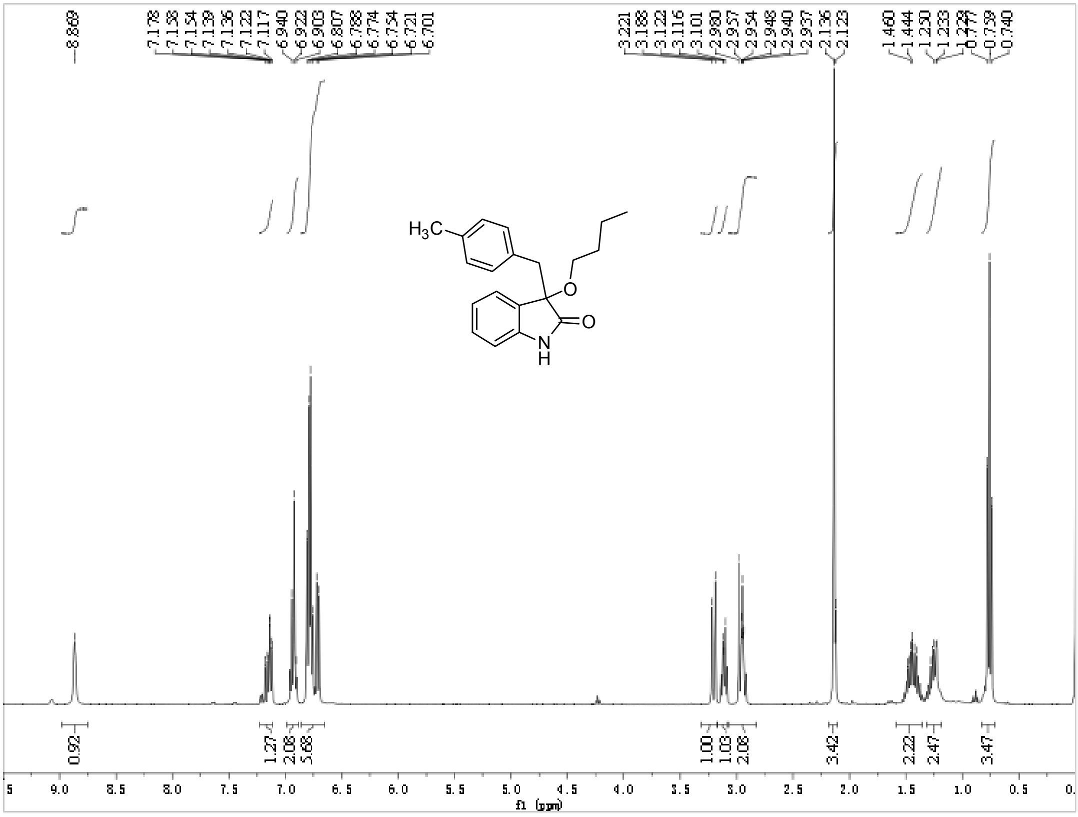

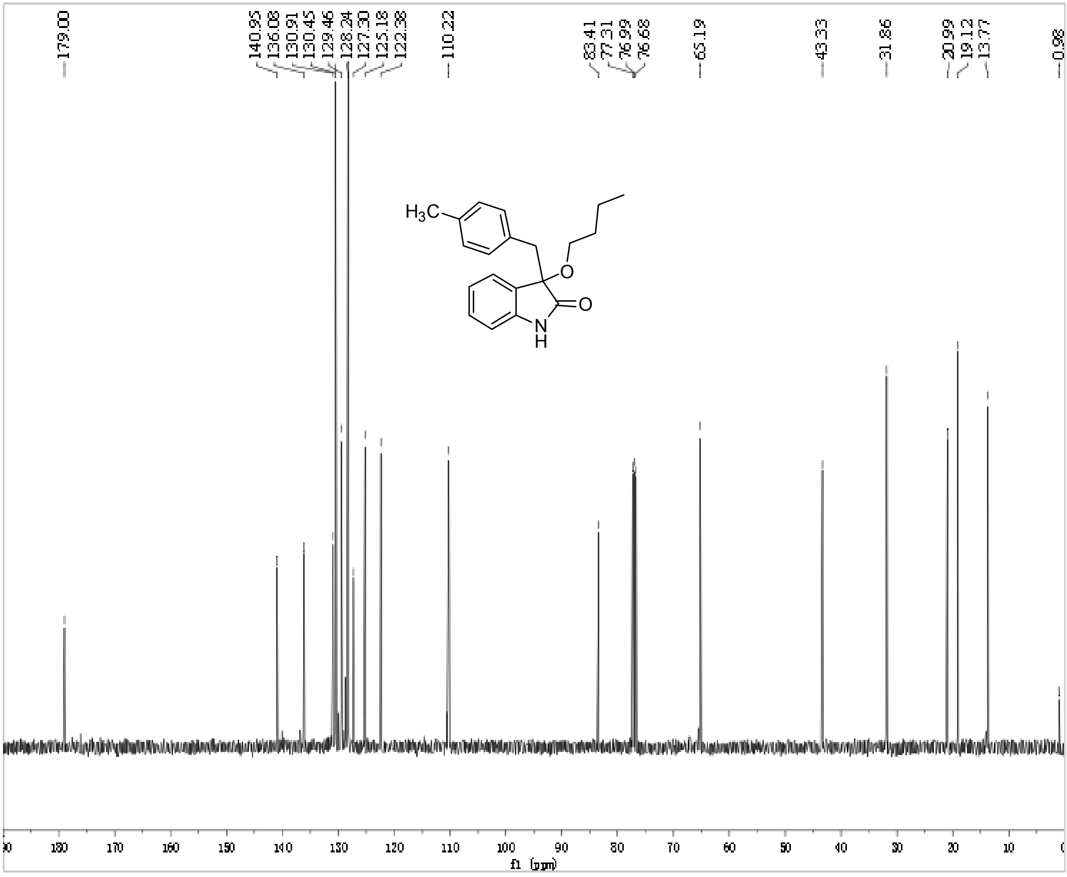

^{1}H and ^{13}C NMR of 3de

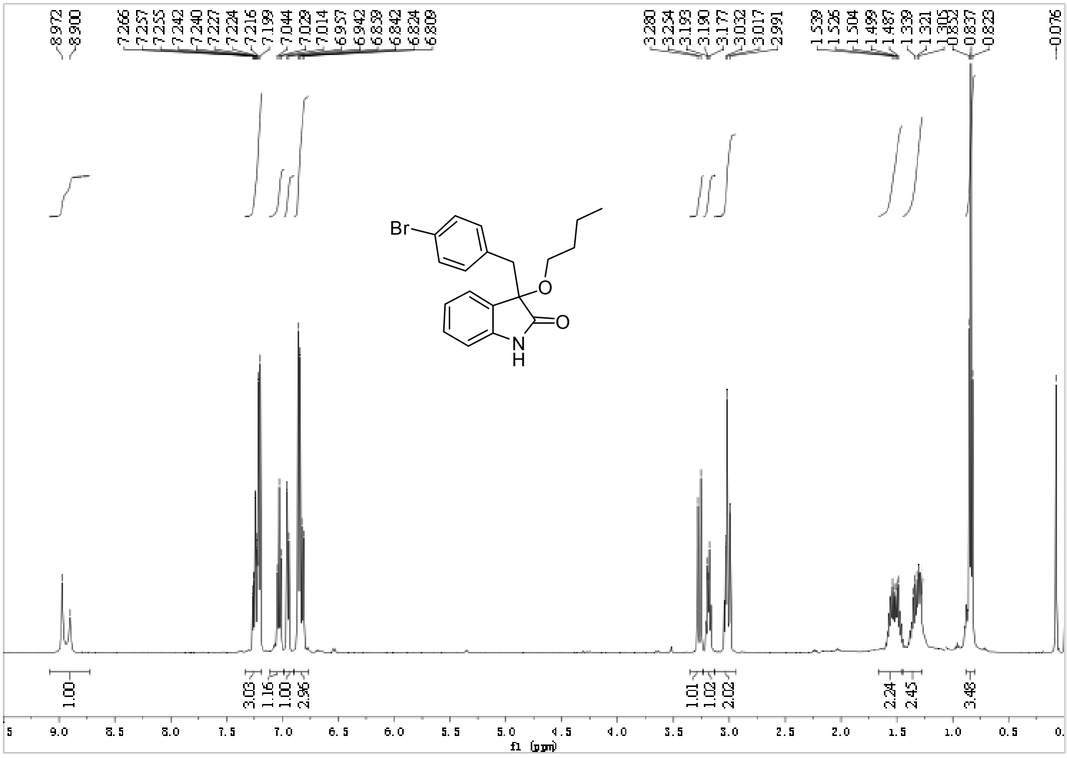

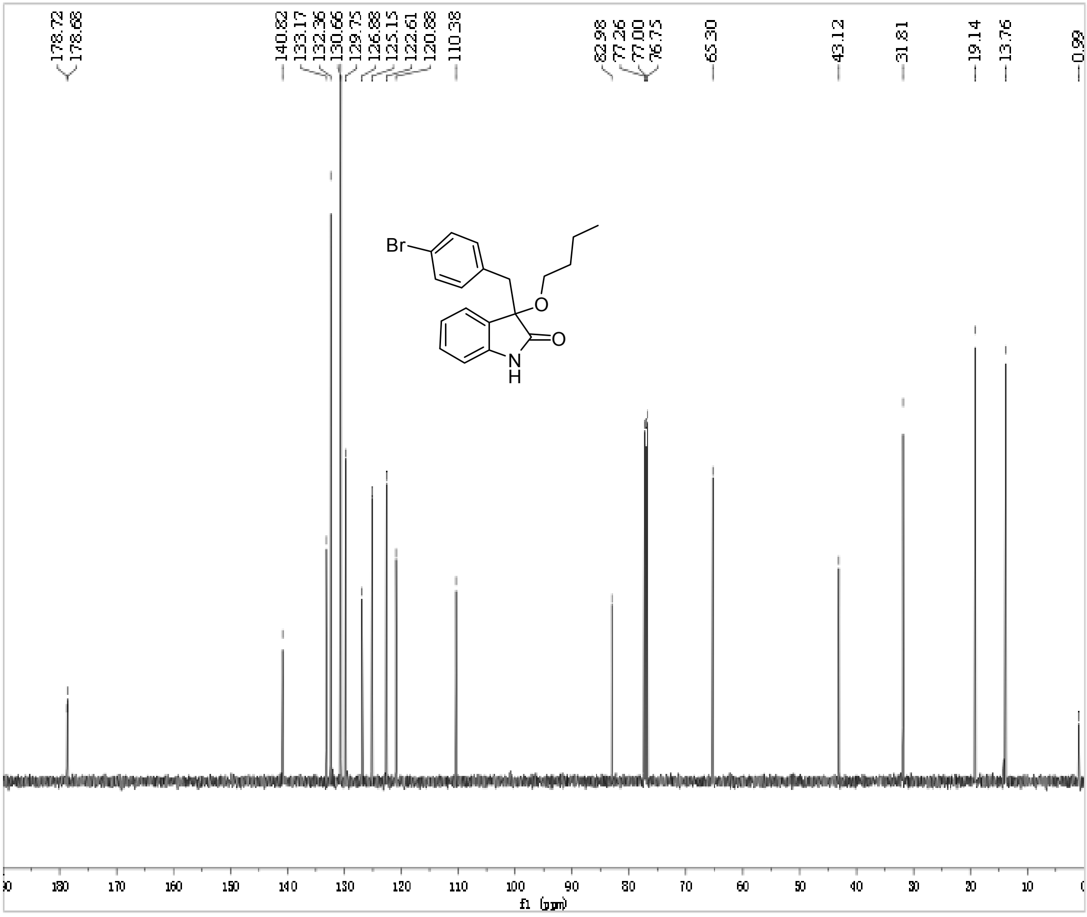

^{1}H and ^{13}C NMR of 3ee

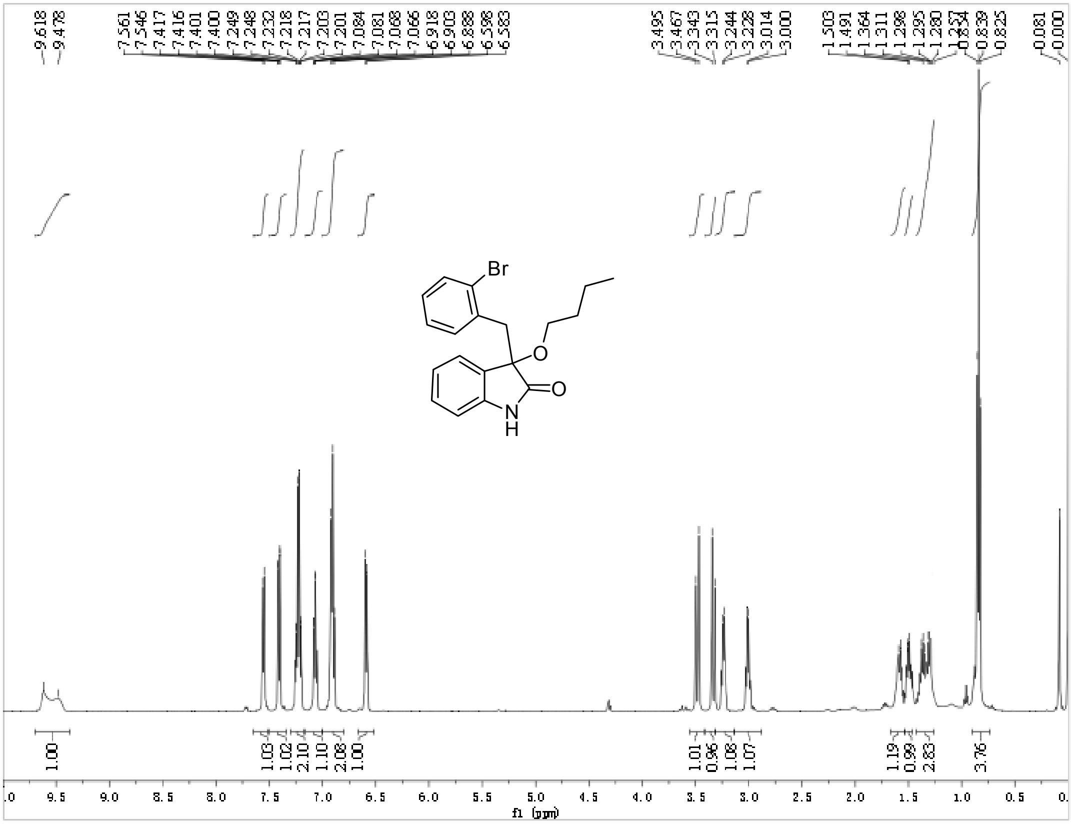

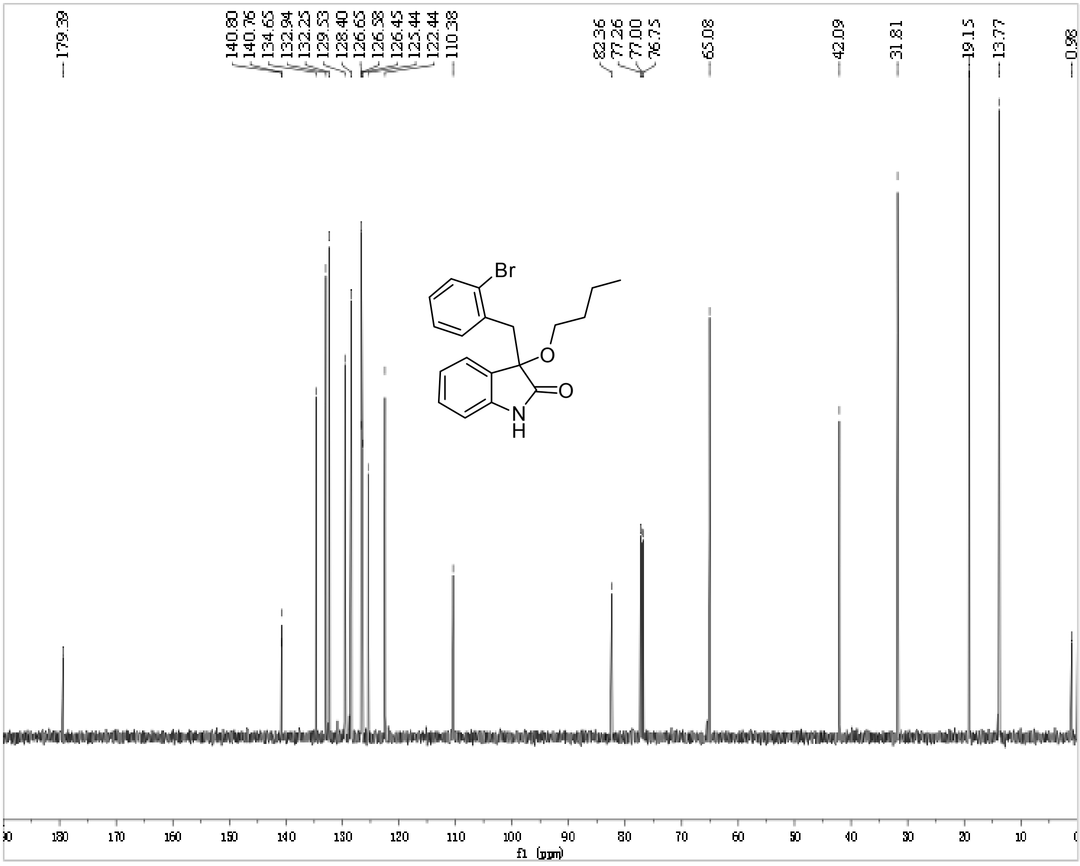

^{1}H and ^{13}C NMR of 3fe

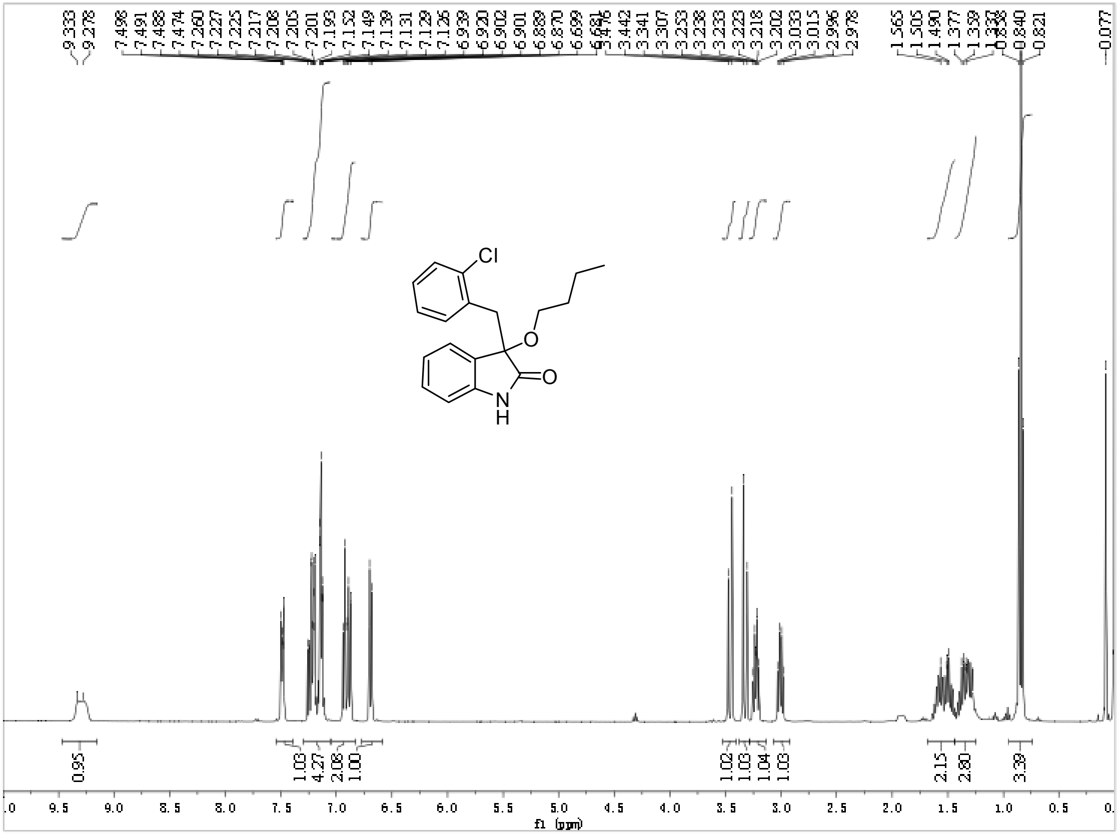

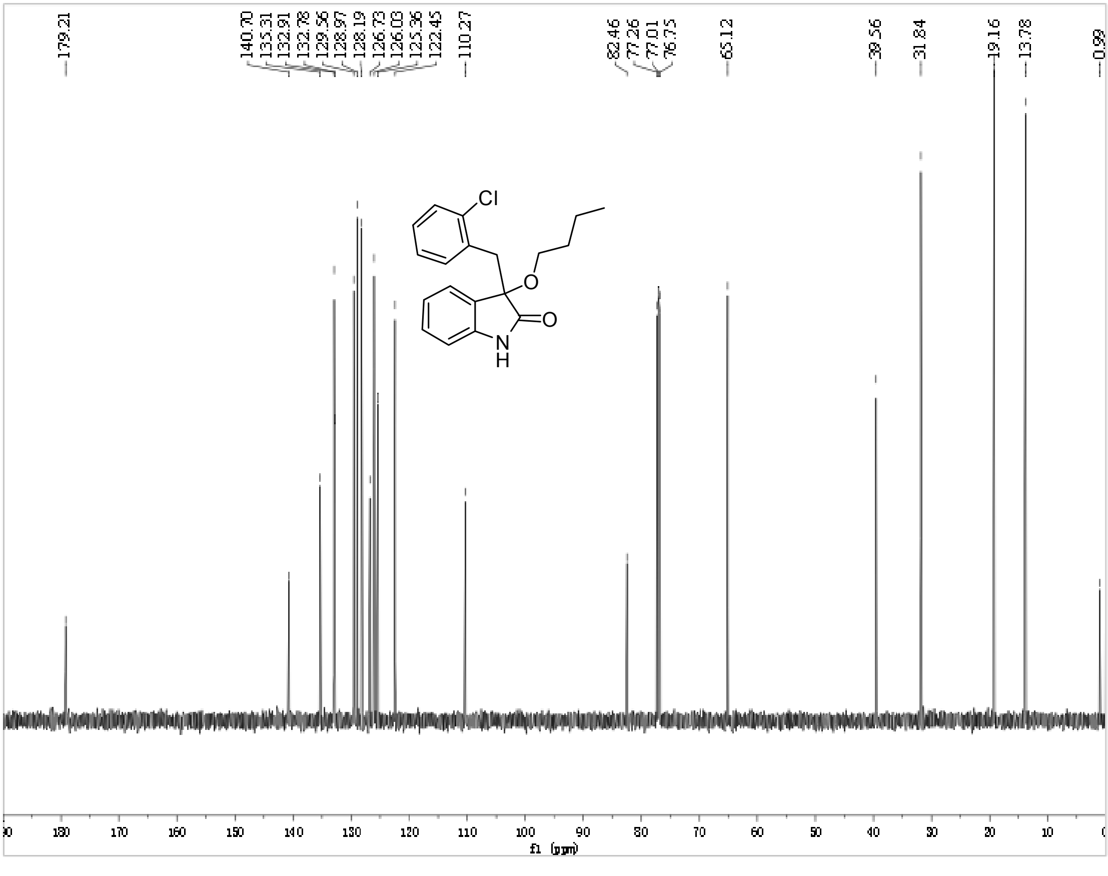

^{1}H and ^{13}C NMR of 3ge

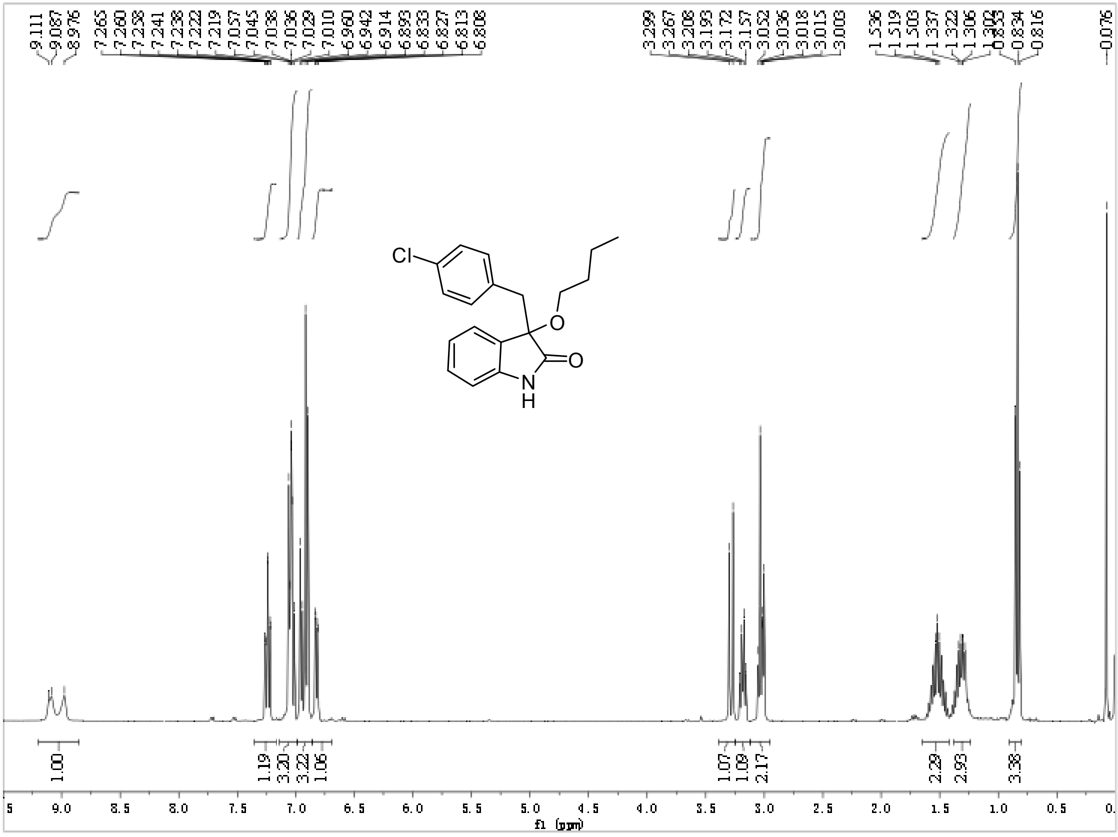

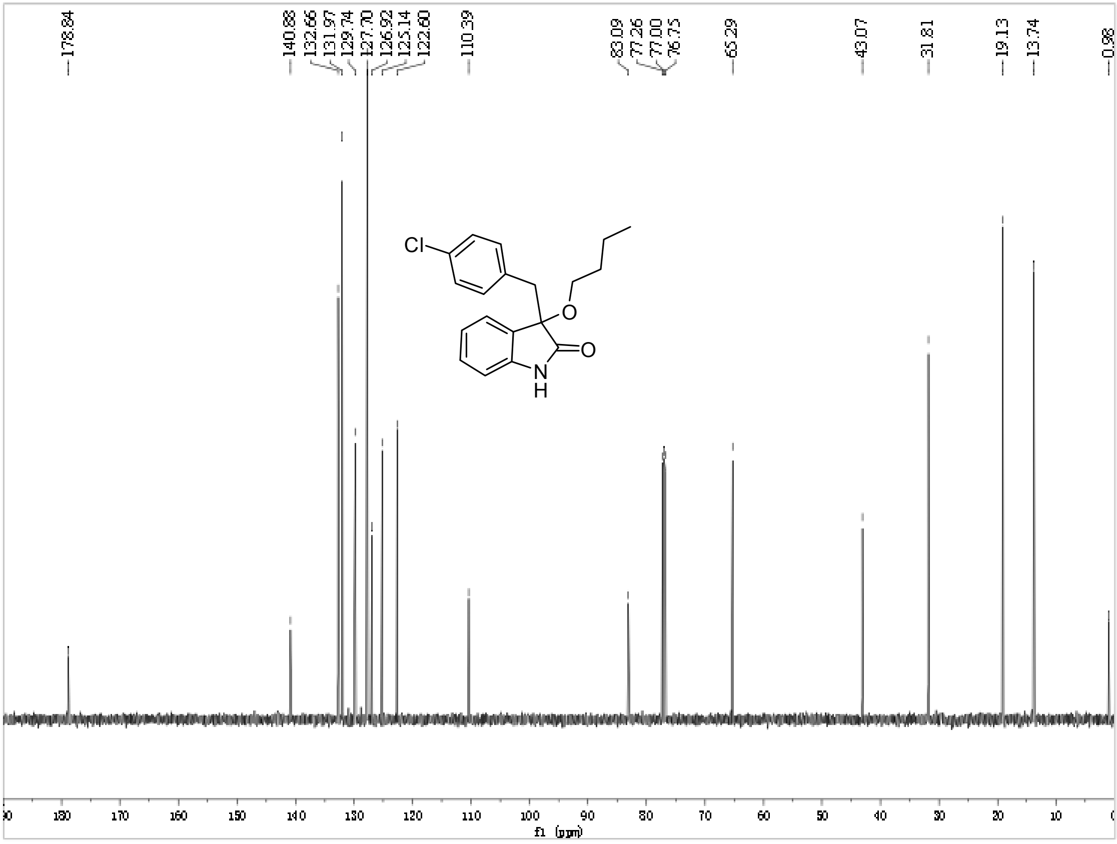

^{1}H and ^{13}C NMR of 3he

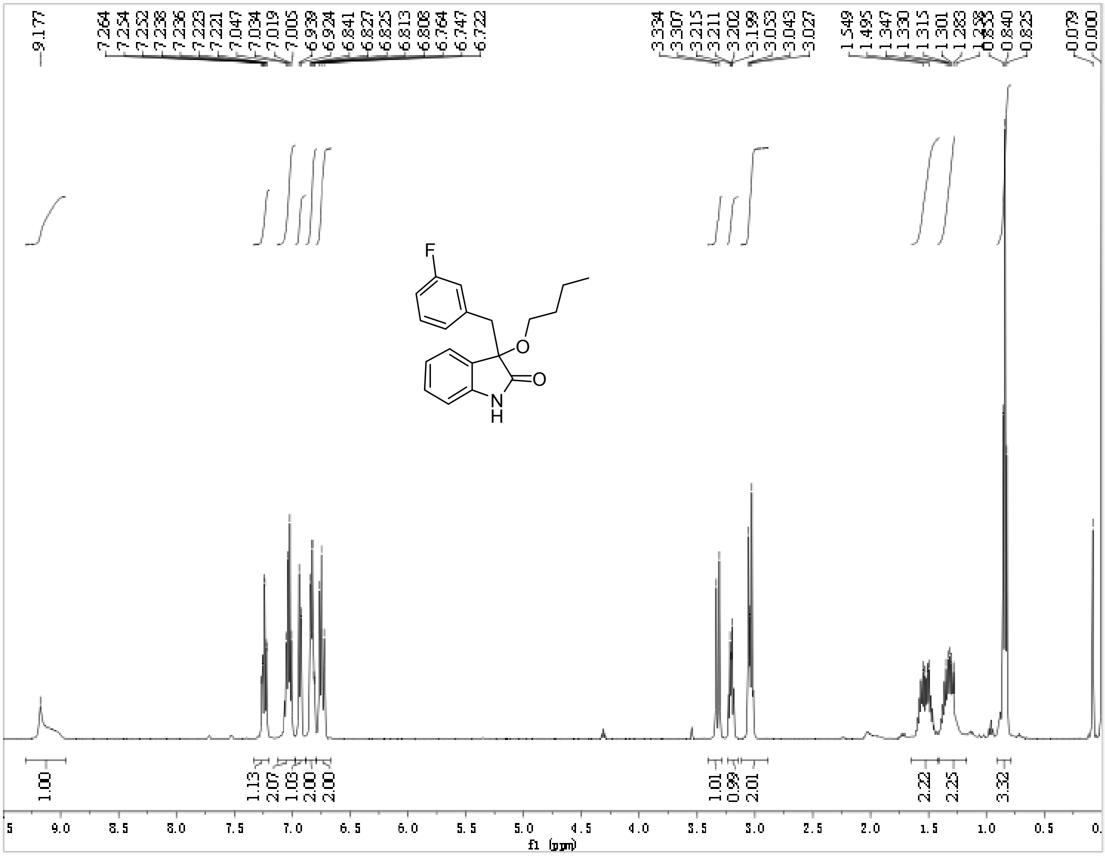

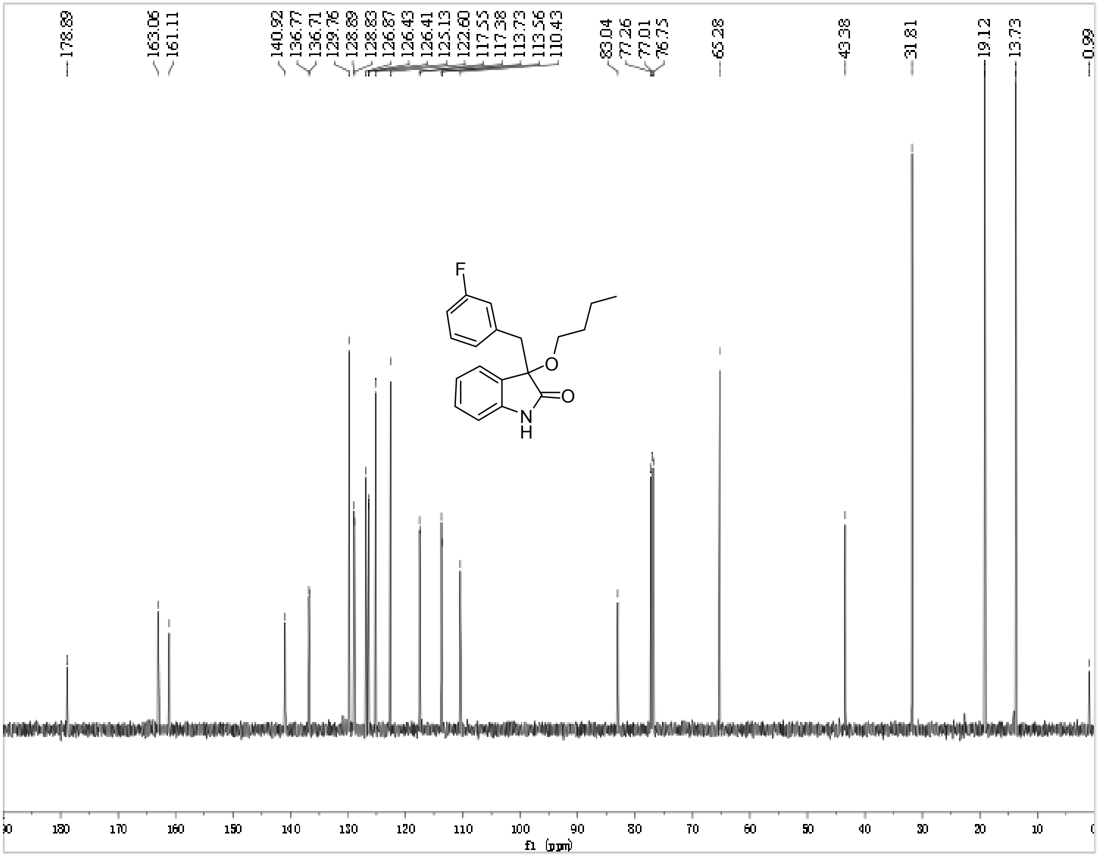

^{1}H and ^{13}C NMR of 3af

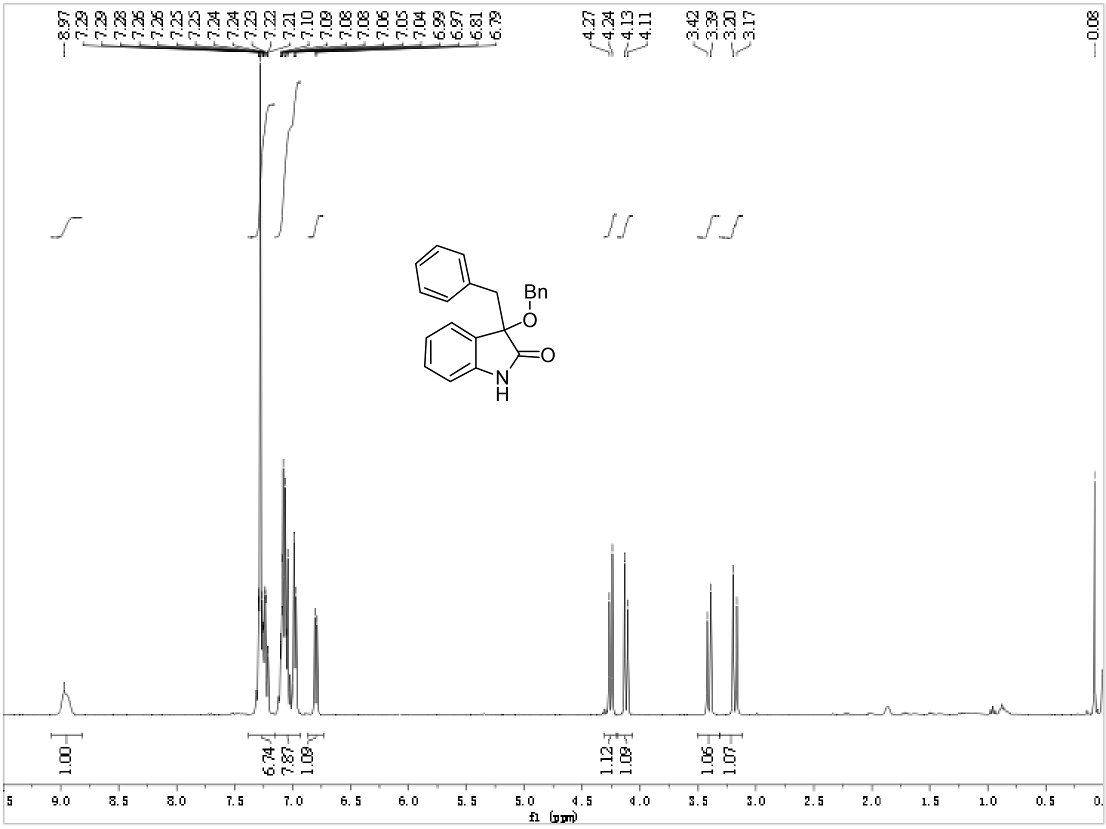

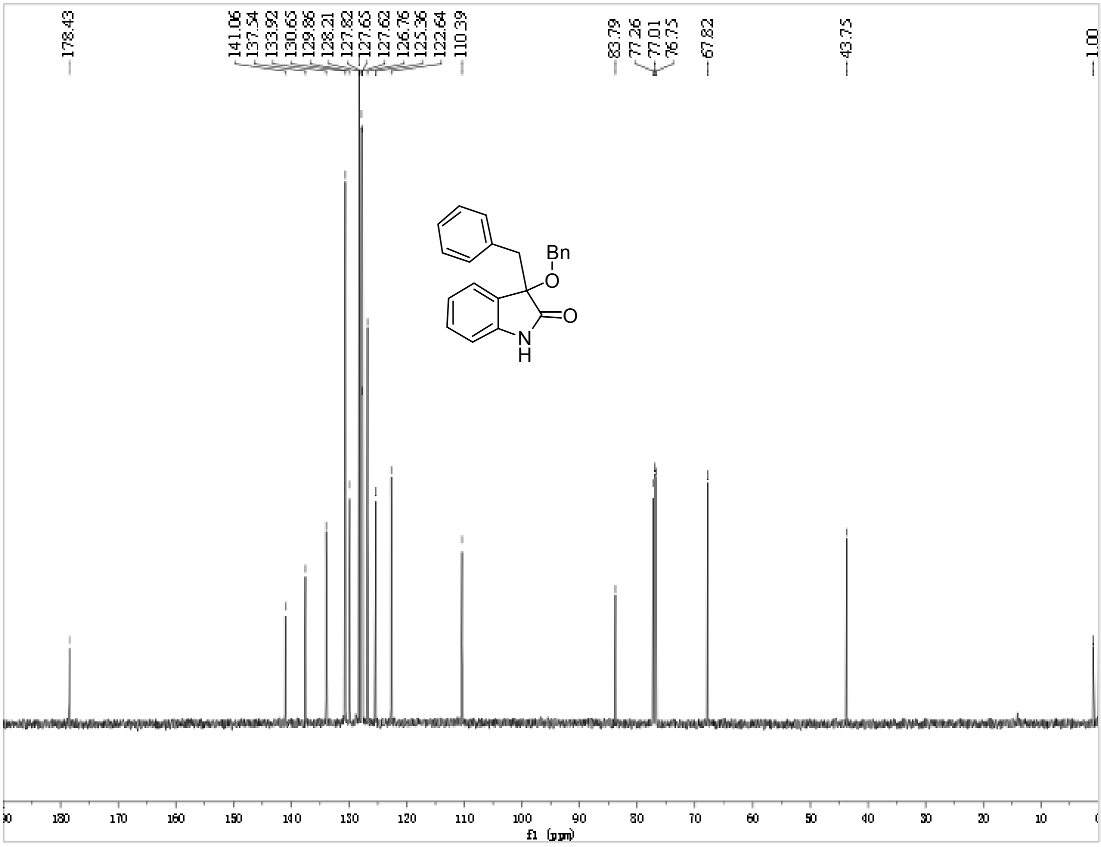

^{1}H and ^{13}C NMR of 3bf

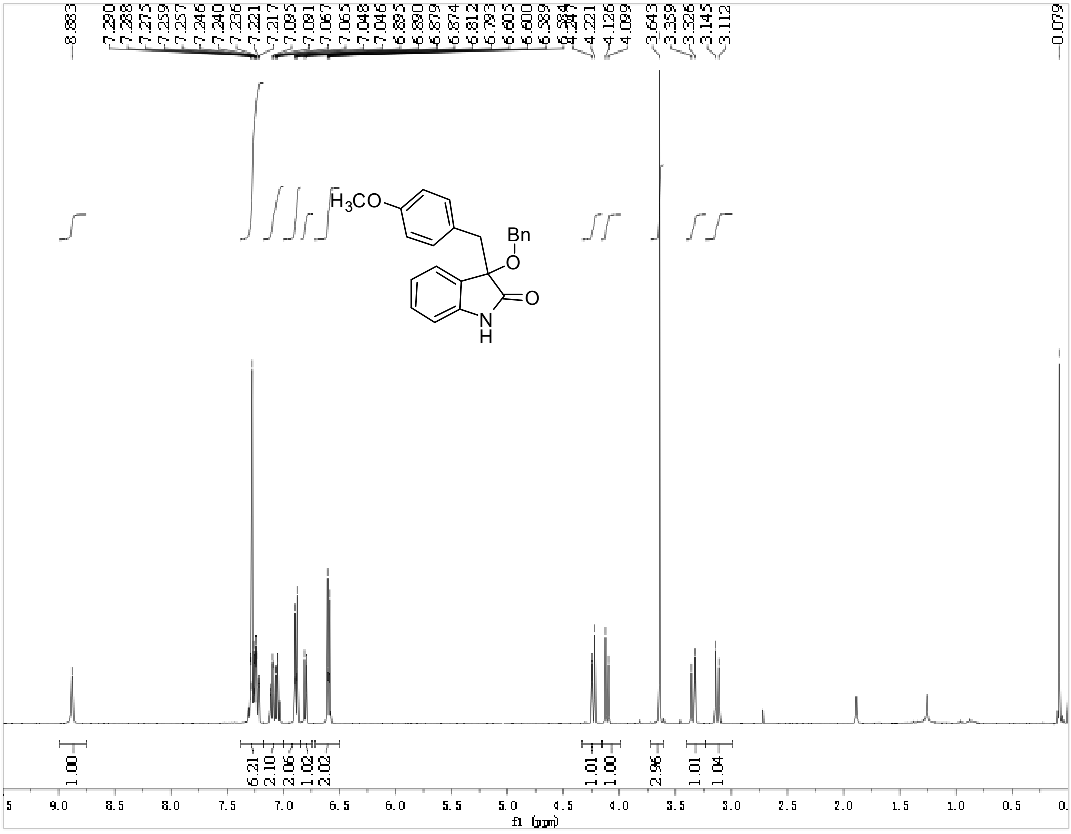

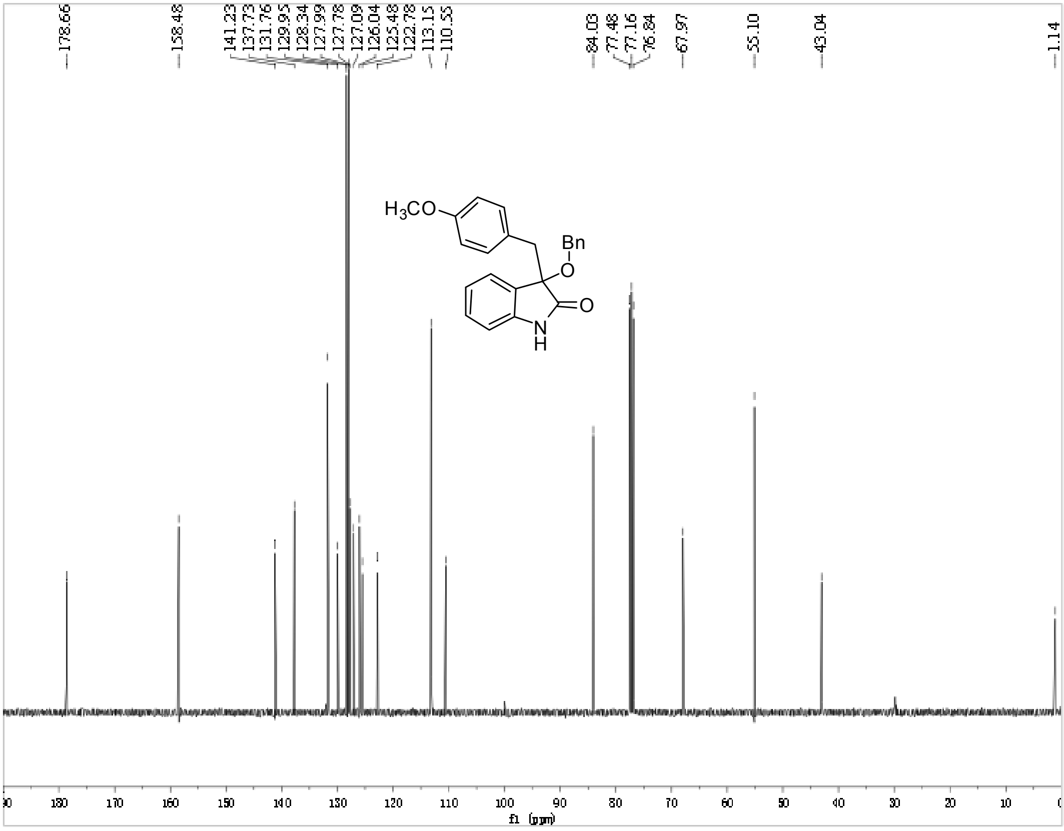

^{1}H and ^{13}C NMR of 3cf

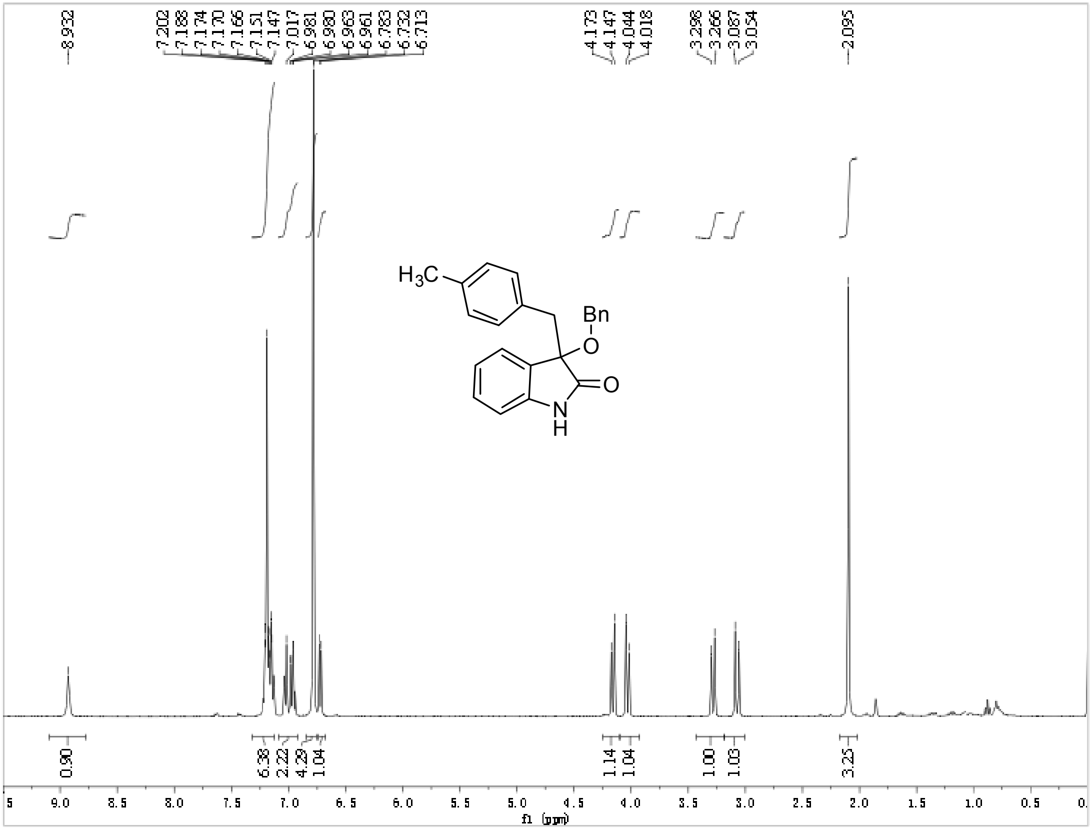

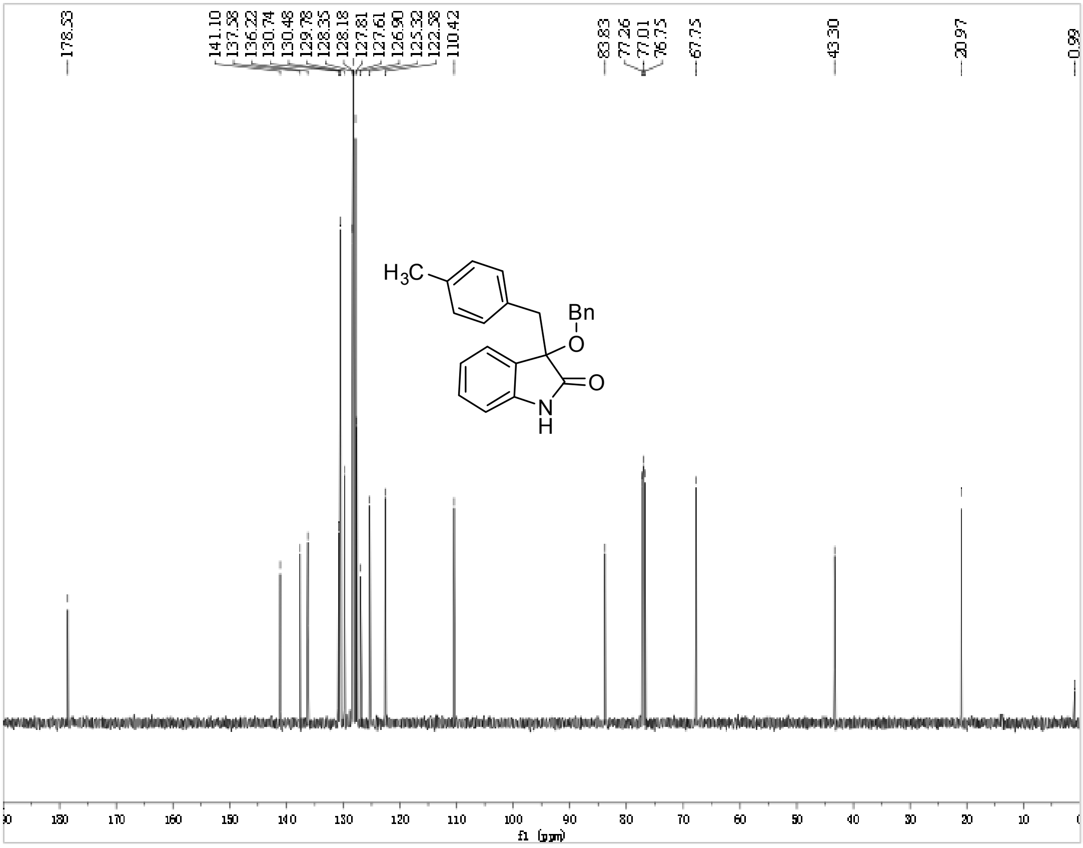

^{1}H and ^{13}C NMR of 3df

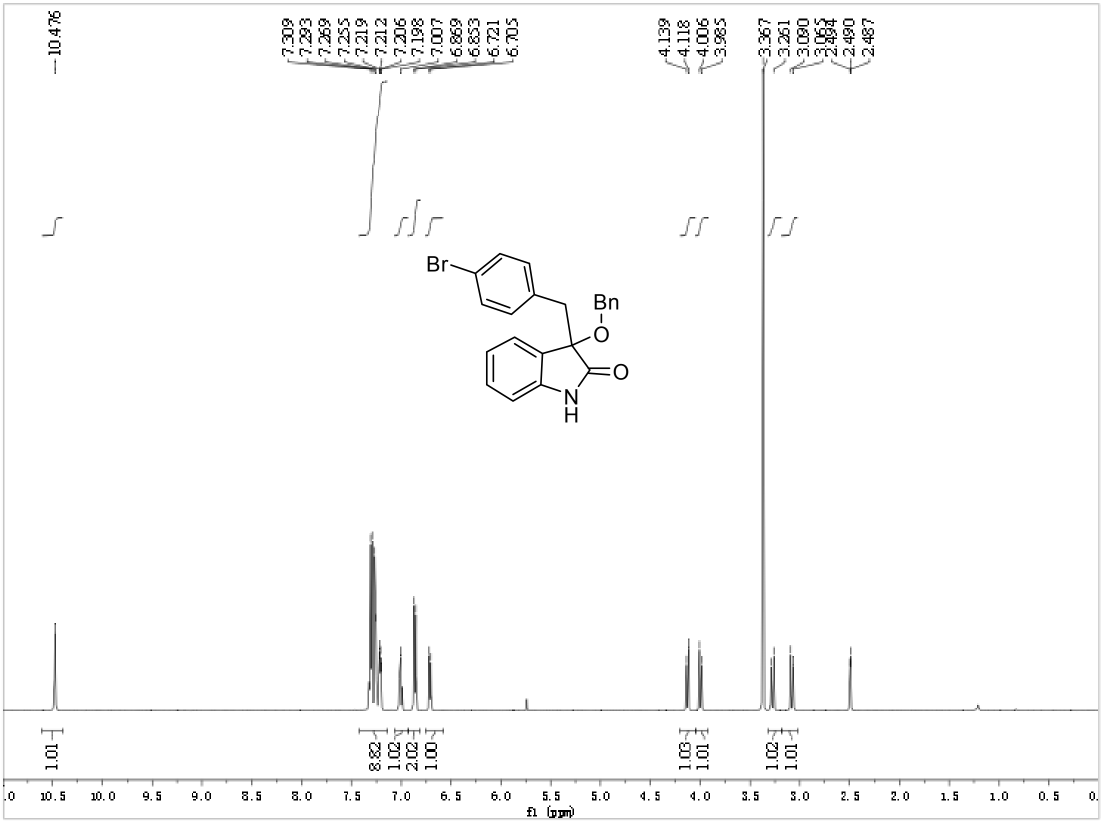

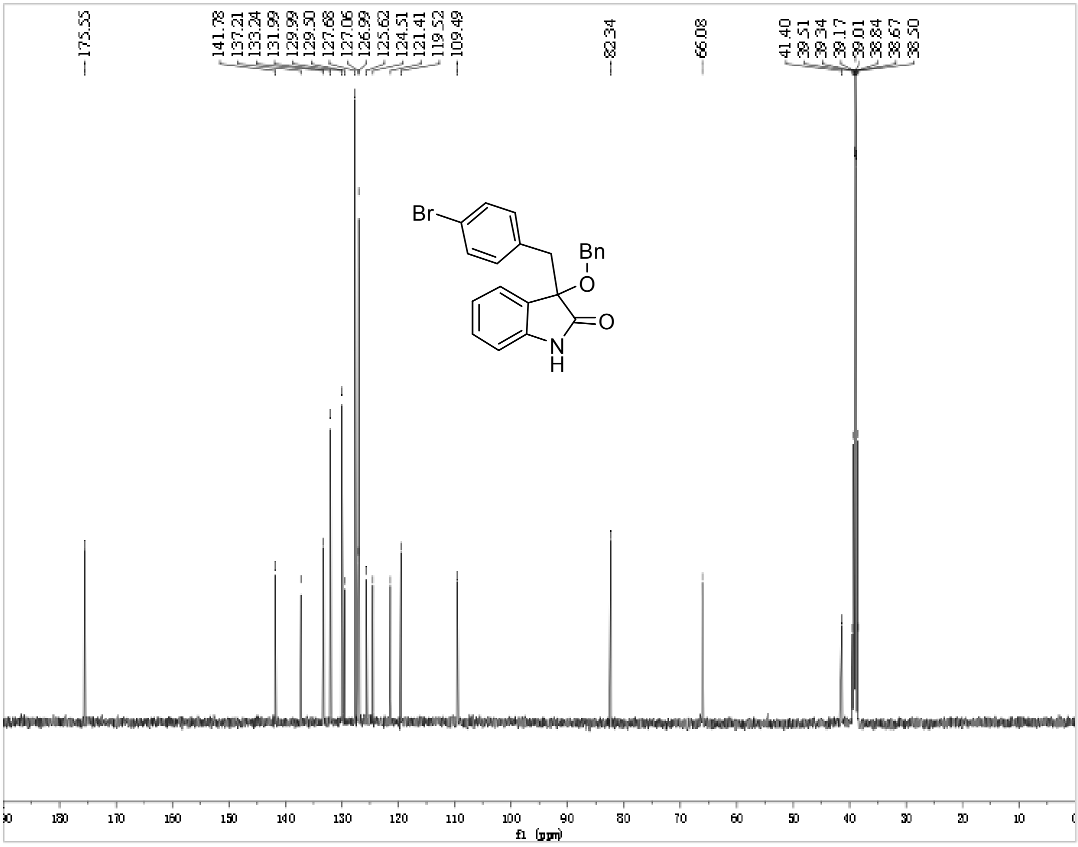

^{1}H and ^{13}C NMR of 3ef

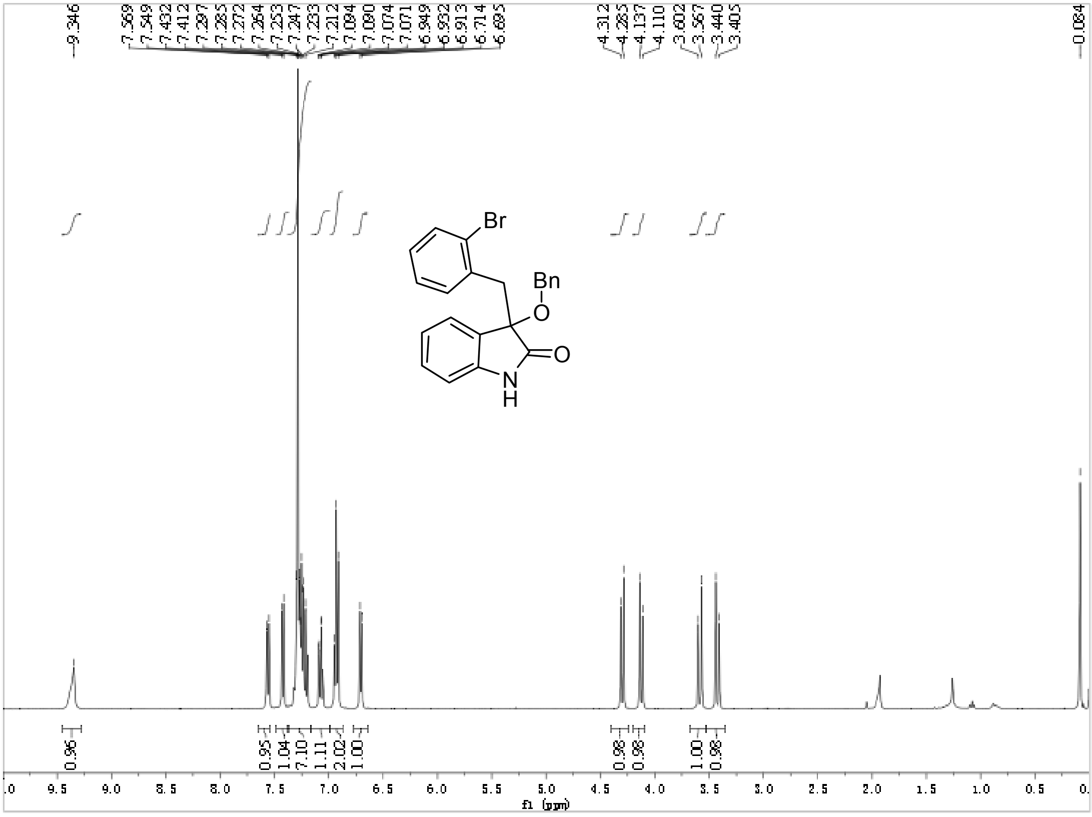

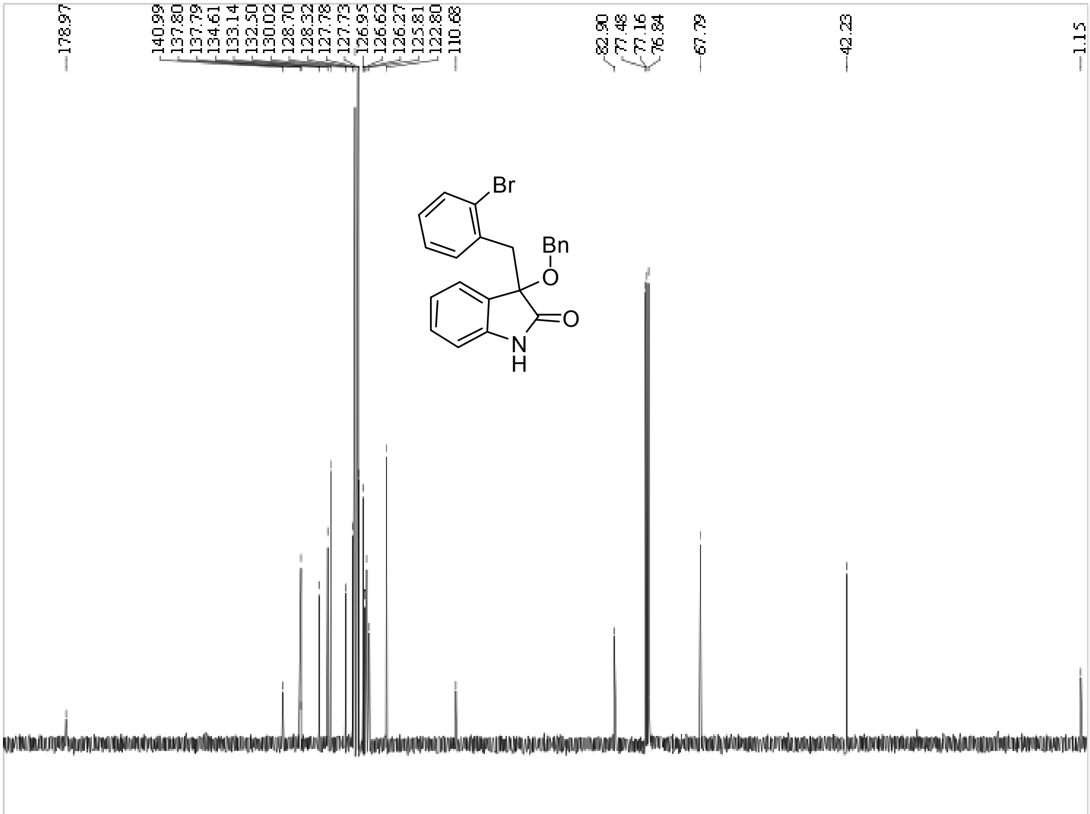

^{1}H and ^{13}C NMR of 3ff

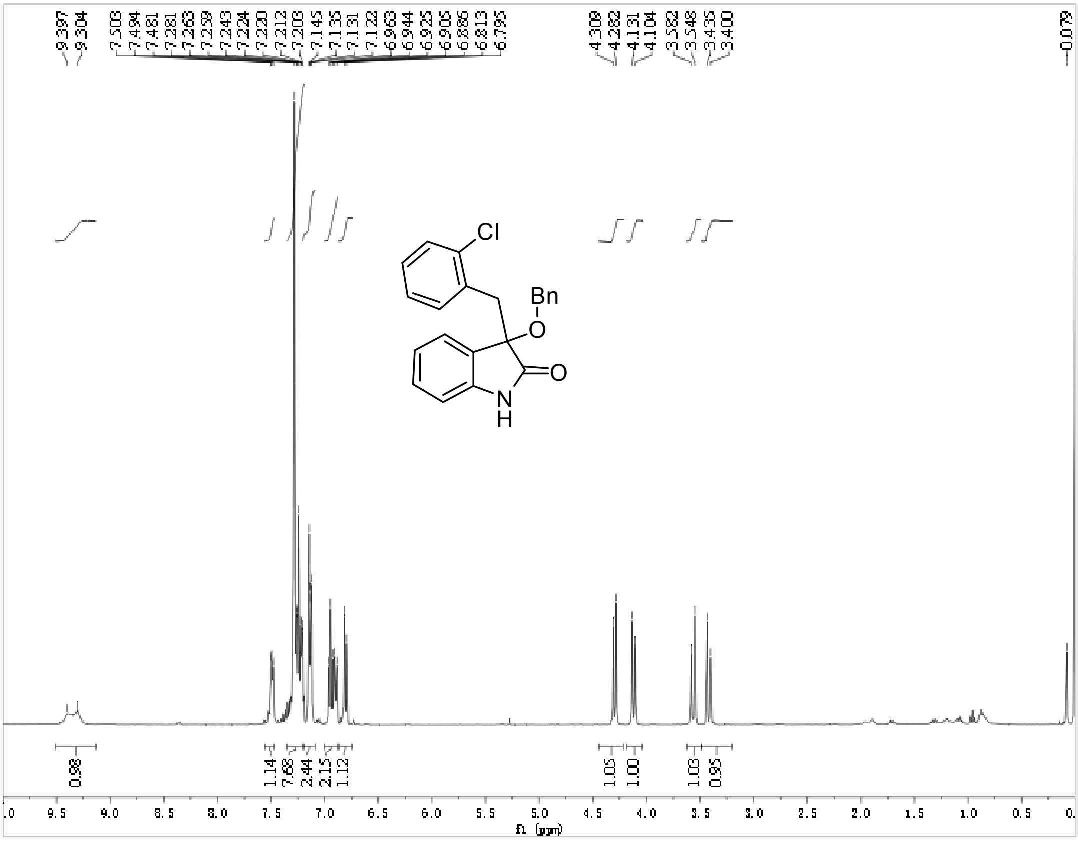

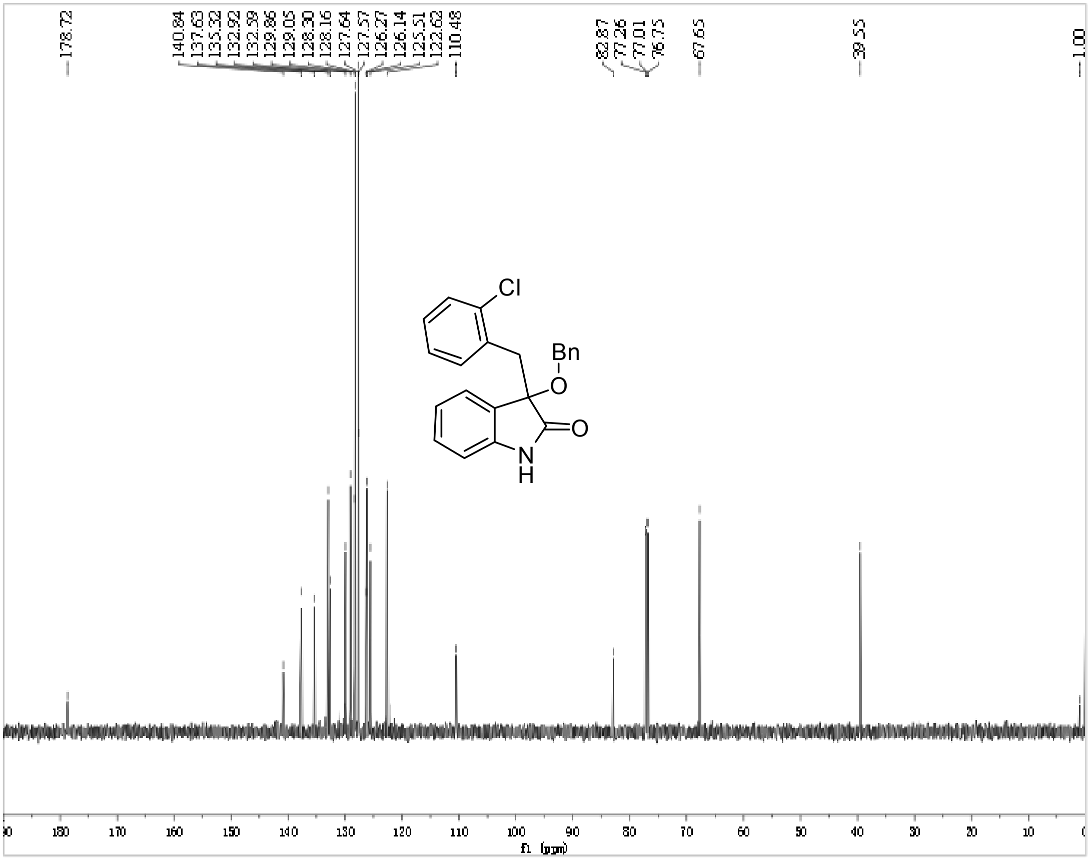

^{1}H and ^{13}C NMR of 3gf

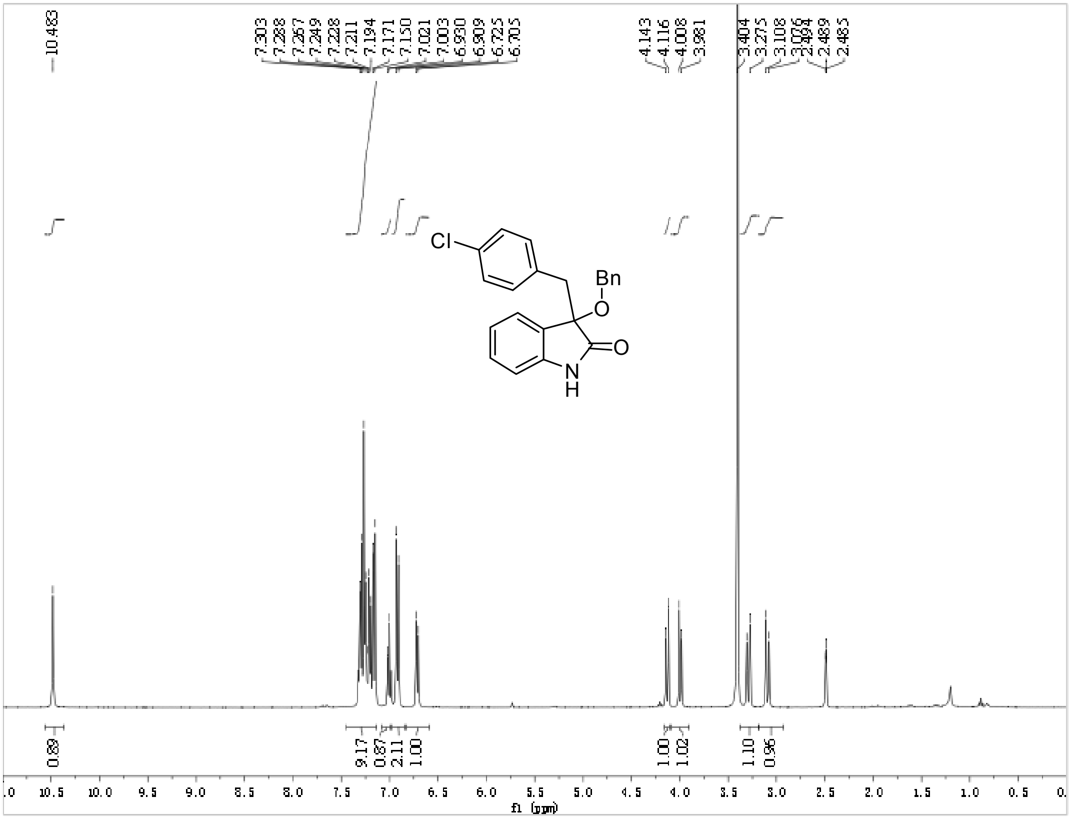

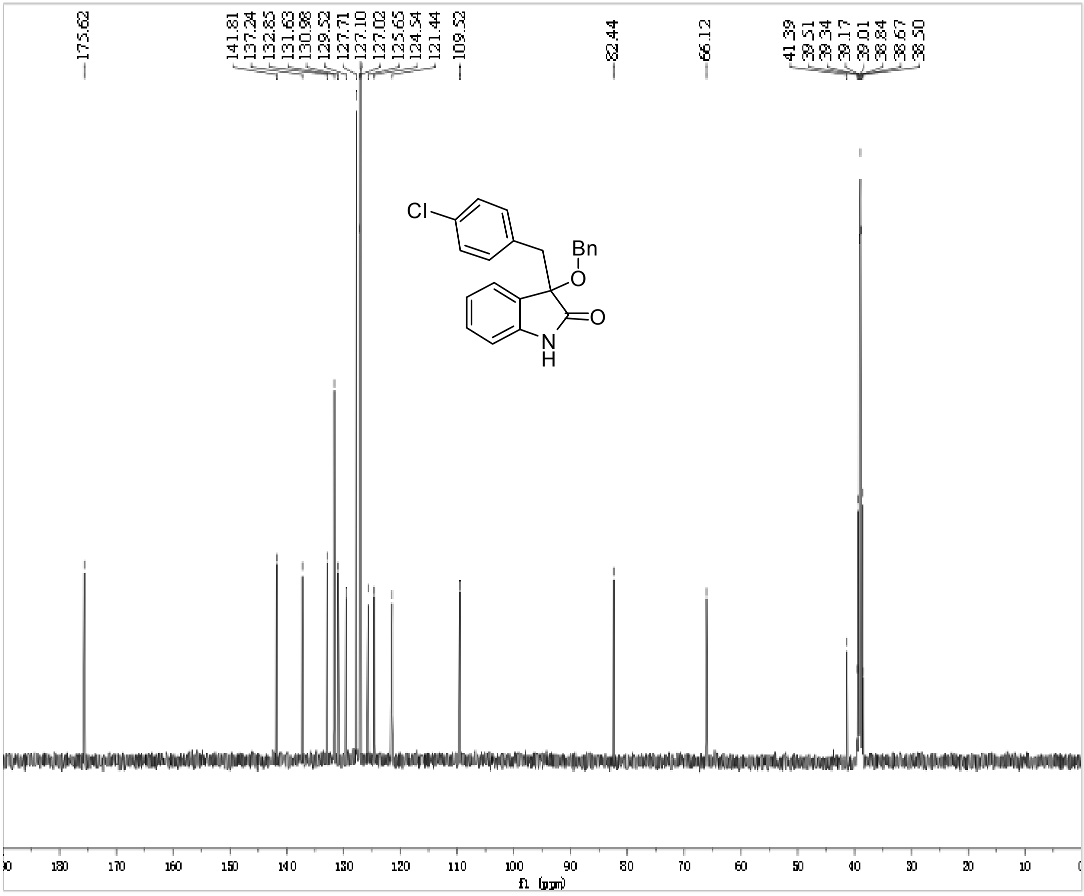

^1H and ^{13}C NMR of 3hf

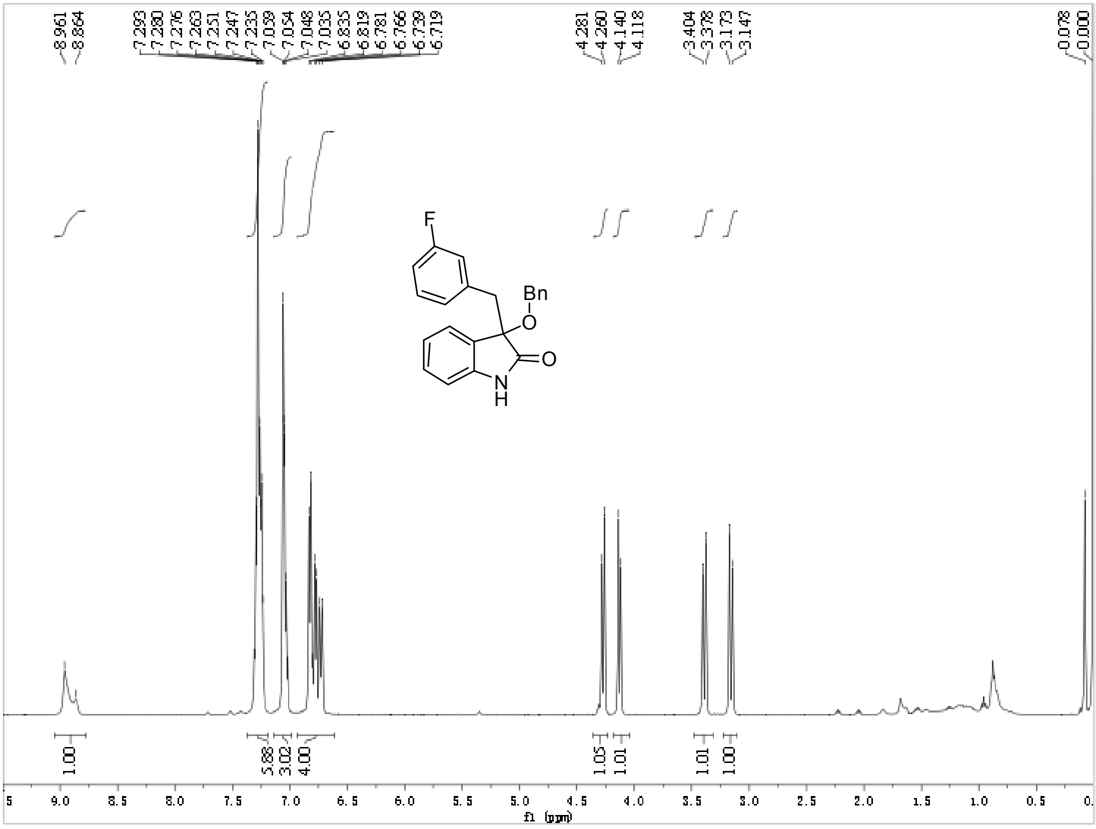

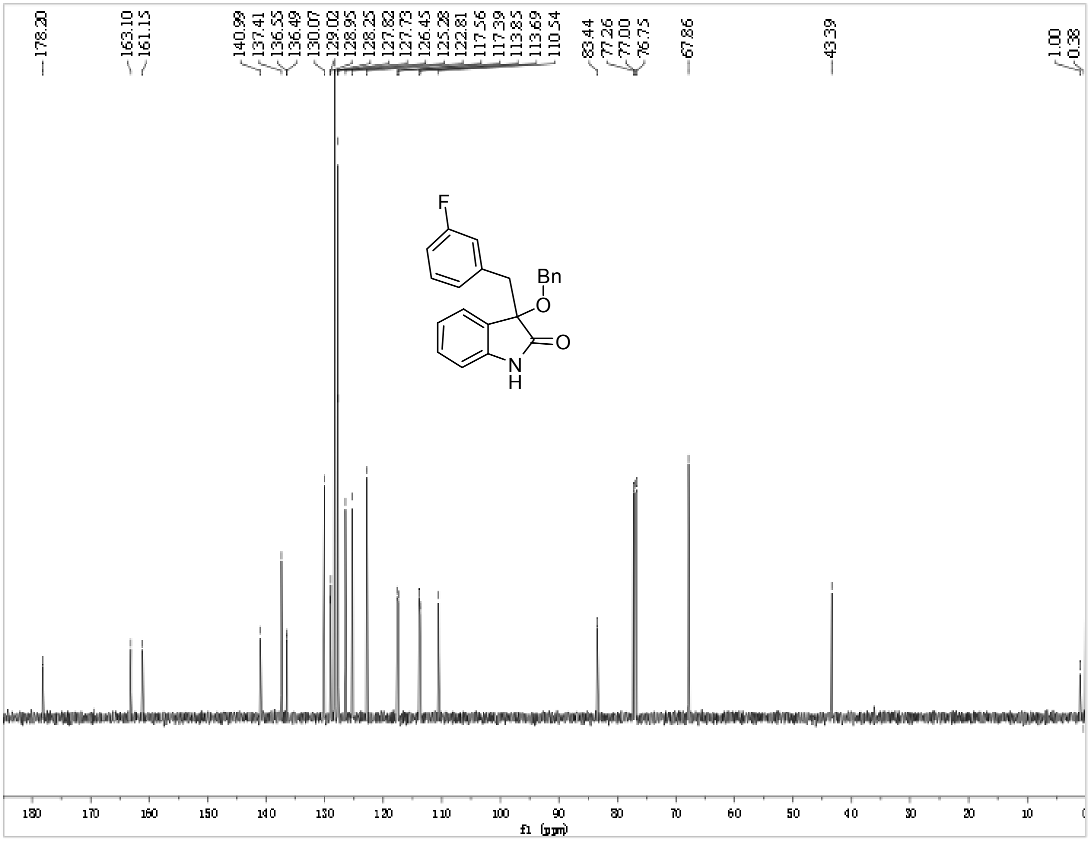

^{1}H and ^{13}C NMR of 3if

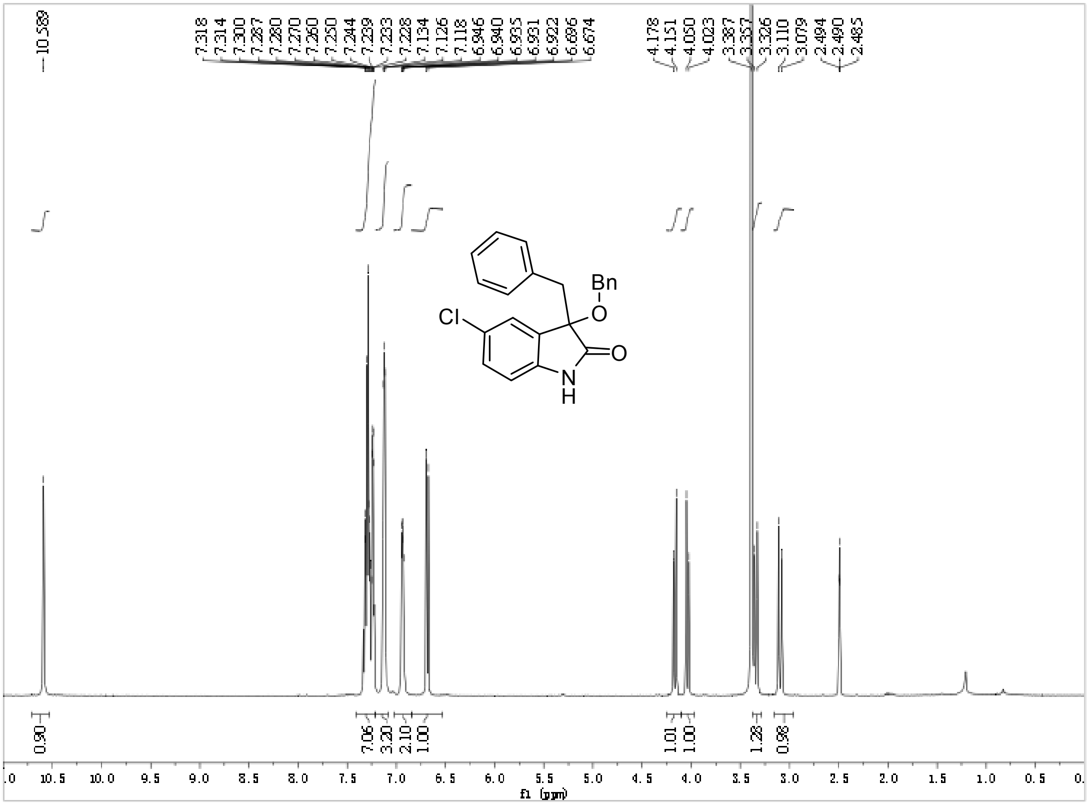

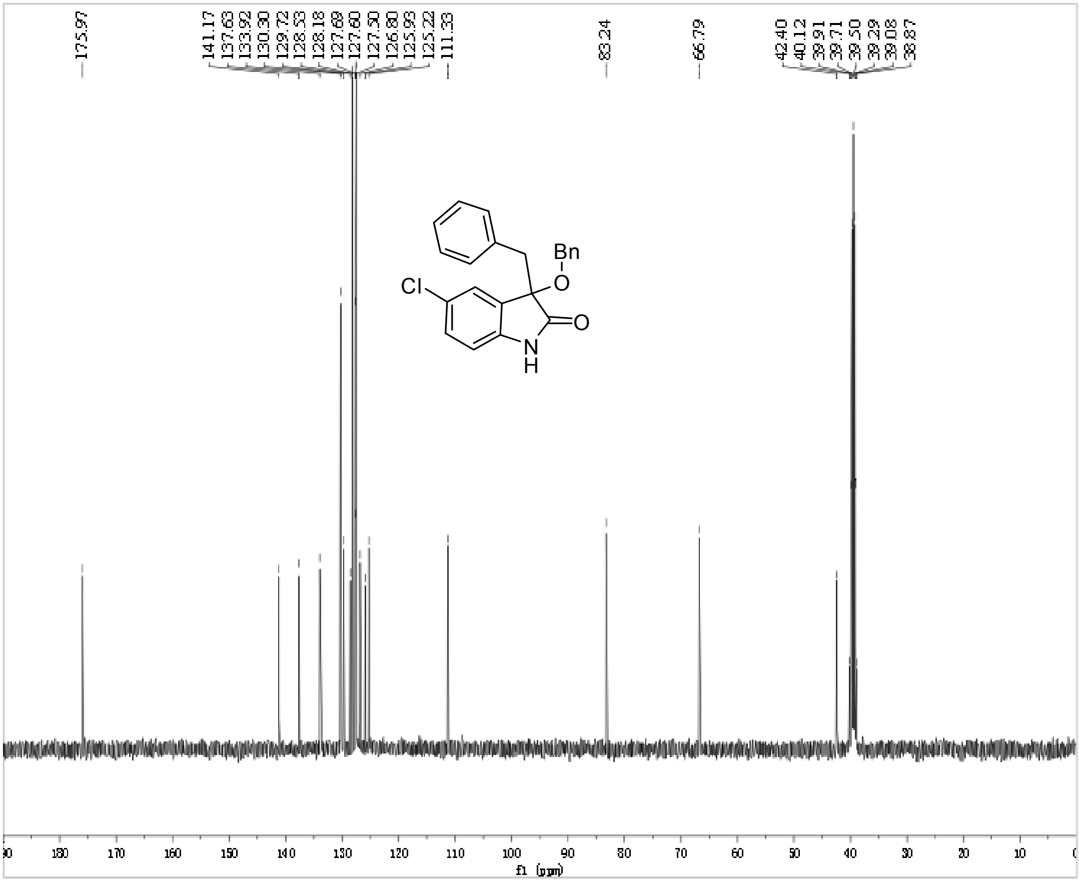

^{1}H and ^{13}C NMR of 3jf

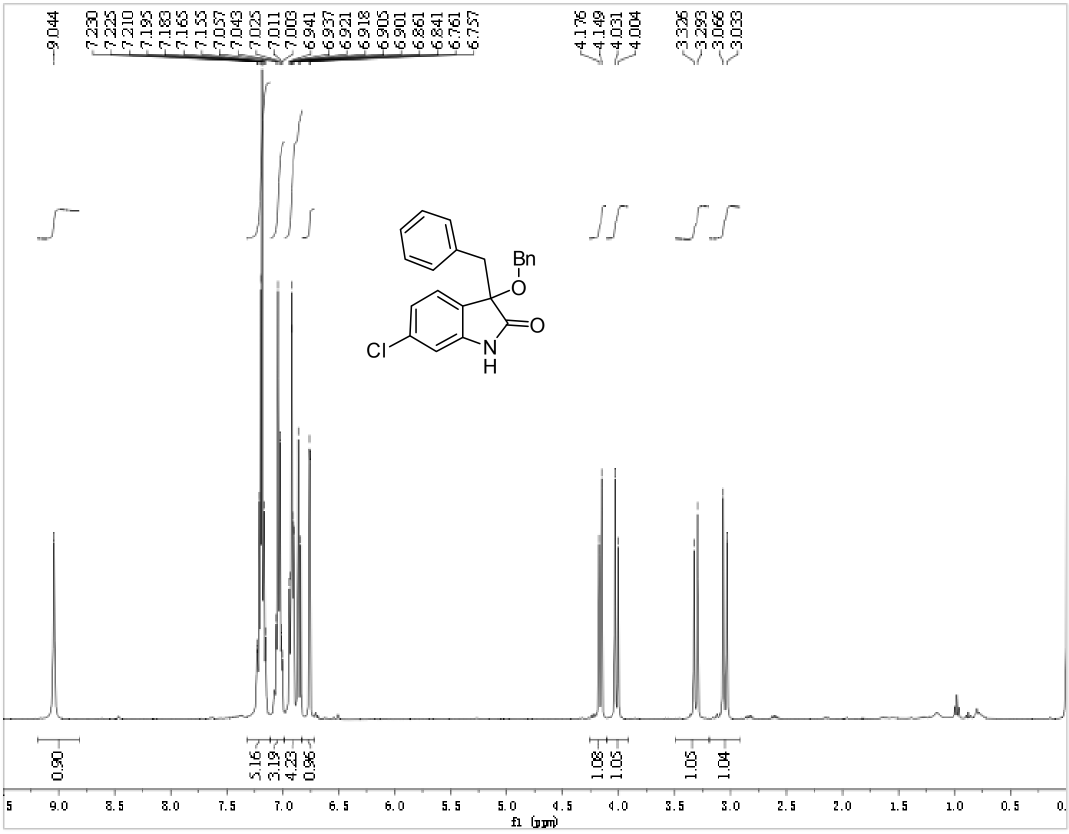

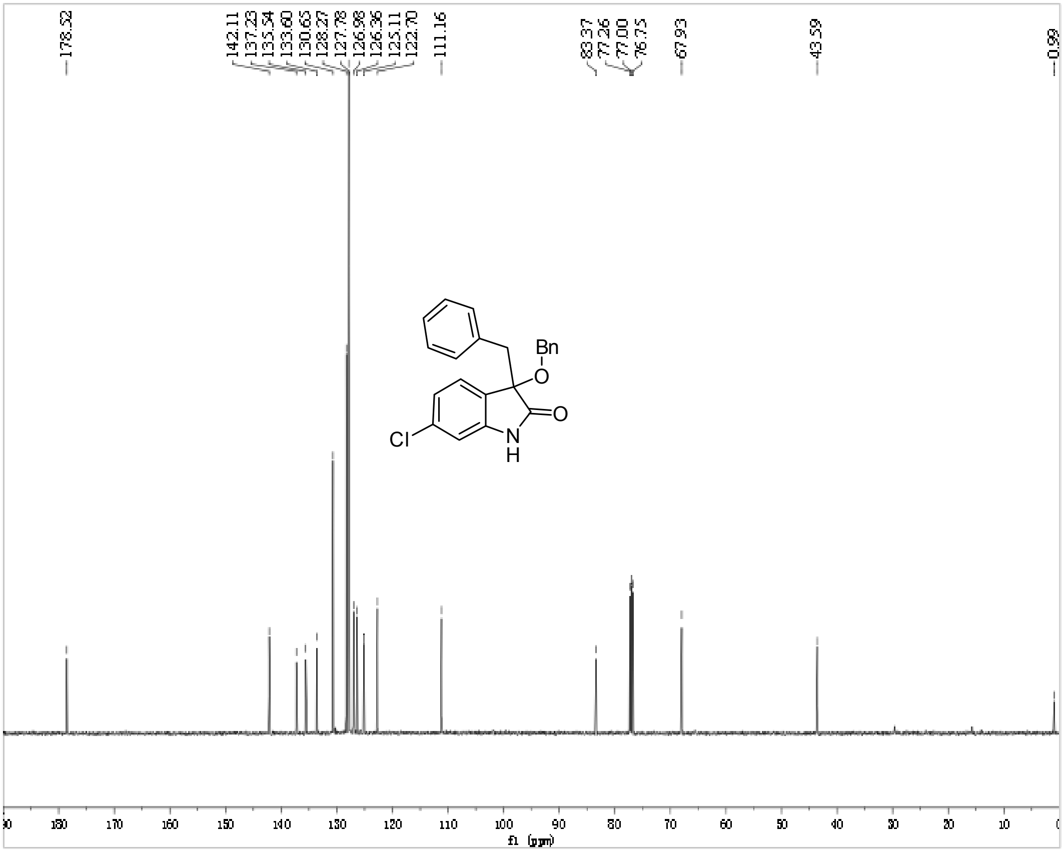

^{1}H and ^{13}C NMR of 3cg

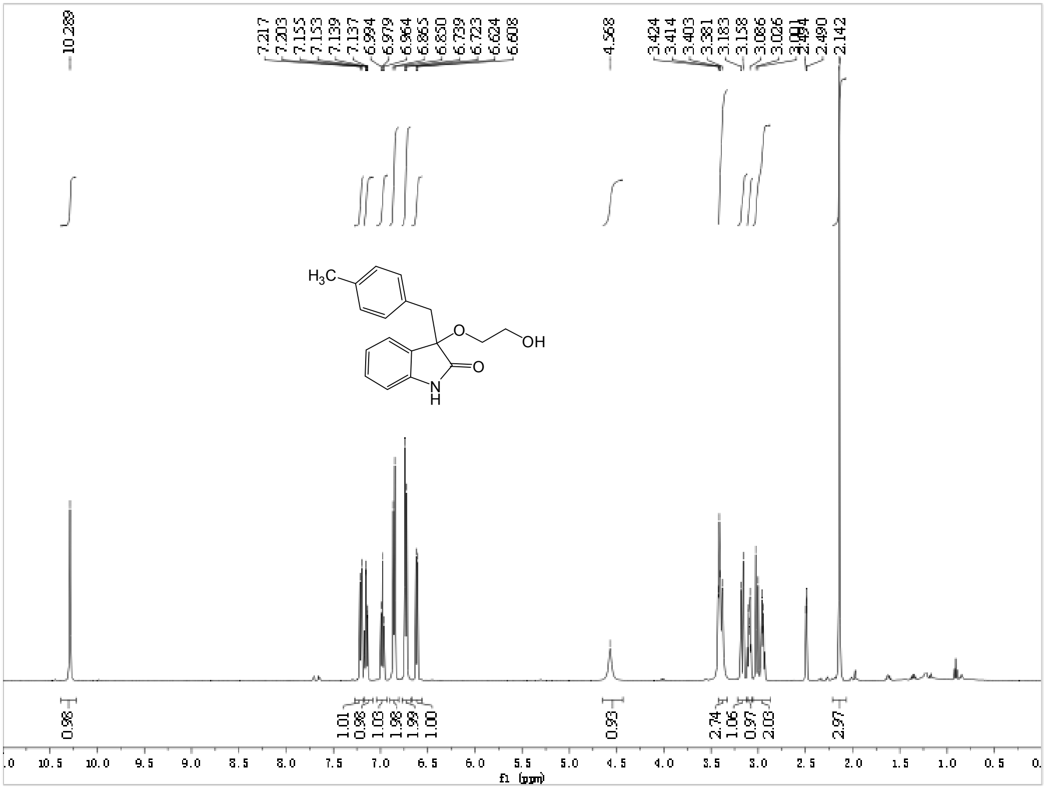

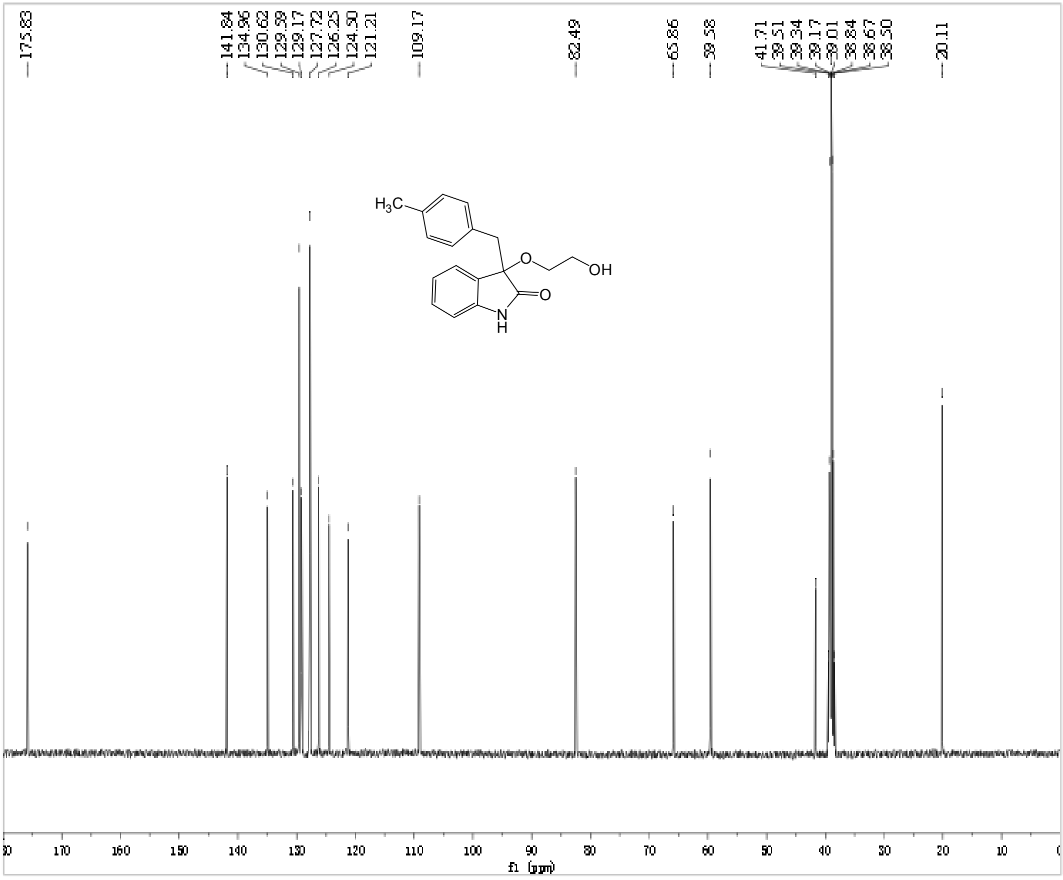

^{1}H and ^{13}C NMR of 3gg

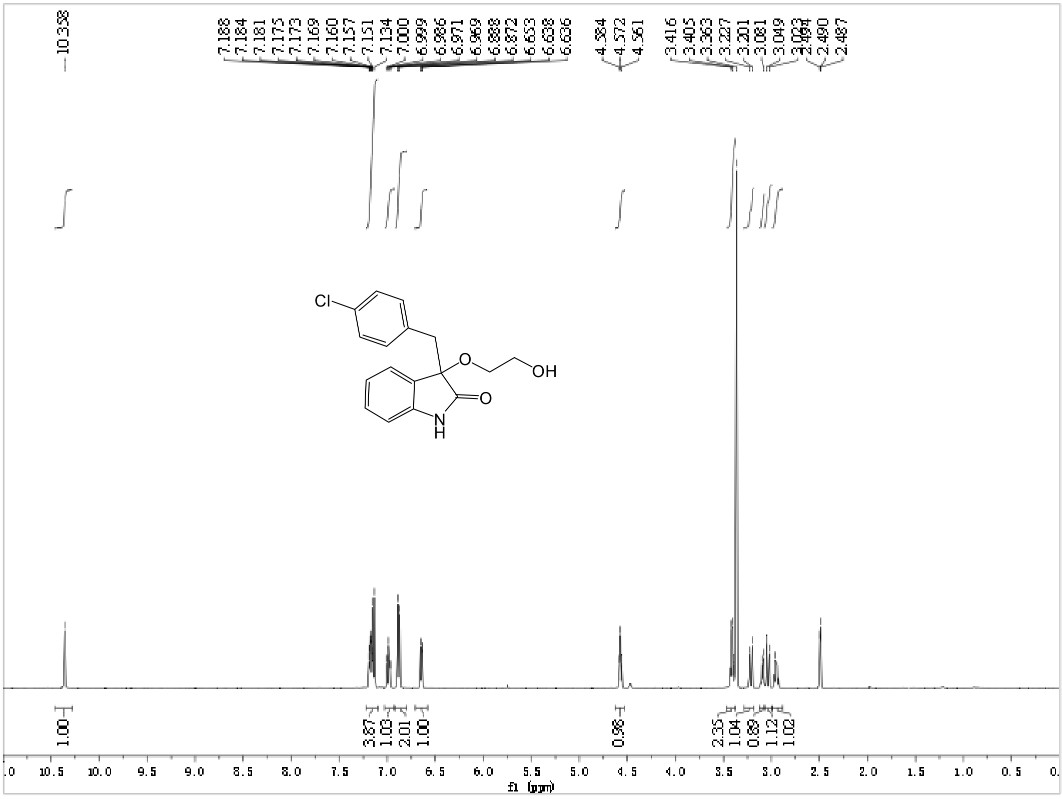

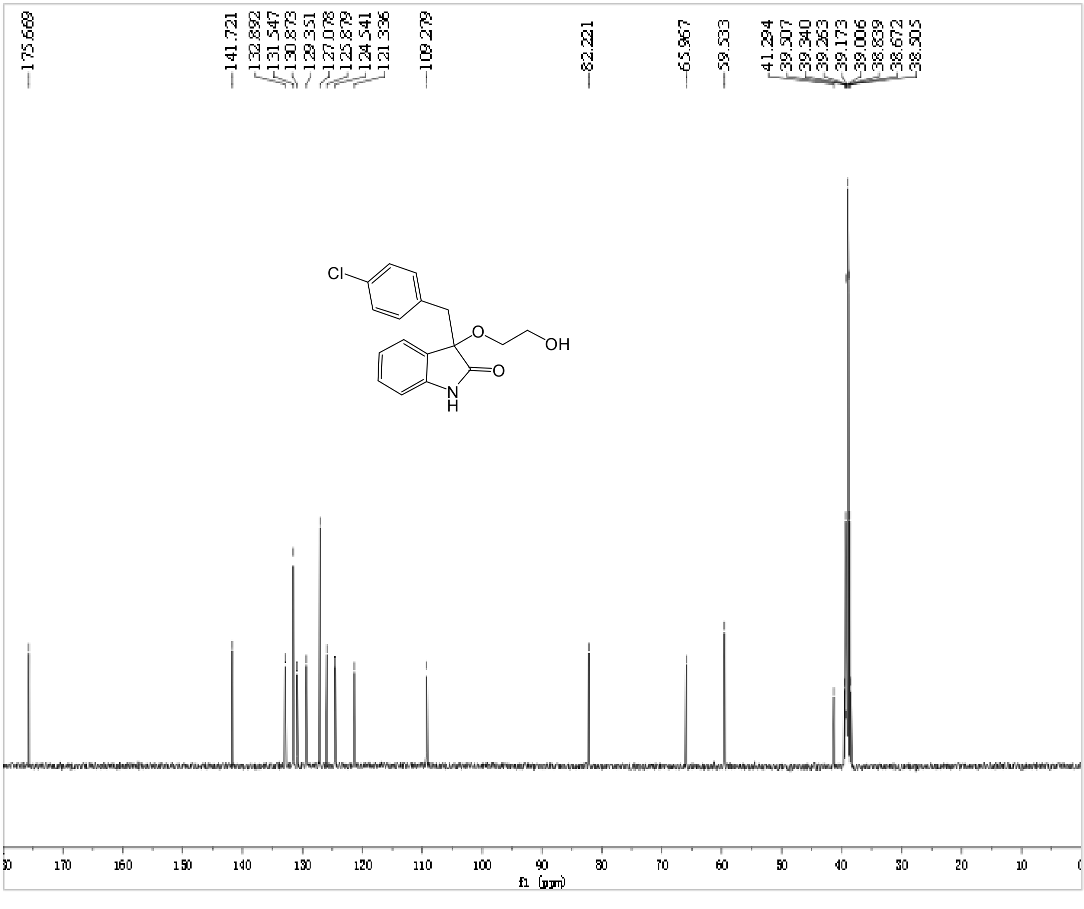

^{1}H and ^{13}C NMR of 3ah

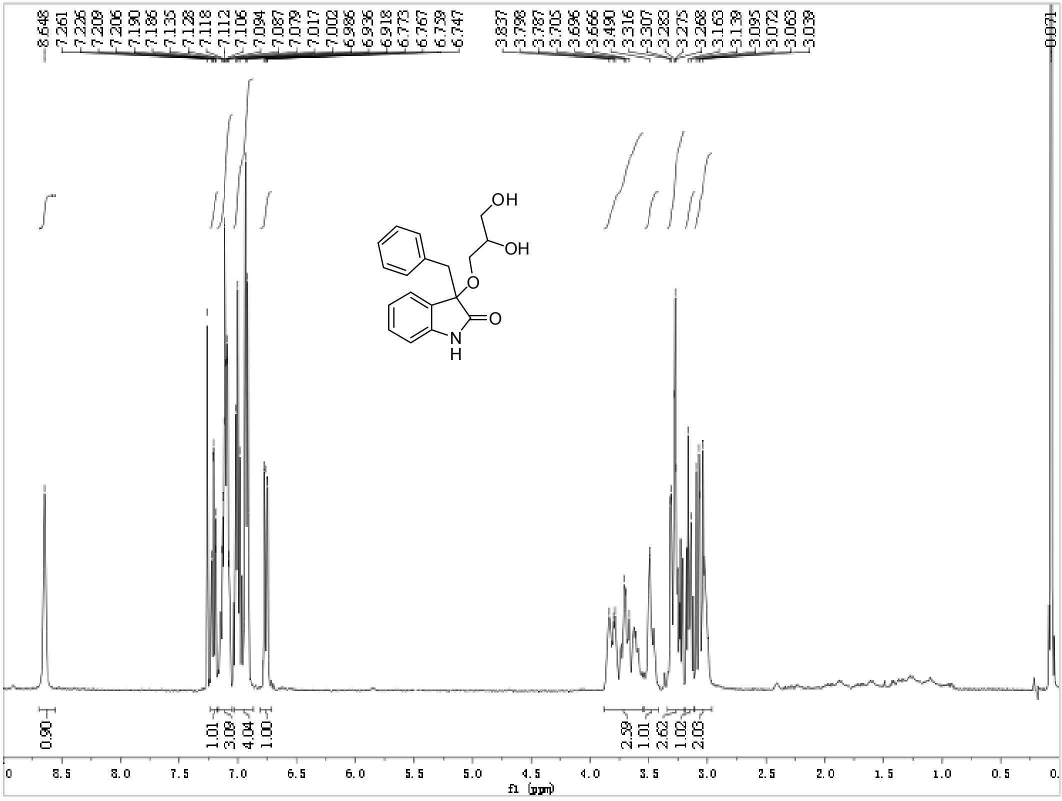

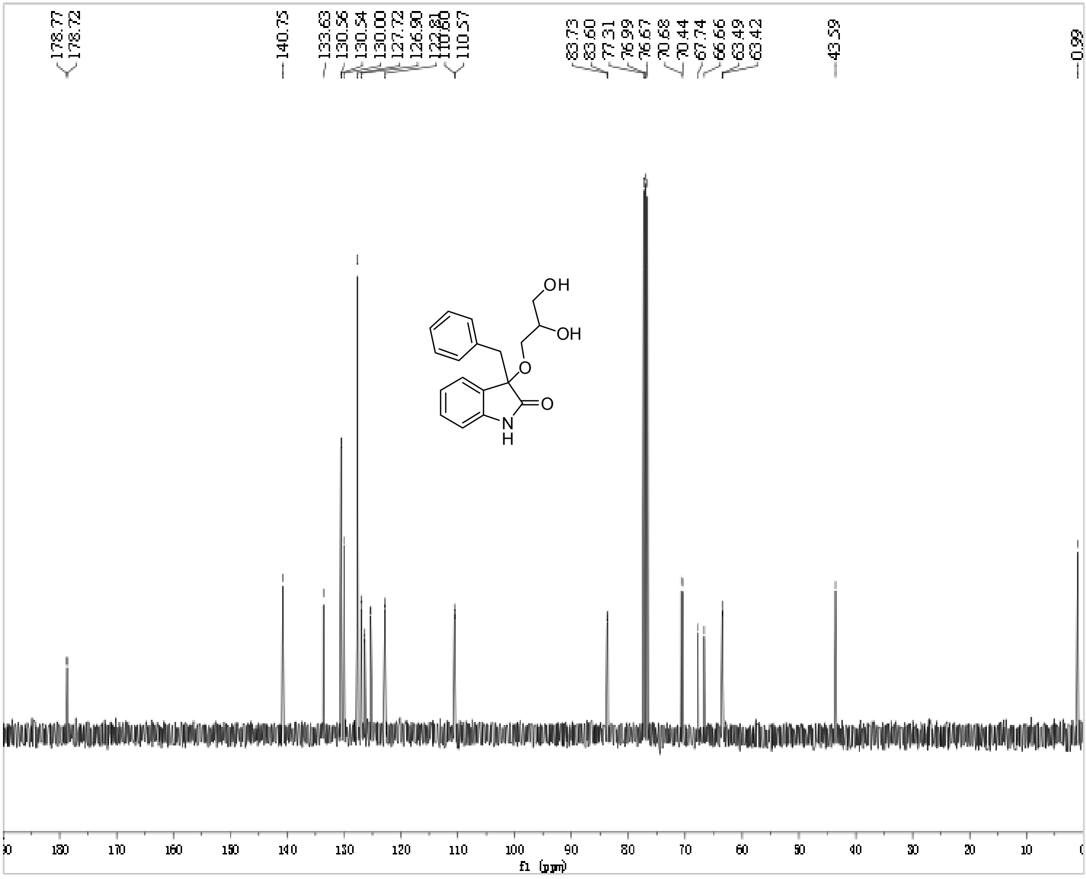

Supplement: Supplementary file 1 [file molecules-22-00801-s001.pdf]
